# eNRSA: a faster and more powerful approach for nascent transcriptome analysis

**DOI:** 10.1093/gigascience/giaf071

**Published:** 2025-07-04

**Authors:** Jing Wang, Hua-chang Chen, Scott W Hiebert, Quanhu Sheng, William P Tansey, Yu Shyr, Qi Liu

**Affiliations:** Department of Biostatistics, Vanderbilt University School of Medicine, Nashville, TN 37203, USA; Center for Quantitative Sciences, Vanderbilt University Medical Center, Nashville, TN 37203, USA; Department of Biostatistics, Vanderbilt University School of Medicine, Nashville, TN 37203, USA; Center for Quantitative Sciences, Vanderbilt University Medical Center, Nashville, TN 37203, USA; Department of Biochemistry, Vanderbilt University School of Medicine, Nashville, TN 37205, USA; Vanderbilt-Ingram Cancer Center, Vanderbilt University Medical Center, Nashville, TN 37232, USA; Department of Biostatistics, Vanderbilt University School of Medicine, Nashville, TN 37203, USA; Center for Quantitative Sciences, Vanderbilt University Medical Center, Nashville, TN 37203, USA; Department of Biochemistry, Vanderbilt University School of Medicine, Nashville, TN 37205, USA; Department of Cell and Developmental Biology, Vanderbilt University School of Medicine, Nashville, TN 37240, USA; Department of Biostatistics, Vanderbilt University School of Medicine, Nashville, TN 37203, USA; Center for Quantitative Sciences, Vanderbilt University Medical Center, Nashville, TN 37203, USA; Department of Biostatistics, Vanderbilt University School of Medicine, Nashville, TN 37203, USA; Center for Quantitative Sciences, Vanderbilt University Medical Center, Nashville, TN 37203, USA

**Keywords:** nascent transcriptome analysis, adaptive major transcript, alternative transcription start site (ATSS), alternative transcription termination site (ATTS), transcription readthrough

## Abstract

Nascent RNA sequencing tracks primary transcriptional events, making it crucial for studying the immediate regulatory changes of genes and enhancers in response to both endogenous and exogenous stimuli. NRSA is a widely used tool for analyzing nascent transcriptomic data, enabling quantification of transcriptional changes at proximal promoters and gene bodies, estimation of pausing indices, identifying active enhancers, and establishing enhancer–target gene relationships. To improve its functionality and broaden its applicability to diverse organisms and complex study designs, we have developed an enhanced version, eNRSA. Key advancements include adaptive selection of major transcripts, support for any organism with known gene structures, compatibility with complex study designs, and identification of alternative transcription start and termination sites, as well as transcription readthrough events. Additionally, eNRSA achieves a ∼20-fold increase in analysis speed while significantly reducing memory usage. These enhancements make eNRSA a faster, more versatile, and more powerful tool for nascent transcriptome analysis. eNRSA is freely available at https://bioinfo.vanderbilt.edu/eNRSA/.

## Introduction

Transcription is a highly regulated process comprising multiple stages, each precisely controlled to ensure accurate gene expression [1–4]. These key transcriptional stages include initiation, pausing, elongation, and termination [4]. Unlike steady-state RNA sequencing, nascent RNA sequencing captures transcription that is actively synthesized, providing a direct measure of gene expression across various regulatory stages [5, 6]. This capability is particularly valuable for uncovering immediate, direct, and transient transcriptional changes that reflect cellular responses to diverse conditions, including stress, differentiation, or disease progression.

**Figure 1:**
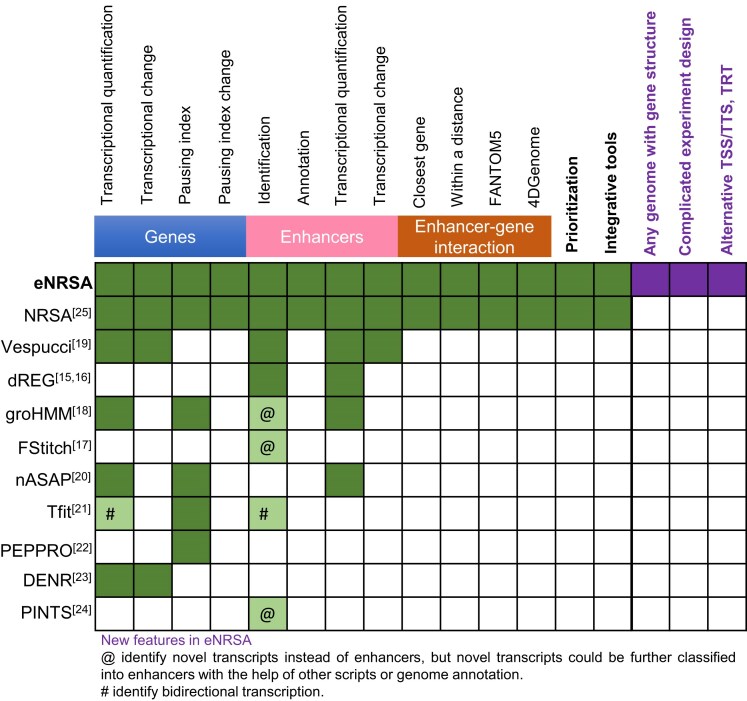
Summary of features distinguishing eNRSA from exiting nascent RNA sequencing analysis tools.

There are several sequencing-based techniques designed to extract newly transcribed RNAs from the total pool of cellular RNA. These include small capped RNA sequencing (Start-seq) [7], chromatin-associated RNA sequencing (caRNA-seq) [8, 9], global run-on sequencing (GRO-seq) [5], precision run-on sequencing (PRO-seq) [6], native elongating transcript sequencing (NET-seq) [10], mammalian NET-seq (mNET-seq) [11], thiol(SH)-linked alkylation for the metabolic sequencing of RNA (SLAM-seq) [12], bulk analysis of nascent transcript termini sequencing (Butt-seq) [13], and transient transcriptome sequencing (TT-seq) [14]. Of these, GRO-seq and PRO-seq are among the most widely used methods, largely due to their ability to provide high-resolution, genome-wide data on actively transcribing RNA polymerases. To analyze GRO/PRO-seq data, several tools have been developed, including dREG, FStitch, groHMM, Vespucci, nASAP, Tfit, NRSA, PEPPRO, DENR, and PINTS (Fig. 1). dREG [15, 16] and FStitch [17] focus on identifying active regulatory elements, such as enhancers with divergent transcription. groHMM [18] quantifies nascent transcription for both known genes and enhancers, while Vespucci [19] also estimates transcriptional changes between conditions. nASAP [20] is a web server for nascent RNA analysis, including transcription-level quantification, pausing site identification, and regulatory network construction. Tfit [21] aims to identify and profile bidirectional transcription sites using a finite mixture model. PEPPRO [22] is designed for quality control and preprocessing, and it also generates bigWig signal tracks for downstream analysis. DENR [23] models nascent reads as a mixture of user-provided isoforms, allowing it to estimate RNA abundance at both isoform and gene levels. PINTS [24] is a peak identifier that detects active promoters and enhancers genome-wide and pinpoints the precise location of 5′ transcription start sites. Among these tools, NRSA [25] stands out for its comprehensive analysis of nascent transcription. It not only quantifies nascent transcription and pausing for known genes but also detects, annotates, quantifies, and prioritizes active enhancers (Fig. 1). However, NRSA is time- and memory-intensive, particularly when processing large-scale nascent transcriptomes. Additionally, its limitation to single-factor designs and the requirement to preprocess gene structure files by selecting 1 major transcript per gene diminish its performance and restrict its applicability across diverse organisms and genomes.

Here, we developed enhanced NRSA (eNRSA) to significantly improve computational efficiency, broaden its applicability, and enhance performance (Fig. 1). eNRSA runs 20 times faster than NRSA while requiring only roughly one-eighth of the memory. Further, eNRSA enhances performance by adaptively selecting major transcripts based on the nascent transcriptome being analyzed and by supporting multifactor experimental designs. These advancements make eNRSA applicable to any organism or genome with a known gene structure. By fully leveraging the unique characteristics of the nascent transcriptome, eNRSA introduces new functionalities to identify alternative transcription start sites (ATSSs), alternative transcription termination sites (ATTSs), and transcription readthrough (TRT) dysregulation across conditions. eNRSA is freely available at [26].

## Results

### Overview of eNRSA

Nascent RNA sequencing captures the production of newly synthesized RNAs, offering a comprehensive view of regulatory dynamics throughout the transcription cycle, including initiation, pausing, elongation, and termination [4, 27]. In 2018, we developed NRSA, which enables in-depth analysis of the nascent transcriptome at both gene and enhancer levels, surpassing other tools in scope and accuracy [25]. NRSA not only estimates promoter-proximal pausing and elongation rates but also identifies, annotates, and quantifies active enhancers while measuring enhancer-mediated regulation.

**Figure 2:**
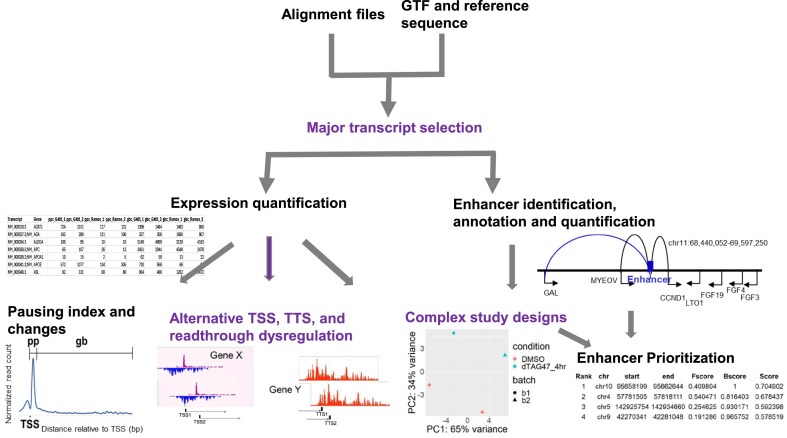
Workflow of eNRSA.

To enhance NRSA’s performance and broaden its applications, we developed an advanced version, eNRSA, which supports any organism with a known gene structure; accommodates complex study designs; introduces new functions to identify ATSSs, ATTSs, and readthrough dysregulation; and significantly improves computational efficiency (Fig. 1). eNRSA takes nascent transcriptome data and a reference genome with a gene structure file as input, providing detailed outputs on transcriptional changes in promoter-proximal and gene body regions, alternative transcriptional events (including ATSSs, ATTSs, and readthrough), enhancer activity, and the pausing index, along with various visualization options (Fig. 2).

eNRSA introduces 3 key advancements: (i) adaptive selection of major transcripts, enabling data-driven definition of promoter-proximal and gene body regions, extending support to any organism with defined gene structures (compared to NRSA’s limitation of 5 organisms and 8 genomes); (ii) advanced capabilities for complex study designs, improving differential transcriptional analysis by accounting for confounding factors and allowing for multifactorial designs; and (iii) new functions to identify ATSSs, ATTSs, and readthrough dysregulation (Fig. 2). Additionally, eNRSA significantly optimizes computational performance by simultaneously increasing processing speed and reducing memory requirements.

### eNRSA selects major transcripts adaptively and supports any organism with a known gene structure

One primary function of NRSA is to quantify transcriptional changes at promoter-proximal and gene body regions and to estimate the pausing index, which heavily depends on selecting the major transcript for each gene. Given that a single gene often encodes multiple transcripts, selecting the major transcript is crucial for accurately representing the gene’s nascent transcription. This selection significantly impacts the definition of the corresponding promoter-proximal and gene body regions, ultimately influencing the accuracy of transcriptional regulation quantification. To simplify calculations, NRSA uses the longest transcript for each gene as the major transcript, defining promoter-proximal and gene body regions accordingly. Although NRSA preprocesses the gene structure file (GTF file) to extract the longest transcript for each gene from 8 genomes across 5 organisms (hg19, hg38, mm10, mm39, dm3, dm6, ce10, and danRer10) and packs these preprocessed files in the tool for seamless analysis, users have to perform this preprocessing themselves if working with other organisms or different genome versions. This task is nontrivial and demands programming skills, limiting NRSA’s broad application to various organisms. Furthermore, this approach may yield misleading findings if the longest transcript is not actually the major transcript for a given gene.

To address these issues, eNRSA automatically and adaptively selects major transcripts based on the nascent transcriptome data being analyzed. Unlike NRSA, which relies on preselected longest transcripts, eNRSA identifies transcripts with the highest nascent transcriptional levels as major transcripts. This data-driven approach is more accurate and flexible than a fixed selection method, as major transcripts may vary under different conditions. By removing the preprocessing step, eNRSA supports any organism with a known gene structure without additional procedures.

### eNRSA improves differential transcription analysis by allowing complex study designs

Most nascent transcriptome analysis tools, including NRSA, are limited to handling simple study designs involving only 2 groups separated by a single factor of interest [19, 25], lacking the ability to analyze complex study designs with multiple factors or confounding variables, such as batch effects. This limitation restricts NRSA’s applicability and can result in unreliable and irreproducible findings. Failing to account for confounding factors can lead to either false differential transcription arising from technical rather than biological effects or loss of true differential signals.

**Figure 3:**
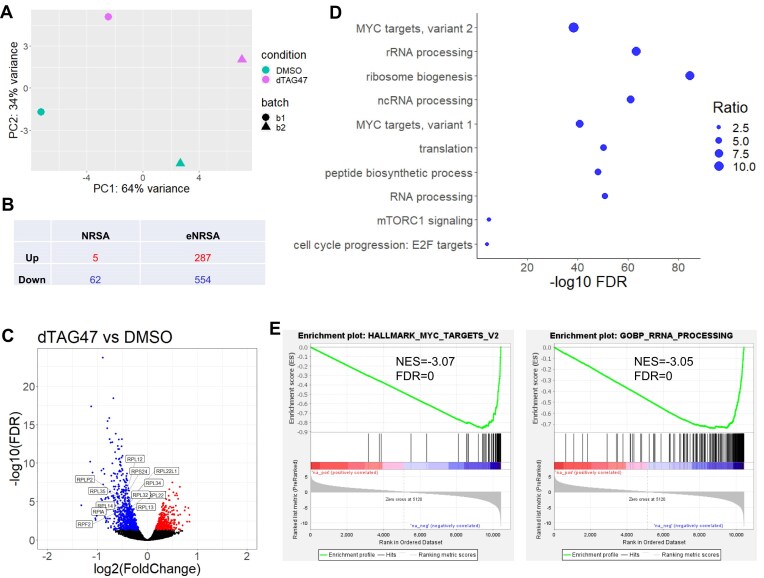
eNRSA enhances differential analysis by removing the batch effect. (A) PCA plot of normalized counts mapped to the gene body for DMSO- and dTAG47-treated cells from 2 batches. (B) The number of dysregulated genes identified by NRSA and eNRSA with an FDR <0.05. (C) Volcano plot showing the log_2_ fold change (x-axis) and −log_10_ FDR (y-axis) for dTAG47-treated vs. DMSO-treated cells on gene body transcription. (D) Pathways enriched in the downregulated genes in dTAG47-treated vs. DMSO-treated cells by WebGestalt 2024. (E) GSEA results revealing the 2 most significant pathways, MYC targets and rRNA processing, enriched in the downregulation of dTAG47-treated cells compared to DMSO.

For example, when NRSA was used to compare the nascent transcriptome between 2 conditions—DMSO-treated and dTAG47-treated cells for MYC binding depletion [28]—it identified only 5 upregulated and 62 downregulated genes with an false discovery rate (FDR)<0.05 and |log_2_ fold change| >0.3, with no notable pathways emerging from functional enrichment analysis. Principal component analysis (PCA), however, revealed a strong batch effect, with DMSO-treated and dTAG47-treated cells from the same batch clustering even more closely than cells from the same condition in different batches (Fig. 3A). The lack of batch effect correction likely explains why NRSA did not yield biologically meaningful results.

In contrast, eNRSA supports the analysis of complex study designs, with the ability to adjust for confounding factors, thereby enhancing differential transcription analysis. When applied to the same dataset of DMSO-treated and dTAG47-treated cells with the batch factor incorporated in the model, eNRSA identified 841 dysregulated genes with an FDR <0.05 and |log_2_ fold change| >0.3, including 287 upregulated and 554 downregulated ones (Fig. 3B). To further evaluate the performance of eNRSA, we permuted PRO-seq reads between DMSO- and dTAG47-treated cells (100 permutations) to generate a background distribution, which was then used to estimate the probability of obtaining the observed difference by chance. All 841 genes were supported by the permutation-based test. Using this test as the gold standard, eNRSA achieved 80.1% sensitivity, 100% specificity, and 95.6% accuracy.

Notably, most downregulated genes in dTAG47-treated cells were either MYC targets or associated with core MYC functions. MYC is known to control the transcription of genes essential for ribosome biogenesis [29–33]. Consistently, eNRSA detected significant downregulation of ribosome-related genes in dTAG47-treated cells, including *RPLP2, RPL12, RPL13, RPL14, RPL32, RPL34, RPL35*, and *RPS24* (Fig. 3C; Supplementary Table S1). Functional enrichment analysis using WebGestalt [34] and GSEA [35] revealed significant downregulation of genes involved in ribosome biogenesis, mTORC1 signaling, and MYC targets (normalized enrichment score [NES] = −3.07, FDR = 0), as well as rRNA processing (NES = −3.05, FDR = 0) (Fig. 3D and E; Supplementary Table S2). These findings demonstrate that eNRSA effectively unmasks true differential transcription signals previously obscured by batch effects, significantly enhancing detection power.

### eNRSA identifies ATSSs, ATTSs, and readthrough dysregulation

In mammalian genomes, most genes give rise to multiple transcript isoforms. At least 70% of genes have multiple polyadenylation sites, more than 50% have alternative transcription start sites, and transcripts from nearly all genes can be subject to alternative splicing [36–39]. Differential transcript isoforms can encode products that differ in structure, location, stability, enzyme activity, and other properties, which regulate key biological processes and contribute to disease. Therefore, identifying these alternative events is crucial [36].

ATSSs and ATTSs have been reported to contribute more to isoform diversity than alternative splicing [36]. TSS denotes the annotated start of the gene, while TTS refers to the annotated end of the gene, defined as the 3′ end of the last exon in the GTF file, marking the cleavage and polyadenylation site of the mature RNA transcript. Nascent transcriptome data from GRO/PRO-seq, (m)NET-seq, and Butt-seq are characterized by peaks at promoter-proximal regions and an accumulation of reads around TTS sites, providing a natural way to identify ATSSs and ATTSs. However, no tools are currently available to specifically identify ATSSs and ATTSs from nascent transcriptome data. Leveraging these unique characteristics, eNRSA identifies ATSSs by detecting shifts in read distributions across promoter-proximal regions and ATTSs by detecting shifts across TTS sites, between 2 conditions. Each promoter-proximal region corresponds to a TSS site, where higher read counts indicate greater TSS usage. A shift in read enrichment from one TSS site to another signals an ATSS event, which eNRSA assesses using the Cochran–Mantel–Haenszel test (CMH) for statistical significance (details in Methods). Similarly, eNRSA detects ATTS events by counting and estimating shifts in read distributions across TTS sites.

**Figure 4:**
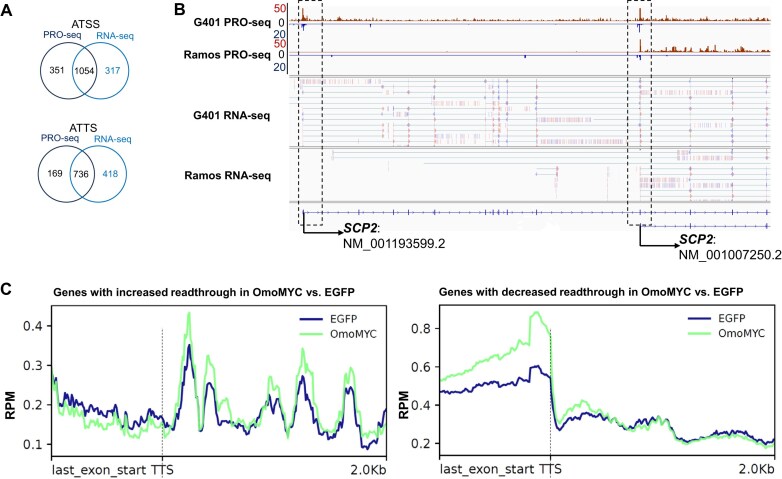
Example of ATSS and readthrough dysregulation identified by eNRSA. (A) ATSSs (up) and ATTSs (bottom) identified from PRO-seq and RNA-seq between G401 and Ramos cells. (B) IGV screenshot of PRO-seq and RNA-seq signals illustrating ATSS events between G401 and Ramos cells. In the G401 cell line, promoter-proximal peaks were observed at 2 TSS sites, while only a single peak was observed at the second TSS site in the Ramos cell line. (C) Metagene profiles of PRO-seq data for the genes with increased (left) and decreased (right) readthrough in OmoMYC vs. EGFP.

eNRSA demonstrated strong performance in detecting ATSS and ATTS events, with findings further supported by RNA-seq data. When applied to compare nascent transcriptome between 2 cell lines, Ramos and G401, eNRSA identified 1,405 ATSSs and 905 ATTSs at an FDR <0.05 (Supplementary Tables S3 and S4). These events were then compared to those detected from RNA-seq data. Given the complexity of transcriptional regulation and the fact that nascent transcriptome profiling and RNA-seq capture different layers of transcription, a strong overlap was observed between the 2 datasets. Specifically, among the 1,405 ATSSs, 1,054 (75.0%) were also supported by RNA-seq; likewise, among the 905 ATTSs, 736 (81.3%) were confirmed by RNA-seq (Fig. 4A; Supplementary Tables S3 and S4). As an illustrative example, the *SCP2* gene exhibited one of the most significant ATSS events (FDR = 0). In the G401 cell line, promoter-proximal peaks were observed at 2 TSS sites: 1 at chr1:53,392,901 (hg19), corresponding to the long transcript NM_001,193,599.2, and the other at chr1:53,480,610 (hg19), corresponding to the short transcript NM_001,007,250.2. In contrast, only a single peak was observed at the second TSS site (chr1:53,480,610) in the Ramos cell line (Fig. 4B). This shift in read enrichment—from 2 TSS sites in G401 to 1 in Ramos—clearly indicates an ATSS event. This event was validated by RNA-seq data from the same cell lines, where both long and short transcripts were detected in G401, but only the short transcript was present in Ramos (Fig. 4B). The differential TSS usage between these cell lines suggests that *SCP2* may produce distinct proteins with differing functions. According to Uniprot, the 2 *SCP2* transcripts encode distinct proteins through transcription initiation from independently regulated promoters. The long transcript encodes SCPx, a thiolase enzyme essential for peroxisomal oxidation of branched-chain fatty acids [40], while the short transcript encodes SCP2, an intracellular lipid transfer protein that facilitates the transfer of common phospholipids, cholesterol, and gangliosides from the endoplasmic reticulum to the plasma membrane [41–43].

From a Polymerase II (Pol II) perspective, transcription termination does not occur immediately at the cleavage site. Instead, Pol II continues transcribing downstream, and dissociation typically occurs 1–50 kb downstream [14, 44, 45]. Therefore, eNRSA defines “readthrough” as transcriptionally engaged Pol II detected downstream of the annotated TTS/cleavage site and extending to the Pol II dissociation zone. This readthrough signal is expected in nascent transcription assays such as PRO-seq, GRO-seq, NET-seq, mNET-seq, and Butt-seq, which capture engaged Pol II, or TT-seq, which also maps transient RNA downstream of polyadenylation sites [14]. In contrast, assays like SLAM-seq, which detect metabolically labeled RNAs posttranscriptionally, are less likely to show signal downstream of the cleavage site due to rapid degradation of unprocessed or unstable transcripts in the readthrough region [12]. Transcription readthrough is observed not only in various cellular stress conditions but also in healthy tissues, suggesting that readthrough transcripts may play a role in regulating cellular processes [46, 47]. To detect readthrough dysregulation, eNRSA calculates a readthrough ratio by comparing the number of reads within a fixed 50 kb downstream of the TTS relative to the number of reads in the terminal exon [44], then estimates the ratio change between 2 conditions to identify dysregulations (details in Methods). Using this approach, eNRSA identified 217 protein-coding genes with reduced readthrough and 160 with increased readthrough in engineered G401 cells expressing OmoMYC—a dominant-negative mutant that blocks the productive association of MYC with its target genes, compared to cells expressing inducible forms of enhanced green fluorescent protein (EGFP, control) (FDR <0.05; Supplementary Table S5). Metagene analysis of nascent transcription for genes with increased readthrough in OmoMYC G401 cells revealed consistent read accumulation beyond the TTS, whereas genes with decreased readthrough showed read depletion in this region (Fig. 4C).

Cleavage and polyadenylation (CPA) factors, including CPSF73, CstF64, and CstF64t, play crucial roles in the 3′ end processing of RNA and are essential for proper transcription termination. A previous study using mNET-seq profiling has shown that depletion of CPSF73 and the double knockdown of CstF64 and CstF64t proteins lead to termination defects [11]. When applied to the mNET-seq data, eNRSA revealed significant overall increases in readthrough (Supplementary Fig. S1, *P* < 1.2e−12), indicating termination defects.

### eNRSA significantly increases speed and decreases memory usage

**Figure 5:**
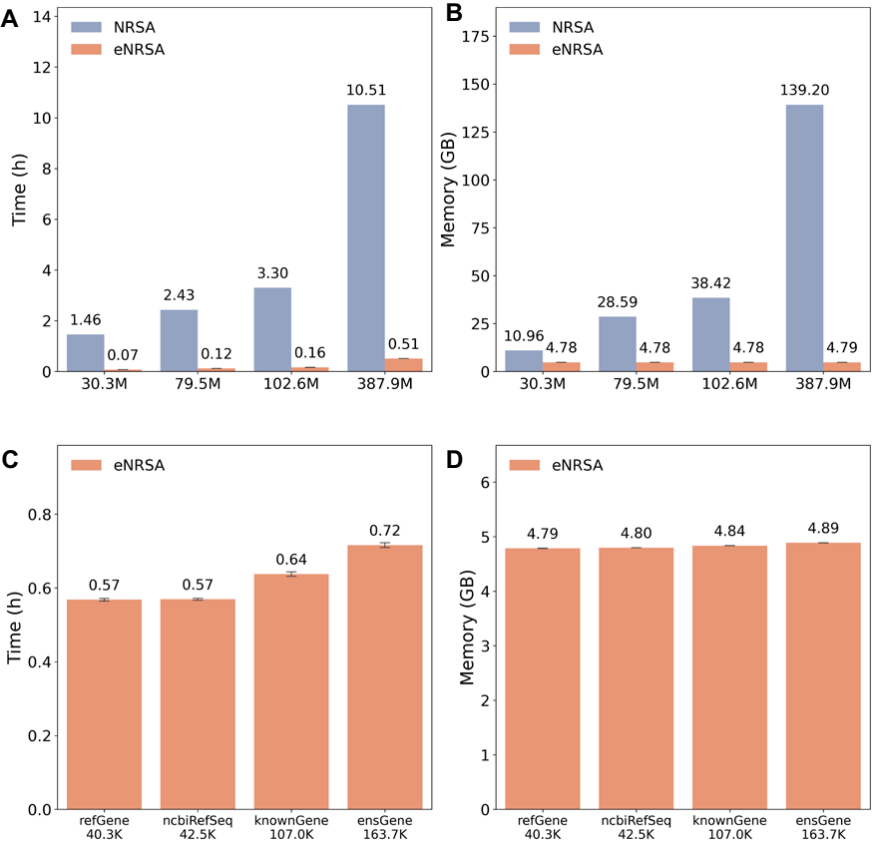
Performance comparison between eNRSA and NRSA. (A) Runtime with increasing numbers of PRO-seq reads. (B) Memory usage with increasing numbers of PRO-seq reads. (C) Runtime with increasing numbers of transcripts defined in the GTF file. (D) Memory usage with increasing numbers of transcripts defined in the GTF file. Computations were performed using a single thread of an Intel Xeon E5-2695 v4 @ 2.10 GHz processor with 1T memory.

Although NRSA has been widely used for nascent transcriptome data analysis [48–54], it struggles to handle large datasets efficiently, requiring long runtime and high memory usage. eNRSA improves both speed and memory efficiency by implementing Python in place of R, using novel algorithms, and adopting a streaming process. In simulations with increasing nascent transcriptome sizes, eNRSA reduced computational time by over 20-fold compared to NRSA. For example, NRSA took 1.46 hours to process 30.3 million nascent RNA reads, while eNRSA required only 0.07 hours. With 387.9 million reads, NRSA took 10.51 hours, whereas eNRSA completed the task in just 0.51 hours (Fig. 5A). Additionally, eNRSA significantly lowered memory demands. While NRSA’s memory usage scaled linearly with read counts (from 10.96 GB for 30.3 M reads to 139.2 GB for 387.9 M reads), eNRSA maintained a constant 4.78 GB memory requirement regardless of read counts (Fig. 5B). These results highlight eNRSA’s substantial computational efficiency over NRSA, making it a valuable tool for analyzing large datasets.

Since eNRSA automatically selects the major transcript for each gene from the gene structure file, we further evaluated its computational efficiency with respect to the number of transcripts defined in the GTF file. As a result, eNRSA showed only a minimal increase in runtime and consistent memory usage as the number of transcripts in the GTF file grew from 40.3 K (RefGene) to 163.7 K (EnsGene) (Fig. 5C, D).

## Discussion

eNRSA offers a powerful and scalable solution for nascent transcriptome analysis, addressing the limitations of NRSA while meeting the increasing demands of analyzing diverse organisms and complex study designs. By incorporating adaptive transcript selection, supporting multifactor designs, and introducing new capabilities to identify ATSSs, ATTSs, and transcription readthrough, eNRSA significantly enhances performance and broadens its applicability. Combined with substantial improvements in computational efficiency, eNRSA stands out as a scalable, efficient, and versatile tool for gaining deeper insights into transcriptional regulation across multiple stages. Although eNRSA is primarily designed to analyze GRO-seq or PRO-seq data, it can also be applied to data generated by other nascent RNA sequencing technologies that capture transcriptional dynamics, including pausing, elongation, and termination, such as Butt-seq, NET-seq, and mNET-seq. Examples of these applications are provided in Supplementary Table S6, Supplementary Fig. S1, and Supplementary Fig. S2, where the results are consistent with the findings reported in the original publications [11, 13, 55]. For nascent RNA sequencing technologies that capture only specific aspects of transcriptional dynamics, eNRSA results should be interpreted with caution. For instance, SLAM-seq tracks RNA kinetics, including transcription, processing, and degradation, and metabolically labeled RNAs are subject to degradation over time [12]. Therefore, while transcriptional changes within the gene body identified by eNRSA may still be meaningful, results related to pausing, ATSSs/ATTSs, and readthrough may not be interpretable—either because SLAM-seq does not capture pausing dynamics or due to RNA degradation, leading to limited coverage beyond transcription end sites. In contrast, TT-seq maps the entire range of transient RNA and monitors RNA synthesis and degradation, which does not yield peak signals near the promoter where polymerase pauses, such as GRO/PRO-seq, NET-seq, Butt-seq, or mNET-seq [14]. Therefore, eNRSA results on gene body, ATTSs, and readthrough may be informative, but those related to pausing may not be applicable.

To further advance the study of transcriptional regulation, eNRSA could be enhanced by integrating data from chromatin accessibility or binding assays, such as ATAC-seq or ChIP-seq. This integration would enable the linking of pausing behavior with chromatin state changes, transcription factor binding, and enhancer–promoter interactions, offering deeper insights into the regulatory landscape underlying transcription initiation, pausing, elongation, and termination. Additionally, eNRSA could be extended to analyze single-cell nascent transcriptome, such as scGRO-seq [56], unlocking new opportunities to study immediate, cell type–specific transcriptional changes and enabling a more detailed investigation of heterogeneity in gene expression across different cell types and conditions.

eNRSA relies heavily on predefined gene structures (e.g., from GTF files) to define gene boundaries for transcriptional quantification. While this approach is efficient, it may limit the tool’s ability to detect novel or poorly annotated genes, especially in regions with uncharacterized or alternative gene models. Implementing more flexible annotation systems or incorporating RNA-seq data could help address this limitation. eNRSA identifies ATSSs and ATTSs by detecting distinct promoter-proximal pausing and cleavage/polyadenylation signals. However, nascent transcription data have limited resolution when it comes to accurately defining alternative TSSs and TTSs, particularly in regions with overlapping or closely spaced initiation/termination sites. This limits eNRSA’s ability to fully capture all potential ATSS and ATTS events, especially in genes with complex promoter architectures or multiple isoforms. eNRSA uses read counts mapped to promoter-proximal and CPS regions to detect ATSSs and ATTSs, but transcriptional noise from neighboring genes can complicate the identification process. While eNRSA attempts to mitigate this noise by excluding closely located genes, this strategy may not always be sufficient, particularly when genes are densely packed or transcriptionally active in close proximity. Furthermore, eNRSA assumes that differences in promoter-proximal pausing regions suggest alternative TSS usage, but such differences can also arise from postinitiation regulatory mechanisms. To confidently link pausing behavior to TSS heterogeneity, it is essential to integrate complementary data types. For example, combining PRO-seq data with TSS-specific methods like CAGE-seq can help validate TSS locations. Additionally, RNA-seq data can assess differential isoform expression, which may align with alternative TSS usage. Mapping histone modifications, such as H3K4me3 (a mark of active promoters), can also provide insights into how pausing regions correlate with distinct TSSs.

## Methods

### Adaptive selection of major transcripts

Promoter-proximal and gene body regions are determined based on TSS and TTS sites. The promoter-proximal region is defined by examining each 50-bp window with a 5-bp sliding step along the coding strand, spanning ±500 bp from the TSS. The 50-bp window with the highest number of reads is selected as the promoter-proximal region. The gene body is defined as the region extending from +1 kb downstream of the TSS to the TTS [25].

Unlike NRSA, which preselects the longest transcript of each gene as the major transcript, eNRSA adopts a data-driven approach to determine the major transcript based on the nascent transcriptome data being analyzed. For each gene, eNRSA groups transcripts with identical TSS and TTS sites, quantifies transcription activity in the promoter-proximal and gene body regions for each group, and selects the transcript group with the highest reads in the promoter-proximal region as the major transcript. If 2 groups have the same number of promoter-proximal reads (i.e., share the same TSS), the transcript group with the highest number of reads in the gene body region is selected.

### Differential transcription analysis in complex study designs

After quantifying reads in the promoter-proximal and gene body regions, eNRSA estimates transcriptional alterations in both regions between 2 conditions. To facilitate differential expression analysis and accommodate complex study designs, eNRSA integrates PyDEseq2, a Python implementation of the DESeq2 workflow for differential expression analysis. Users provide an experimental design file that includes a column specifying the path to each sample’s alignment file and additional columns indicating each sample’s group assignments. Each sample can belong to multiple groups. eNRSA uses this design file to build a DESeq2 [57] model for differential comparisons.

To detect and quantify intergenic enhancers, eNRSA utilizes the HOMER package [58] to call novel transcripts using default parameters (tssFold >4 and bodyFold >3) on reads pooled from all samples. Transcripts located within −2 kb to +20 kb of any annotated gene are excluded. Active enhancers are then defined as regions exhibiting pairs of bidirectional transcripts, and their activity is quantified by the number of reads mapped to the corresponding enhancer region [25].

Notably, eNRSA allows users to provide their own normalization factors, such as those derived from spike-ins. If no normalization factor is provided, eNRSA applies the default DESeq2 normalization method to gene body expression and then uses the same normalization factor to normalize transcription in the promoter-proximal regions and enhancers.

### ATSS and ATTS identification

Nascent transcription sequencing captures promoter-proximal pausing, a regulatory step where RNA Pol II pauses after initiating transcription, typically 20–60 nucleotides downstream of the TSS. Distinct clusters of paused Pol II near a gene’s promoter, referred to as distinct pausing regions, may indicate alternative TSS usage. To identify potential ATSSs, eNRSA compares read counts within 2 promoter-proximal pausing regions under different conditions and evaluates the association between TSS usage and condition. For each gene, all TSS sites are considered, with pairwise comparisons restricted to TSSs separated by at least 1,000 bp to avoid mixed signals and enhance reliability. For example, if a gene has 3 TSSs (TSS1, TSS2, TSS3), eNRSA evaluates usage changes between TSS1 and TSS2, TSS1 and TSS3, and TSS2 and TSS3, while excluding closely spaced TSSs (<1,000 bp apart). Changes in TSS usage between conditions are assessed by constructing 2 × 2 contingency tables for each pair of samples, 1 from each condition, based on read counts corresponding to the 2 TSSs being compared. Rather than pooling data across samples, which may obscure biological variability, the CMH test is used to perform a stratified analysis. This test evaluates whether there is a consistent association between TSS usage and condition across sample pairs, while controlling for sample-specific variability. Under the assumption of a common odds ratio across strata, the CMH test increases statistical power and reduces bias due to heterogeneity among samples. This framework ensures robust detection of systematic shifts in TSS usage across biological replicates.

Similarly, nascent transcription accumulates at cleavage and polyadenylation sites, where distinct clusters of reads near a gene’s termination may indicate alternative TTS usage. To detect ATTSs, eNRSA compares read counts mapped between −1 kb and +2 kb of 2 TTS sites and assesses the association between TTS usage and the condition using a CMH test, analogous to the ATSS analysis. For each gene, all TTS sites were considered. To minimize transcriptional noise from neighboring genes, eNRSA excludes any TTS site that overlaps with another gene or has a gene located within 3 kb downstream.

### Readthrough dysregulation

Nascent transcription sequencing measures RNA still associated with actively transcribing RNA polymerase, enabling it to capture readthrough reads, which are transcripts extending beyond the normal termination site. eNRSA quantifies transcriptional readthrough using the ratio of the number of reads mapped within a fixed 50-kb region downstream of the TTS to the number of reads mapped within the last exon [44]. A significant change in this ratio under different conditions indicates readthrough dysregulation. To ensure reliability, eNRSA considers only active genes, defined as those with promoter-proximal read densities greater than zero and gene-body densities exceeding 4 reads per kilobase after total read counts are normalized to 10 million based on background estimation. The significance of changes in the readthrough ratio is assessed using the CMH test applied in a manner similar to the TSS analysis. To minimize transcriptional noise from neighboring genes, eNRSA excludes genes that have other active genes within 50 kb downstream.

### Running speed and memory improvements

To improve speed and reduce memory usage, eNRSA adopts an optimized strategy for transcriptional quantification. While NRSA uses a gene-centered approach that scans the nascent transcriptome data repeatedly to count reads mapped to each gene, eNRSA takes a more efficient approach. eNRSA begins by performing 2 types of read counting: one at the individual site-specific level and another by summing reads within 200-bp binned regions. These counts are then used to generate a comprehensive count matrix. The count matrix, created for each chromosome, is stored in a Python dictionary and saved as a binary pickle file for downstream analysis. For each gene defined in the GTF file, eNRSA consolidates transcripts with identical TSSs and TTSs into a single entity. The transcript group’s region is then overlapped with the precomputed count matrix for quantification. This strategy allows eNRSA to process the alignment data only once, significantly enhancing efficiency. Additionally, it groups the transcripts by chromosome, loading only the corresponding count matrix for the active chromosome. Once all transcripts for that chromosome are processed, the memory is released, further optimizing resource usage. By replacing all R-based coeds in NRSA with Python, eNRSA achieves faster runtimes, making it suitable for large-scale nascent transcriptome studies.

### eNRSA installation and implementation

eNRSA is implemented in Python 3 (≥3.6), and the dependencies include BEDTools, HOMER, and 2 Python packages—PyDESeq2 and Fisher. eNRSA can be executed in a standard Python environment with the required dependencies installed either by conda or Docker container. eNRSA, along with its detailed manual, including installation instructions, implementation guidelines, and output descriptions, is available at [26].

### Other bioinformatics analysis

To detect ATSSs and ATTSs from RNA-seq data, we calculated the ratio of reads mapped to the 2 alternative TSS or TTS sites for each sample pair, with 1 sample from each condition. To assess the significance of ATSS and ATTS events across sample pairs between the 2 conditions, we applied the CMH test.

The PCA plot, volcano plot, and the performance bar plots were generated in R using the ggplot2 package [59]. Functional enrichment analysis was performed by WebGestalt 2024 and GSEA_4.3.3 [34, 35]. The snapshots were from IGV_2.11.0 [60]. The PRO-seq profiles for readthrough disruption were generated by deepTools_3.5.6 with the last exon scaled to 1,000 bp [61].

### Nascent transcriptomic datasets

The PRO-seq data for DMSO-treated and dTAG47-treated cells in the G401 cell line are available at the Gene Expression Omnibus (GEO) under accession number GSE164926. The PRO-seq data for the G401 and Ramos cell lines can be accessed from GEO under accession numbers GSE173207 and GSE183781, respectively. RNA-seq data for the G401 and Ramos cell lines are available from GEO under accession numbers GSE173207 and GSE212456. The PRO-seq data for G401 cells expressing EGFP control and OmoMYC are available under accession number GSE109310. Additionally, the GRO-seq data from VEGF-A–stimulated and nonstimulated HUVEC (Human Umbilical Vein Endothelial Cell) cells, Butt-seq data from KL1-treated and DMSO-treated S2 cells, and S2P mNET-seq data from siCPSF73-treated, siCstF64+siCstF64t-treated, and control siRNA-treated HeLa cells are available at GSE52642, GSE228595 and GSE60358, respectively.

## Availability of Supporting Source Code and Requirements

Project name: eNRSA

Project homepage: https://bioinfo.vanderbilt.edu/eNRSA/

Operating system(s): Platform independent

Programming language: Python

Other requirements: Python3.8 or higher, HOMERs v5.1, bedtools v2.31.0

License: GNU GPL-3.0.

biotoolsID: biotools:enrsa; https://bio.tools/enrsa

RRID:SCR_026814

## Data Availability

A version of record snapshot of the GitHub repository has been archived in the Software Heritage [62].

## Additional Files

**Supplementary Fig. S1**. eNRSA was applied to S2P mNET-seq data from siCPSF73-treated, siCstF64+siCstF64t-treated, and control siRNA-treated HeLa cells (GSE60358). Significant readthrough increase was observed in siCPSF73-treated and siCstF64+siCstF64t-treated compared to siRNA control (**P < 1.2e−12, Mann–Whitney test). This result aligns with the findings in the original publication (Nojima et al., *Cell*, 2015).

**Supplementary Fig. S2**. eNRSA was applied to Butt-seq data from KL-1–treated and DMSO-treated S2 cells (GSE228595). An empirical cumulative distribution function (ECDF) plot of the pausing index revealed a significant increase upon KL-1 treatment (Kolmogorov–Smirnov test, *P* < 2.2 × 10^−16^), where the y-axis represents the cumulative fraction of genes, while the x-axis displays the log2-transformed pausing index. This result aligns with the findings in the original publication (Yu et al., *Genes Dev*, 2023).

**Supplementary Table S1**. Enriched gene sets in downregulated genes in dTAG47 vs. DMSO via ORA.

**Supplementary Table S2**. Enriched gene sets in downregulated genes in dTAG47 vs. DMSO via GSEA.

**Supplementary Table S3**. ATSSs between G401 and Ramos.

**Supplementary Table S4**. ATTSs between G401 and Ramos.

**Supplementary Table S5**. Readthrough dysregulation between OmoMYC and EGFP.

**Supplementary Table S6**. HUVEC_GRO-seq VEGF 2 h vs. Notx.

giaf071_Supplementary_Files

giaf071_Authors_Response_To_Reviewer_Comments_original_submission

giaf071_GIGA-D-25-00028_original_submission

giaf071_GIGA-D-25-00028_Revision_1

giaf071_Reviewer_1_Report_Original_submission Aniket Kumar -- 2/7/2025

giaf071_Reviewer_2_Report_Original_submission Mary Allen, Ph.D. -- 3/25/2025

## Abbreviations

ATSS: alternative transcription start site; ATTS: alternative transcription termination site; Butt-seq: bulk analysis of nascent transcript termini sequencing; caRNA-seq: chromatin-associated RNA sequencing; CMH: Cochran–Mantel–Haenszel test; CPS: cleavage and polyadenylation sites; EGFP: enhanced green fluorescent protein; eNRSA: enhanced NRSA; GEO: Gene Expression Omnibus; GRO-seq: global run-on sequencing; GTF: gene structure file; mNET-seq: mammalian NET-seq; NES: normalized enrichment score; NET-seq: native elongating transcript sequencing; NRSA: nascent RNA sequencing analysis; Pol II: RNA polymerase II; PRO-seq: precision run-on sequencing; SLAM-seq: thiol(SH)-linked alkylation for the metabolic sequencing of RNA; Start-seq: small capped RNA sequencing; TT-seq: transient transcriptome sequencing.
