# Supplementary material for: eNRSA: a faster and more powerful approach for nascent transcriptome analysis
Source: Gigascience. 2025 Jul 4;14:giaf071. doi: 10.1093/gigascience/giaf071 (PMC12231571; doi:10.1093/gigascience/giaf071)
Supplement: giaf071_Supplementary_Files [file giaf071_supplementary_files.zip › eNRSA-Suppl-Figures-revision.pdf]

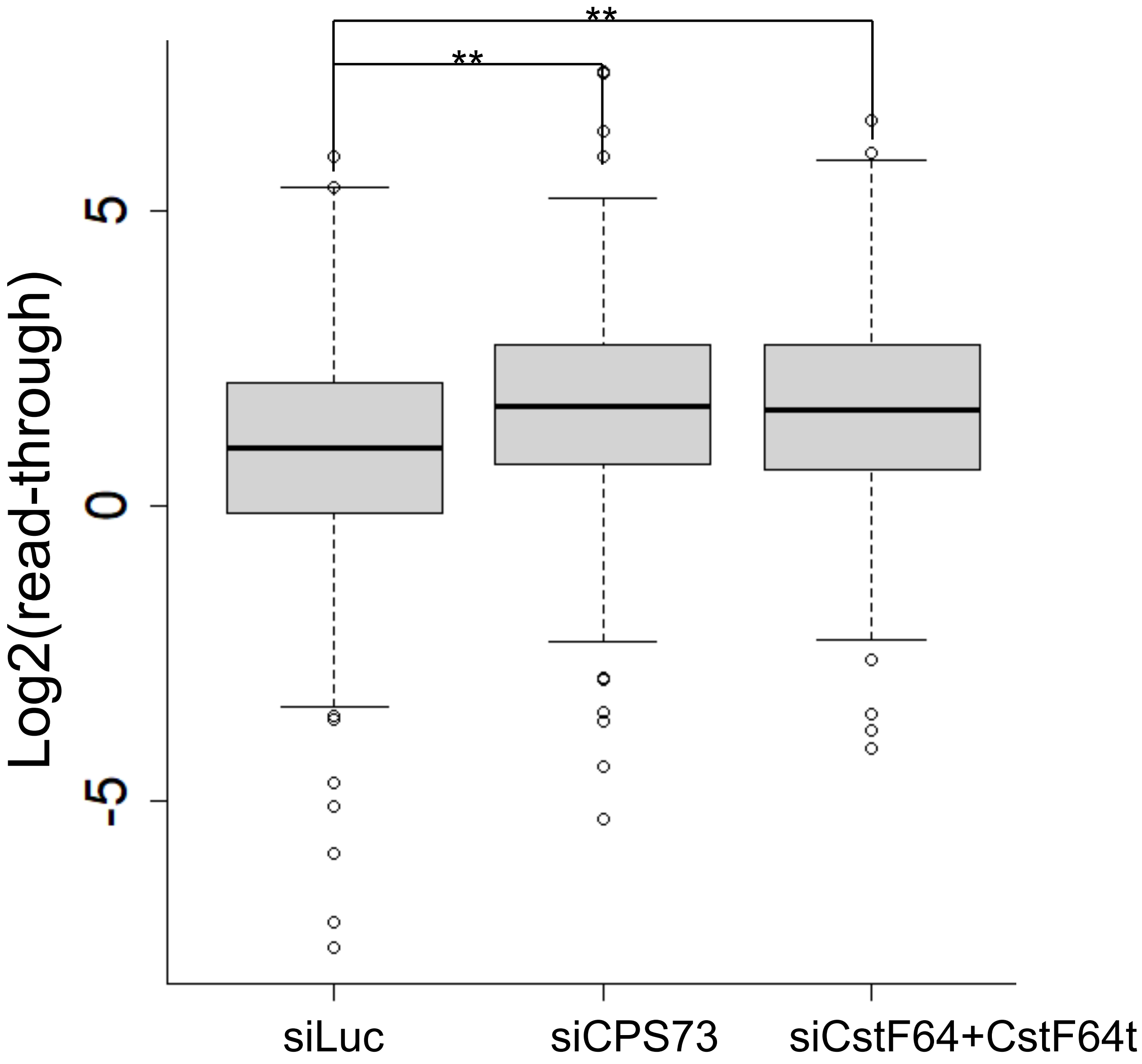

Supplementary Fig. S1. eNRSA was applied to S2P mNET-seq data from siCPSF73-treated, siCstF64+siCstF64t-treated, and control siRNA-treated HeLa cells (GSE60358). Significant readthrough increase was observed in siCPSF73-treated and siCstF64+siCstF64t-treated compared to siRNA control)(**: p< 1.2e−12, Mann-Whitney test). This result aligns with the findings in the original publication (Nojima, et al., Cell, 2015).

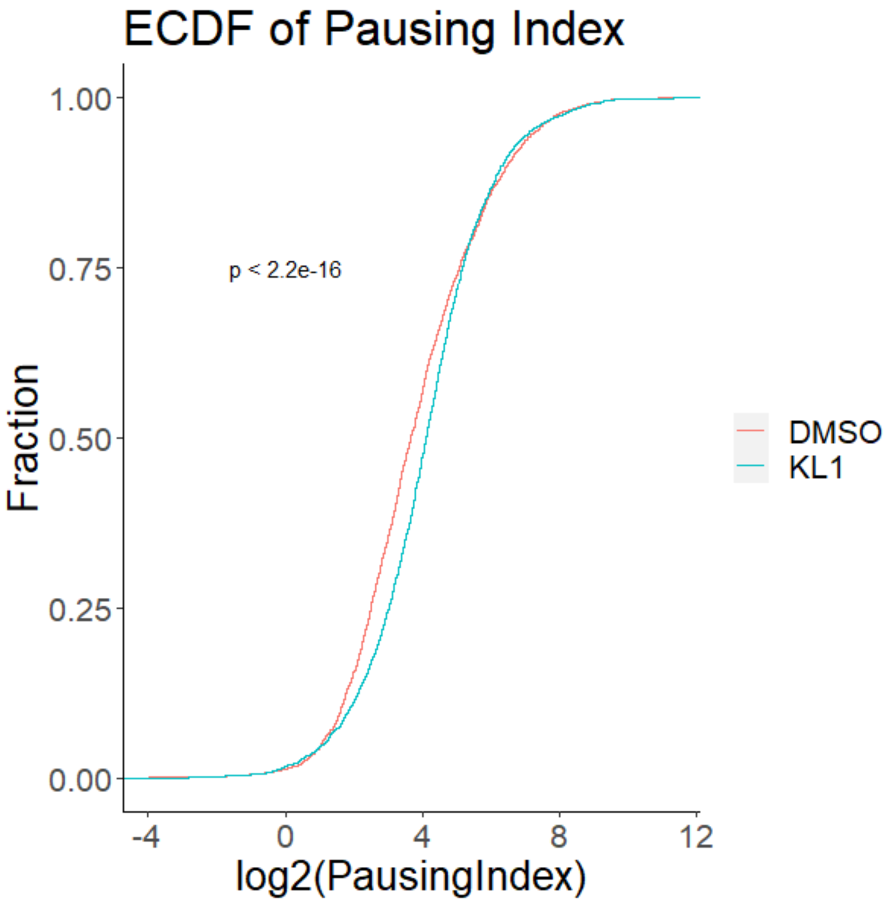

Supplementary Fig. S2. eNRSA was applied to Butt-seq data from KL-1 treated and DMSO treated S2 cells (GSE228595). An empirical cumulative distribution function (ECDF) plot of the pausing index revealed a significant increase upon KL-1 treatment (Kolmogorov–Smirnov test, $p < 2.2 \times 10^{-16}$), where Y-axis represents the cumulative fraction of genes, while the X-axis displays the log2-transformed pausing index. This result aligns with the findings in the original publication (Yu et al., Genes Dev, 2023).
