# Supplementary material for: eNRSA: a faster and more powerful approach for nascent transcriptome analysis
Source: Gigascience. 2025 Jul 4;14:giaf071. doi: 10.1093/gigascience/giaf071 (PMC12231571; doi:10.1093/gigascience/giaf071)
Supplement: giaf071_GIGA-D-25-00028_original_submission [file giaf071_giga-d-25-00028_original_submission.pdf]

GigaScience

eNRSA: A Faster and More Powerful Approach for Nascent Transcriptome Analysis

--Manuscript Draft--

**Manuscript Number:**	GIGA-D-25-00028
**Full Title:**	eNRSA: A Faster and More Powerful Approach for Nascent Transcriptome Analysis
**Article Type:**	Technical Note
**Funding Information:**	National Cancer Institute (CA200709, C247833) — Professor William P. Tansey<br>National Cancer Institute (CA229123, CA274367) — Professor Qi Liu<br>National Institutes of Health (AI139449) — Professor Qi Liu<br>Cancer Center Support Grant (CA068485) — Professor Qi Liu<br>Department of Biostatistics in VUMC (2024/2025 Biostatistics Faculty Development Award) — Dr. Jing Wang
**Abstract:**	Nascent RNA sequencing tracks primary transcriptional events, making it crucial for studying the immediate regulatory changes of genes and enhancers in response to both endogenous and exogenous stimuli. NRSA is a widely used tool for analyzing nascent transcriptomic data, enabling quantification of transcriptional changes at proximal promoters and gene bodies, estimation of pausing indices, identifying active enhancers, and establishing enhancer–target gene relationships. To improve its functionality and broaden its applicability to diverse organisms and complex study designs, we have developed an enhanced version, eNRSA. Key advancements include adaptive selection of major transcripts, support for any organism with known gene structures, compatibility with complex study designs, and identification of alternative transcription start and termination sites, as well as transcription readthrough events. Additionally, eNRSA achieves a ~20-fold increase in analysis speed while significantly reducing memory usage. These enhancements make eNRSA a faster, more versatile, and more powerful tool for nascent transcriptome analysis. eNRSA is freely available at https://bioinfo.vanderbilt.edu/eNRSA/.
**Corresponding Author:**	Qi Liu, Ph.D.<br>Vanderbilt University Medical Center<br>Nashville, TN UNITED STATES
**Corresponding Author Secondary Information:**	
**Corresponding Author's Institution:**	Vanderbilt University Medical Center
**Corresponding Author's Secondary Institution:**	
**First Author:**	Jing Wang
**First Author Secondary Information:**	
**Order of Authors:**	Jing Wang<br>Hua-chang Chen<br>Scott W. Hiebert<br>Quanhu Sheng<br>William P. Tansey<br>Yu Shyr<br>Qi Liu, Ph.D.
**Order of Authors Secondary Information:**	

Powered by Editorial Manager® and ProduXion Manager® from Aries Systems Corporation

Additional Information:	
**Question**	**Response**
Are you submitting this manuscript to a special series or article collection?	No
**Experimental design and statistics**<br><br>Full details of the experimental design and statistical methods used should be given in the Methods section, as detailed in our Minimum Standards Reporting Checklist. Information essential to interpreting the data presented should be made available in the figure legends.<br><br>Have you included all the information requested in your manuscript?	Yes
**Resources**<br><br>A description of all resources used, including antibodies, cell lines, animals and software tools, with enough information to allow them to be uniquely identified, should be included in the Methods section. Authors are strongly encouraged to cite Research Resource Identifiers (RRIDs) for antibodies, model organisms and tools, where possible.<br><br>Have you included the information requested as detailed in our Minimum Standards Reporting Checklist?	Yes
**Availability of data and materials**<br><br>All datasets and code on which the conclusions of the paper rely must be either included in your submission or deposited in publicly available repositories (where available and ethically appropriate), referencing such data using a unique identifier in the references and in the "Availability of Data and Materials" section of your manuscript.	Yes

Powered by Editorial Manager® and ProduXion Manager® from Aries Systems Corporation

Have you have met the above requirement as detailed in our Minimum Standards Reporting Checklist?	
GigaScience has policies and guidelines in place for the use of generative AI-writing tools such as ChatGPT. If you have used such writing tools to assist with writing the manuscript this must be declared and cited in the text. Authors should not list AI-writing tools and other AI-assisted technologies as an author or co-author and should acknowledge that they are fully responsible for text generated or refined by AI-writing tools.<p><br><br>A summary of use (particularly in the introduction or among methods) needs to be included at the end of the paper, and the outputs should also be included as a supplementary file hosted in GigaDB or other open repositories. Please read our guidelines for more information. <p><br><br>By submitting to GigaScience, you are aware of the journal's AI-writing tools policy, and if you have declared use of such tools below, you have acknowledged this where appropriate in your manuscript and have made a summary of use and outputs available. <p><br><b>AI-assisted writing tools have been used in the preparation of this manuscript?	No

Manuscript Click here to access/download;Manuscript;eNRSA-manuscript.docx

eNRSA: A Faster and More Powerful Approach for Nascent Transcriptome Analysis

Jing Wang [1,2, †], Hua-chang Chen [1,2, †], Scott W. Hiebert [3,4], Quanhu Sheng [1,2], William P. Tansey [3,5], Yu Shyr [1,2, *], Qi Liu[1,2, *]

[1]Department of Biostatistics, Vanderbilt University School of Medicine, Nashville, TN, USA

[2]Center for Quantitative Sciences, Vanderbilt University Medical Center, Nashville, TN, USA

[3]Department of Biochemistry, Vanderbilt University School of Medicine, Nashville, TN, USA.

[4]Vanderbilt-Ingram Cancer Center, Nashville, TN, USA.

[5] Department of Cell and Developmental Biology, Vanderbilt University School of Medicine, Nashville, TN, United States

† These authors contributed equally to this work

*** Correspondence**
Yu Shyr, yu.shyr@vumc.org; Qi Liu, qi.liu@vumc.org

Keywords: nascent transcriptome analysis, adaptive major transcript, alternative transcription start site (ATSS), alternative transcription termination site (ATTS), transcription readthrough

Abstract

Nascent RNA sequencing tracks primary transcriptional events, making it crucial for studying the immediate regulatory changes of genes and enhancers in response to both endogenous and exogenous stimuli. NRSA is a widely used tool for analyzing nascent transcriptomic data, enabling quantification of transcriptional changes at proximal promoters and gene bodies, estimation of pausing indices, identifying active enhancers, and establishing enhancer–target gene relationships. To improve its functionality and broaden its applicability to diverse organisms and complex study designs, we have developed an enhanced version, eNRSA. Key advancements include adaptive selection of major transcripts, support for any organism with known gene structures, compatibility with complex study designs, and identification of alternative transcription start and termination sites, as well as transcription readthrough events. Additionally, eNRSA achieves a ~20-fold increase in analysis speed while significantly reducing memory usage. These enhancements make eNRSA a faster, more versatile, and more powerful tool for

nascent transcriptome analysis. eNRSA is freely available at https://bioinfo.vanderbilt.edu/eNRSA/.

Introduction

Transcription is a highly regulated process comprising multiple stages, each precisely controlled to ensure accurate gene expression [1-4]. These key transcriptional stages include initiation, pausing, elongation, and termination [4]. Unlike steady-state RNA sequencing, nascent RNA sequencing captures transcription that are actively synthesized, providing a direct measure of gene expression across various regulatory stages [5, 6]. This capability is particularly valuable for uncovering immediate, direct, and transient transcriptional changes that reflect cellular responses to diverse conditions, including stress, differentiation, or disease progression.

There are several sequencing-based techniques designed to extract newly transcribed RNAs from the total pool of cellular RNA. These include small capped RNA sequencing (Start-seq) [7], chromatin-associated RNA sequencing (caRNA-seq) [8, 9], global run-on sequencing (GRO-seq) [5], precision run-on sequencing (PRO-seq) [6], native elongating transcript sequencing (NET-seq) [10], mammalian NET-seq (mNET-seq) [11], thiol(SH)-linked alkylation for the metabolic sequencing of RNA (SLAM-seq) [12], bulk analysis of nascent transcript termini sequencing (Butt-seq) [13], and transient transcriptome sequencing (TT-seq) [14]. Of these, GRO-seq and PRO-seq are among the most widely used methods, largely due to their ability to provide high-resolution, genome-wide data on actively transcribing RNA polymerases. To analyze GRO/PRO-seq data, several tools have been developed, including dREG, FStitch, groHMM, Vespucci, nASAP, and NRSA (Fig. 1). dREG [15, 16] and FStitch [17] focus on identifying active regulatory elements such as enhancers with divergent transcription. groHMM [18] quantifies nascent transcription for both known genes and enhancers, while Vespucci [19] also estimates transcriptional changes between conditions. nASAP [20] is a web server for nascent RNA analysis including transcription level quantification, pausing site identification and regulatory network construction. Among these tools, NRSA [21] stands out for its comprehensive analysis of nascent transcription. It not only quantifies nascent transcription and pausing for known genes, but also detects, annotates, quantifies, and prioritizes active enhancers (Fig. 1). However, NRSA is time- and memory-intensive, particularly when processing large-scale nascent transcriptomes. Additionally, its limitation to single-factor designs and the requirement to preprocess gene structure files by selecting one major transcript per gene diminishes its performance and restricts its applicability across diverse organisms and genomes.

Here, we developed eNRSA (enhanced NRSA) to significantly improve computational efficiency, broaden its applicability, and enhance performance (Fig. 1). eNRSA runs 20 times faster than NRSA while requiring only roughly 1/8 of the memory. Further, eNRSA

enhances performance by adaptively selecting major transcripts based on the nascent transcriptome being analyzed and by supporting multi-factor experimental designs. These advancements make eNRSA applicable to any organism or genome with a known gene structure. By fully leveraging the unique characteristics of the nascent transcriptome, eNRSA introduces new functionalities to identify alternative transcription start sites (ATSS), alternative transcription termination sites (ATTS), and readthrough dysregulation across conditions. eNRSA is freely available at https://bioinfo.vanderbilt.edu/eNRSA/.

Results

Overview of eNRSA

Nascent RNA sequencing captures the production of newly synthesized RNAs, offering a comprehensive view of regulatory dynamics throughout the transcription cycle, including initiation, pausing, elongation, and termination [4, 22]. In 2018, we developed NRSA, which enables in-depth analysis of the nascent transcriptome at both gene and enhancer levels, surpassing other tools in scope and accuracy [21]. NRSA not only estimates promoter-proximal pausing and elongation rates, but also identifies, annotates, and quantifies active enhancers while measuring enhancer-mediated regulation.

To enhance NRSA's performance and broaden its applications, we developed an advanced version, eNRSA, which supports any organism with a known gene structure, accommodates complex study designs, introduces new functions to identify ATSS, ATTS, and readthrough dysregulation, and significantly improves computational efficiency (Fig. 1). eNRSA takes nascent transcriptome data and a reference genome with a gene structure file as input, providing detailed outputs on transcriptional changes in promoter-proximal and gene body regions, alternative transcriptional events (including ATSS, ATTS, and readthrough), enhancer activity, and the pausing index, along with various visualization options (Fig. 2).

eNRSA introduces three key advancements: 1) adaptive selection of major transcripts, enabling data-driven definition of promoter-proximal and gene body regions, extending support to any organism with defined gene structures (compared to NRSA's limitation of five organisms and eight genomes); 2) advanced capabilities for complex study designs, improving differential transcriptional analysis by accounting for confounding factors and allowing for multifactorial designs; and 3) new functions to identify ATSS, ATTS, and readthrough dysregulation (Fig. 2). Additionally, eNRSA significantly optimizes computational performance by simultaneously increasing processing speed and reducing memory requirements.

eNRSA selects major transcripts adaptively and supports any organism with a known gene structure

One primary function of NRSA is to quantify transcriptional changes at promoter-proximal and gene body regions and to estimate the pausing index, which heavily depends on selecting the major transcript for each gene. Given that a single gene often encodes multiple transcripts, selecting the major transcript is crucial for accurately representing the gene's nascent transcription. This selection significantly impacts the definition of the corresponding promoter-proximal and gene body regions, ultimately influencing the accuracy of transcriptional regulation quantification. To simplify calculations, NRSA uses the longest transcript for each gene as the major transcript, defining promoter-proximal and gene body regions accordingly. Although NRSA preprocesses the gene structure file (GTF file) to extract the longest transcript for each gene from eight genomes across five organisms (hg19, hg38, mm10, mm39, dm3, dm6, ce10, and danRer10) and packs these preprocessed files in the tool for seamless analysis, users have to perform this preprocessing themselves if working with other organisms or different genome versions. This task is non-trivial and demands programming skills, limiting NRSA's broad application to various organisms. Furthermore, this approach may yield misleading findings if the longest transcript is not actually the major transcript for a given gene.

To address these issues, eNRSA automatically and adaptively selects major transcripts based on the nascent transcriptome data being analyzed. Unlike NRSA, which relies on preselected longest transcripts, eNRSA identifies transcripts with the highest nascent transcriptional levels as major transcripts. This data-driven approach is more accurate and flexible than a fixed selection method, as major transcripts may vary under different conditions. By removing the preprocessing step, eNRSA supports any organism with a known gene structure without additional procedures.

eNRSA improves differential transcription analysis by allowing complex study designs

Most nascent transcriptome analysis tools, including NRSA, are limited to handling simple study designs involving only two groups separated by a single factor of interest [19, 21], lacking the ability to analyze complex study designs with multiple factors or confounding variables, such as batch effects. This limitation restricts NRSA's applicability and can result in unreliable and irreproducible findings. Failing to account for confounding factors can lead to either false differential transcription arising from technical rather than biological effects or loss of true differential signals.

For example, when NRSA was used to compare the nascent transcriptome between two conditions—DMSO-treated and dTAG47-treated cells for MYC binding depletion [23], it identified only 5 upregulated and 62 downregulated genes with an FDR<0.05, with no notable pathways emerging from functional enrichment analysis. Principal component analysis, however, revealed a strong batch effect, with DMSO-treated and dTAG47-treated cells from the same batch clustering even more closely than cells from the same

condition in different batches (Fig. 3A). The lack of batch-effect correction likely explains why NRSA did not yield biologically meaningful results.

In contrast, eNRSA supports the analysis of complex study designs, with the ability to adjust for confounding factors, thereby enhancing differential transcription analysis. When applied to the same dataset of DMSO-treated and dTAG47-treated with the batch factor incorporated in the model, eNRSA identified 1,269 dysregulated genes with an FDR<0.05, including 440 upregulated and 829 downregulated ones (Fig. 3B). Notably, most downregulated genes in dTAG47-treated cells were either MYC targets or associated with core MYC functions. MYC is known to control the transcription of genes essential for ribosome biogenesis [24-28]. Consistently, eNRSA detected significant downregulation of ribosome-related genes in dTAG47-treated cells, including *RPLP2*, *RPL12*, *RPL13*, *RPL14*, *RPL32*, *RPL34*, *RPL35*, and *RPS24* (Fig. 3C; Supplementary Table 1). Functional enrichment analysis using WebGestalt [29] and GSEA [30] revealed significant downregulation of genes involved in ribosome biogenesis, mTORC1 signaling, and MYC targets (Normalized Enrichment Score (NES)= -3.07, FDR=0), as well as rRNA processing (NES=-3.05, FDR=0) (Figs. 3D and 3E; Supplementary Table 2). These findings demonstrate that eNRSA effectively unmasks true differential transcription signals previously obscured by batch effects, significantly enhancing detection power.

eNRSA identifies ATSS, ATTS and readthrough dysregulation

In mammalian genomes, most genes give rise to multiple transcript isoforms. At least 70% of genes have multiple polyadenylation sites, more than 50% have alternative transcription start sites, and transcripts from nearly all genes can be subject to alternative splicing [31-34]. Differential transcript isoforms can encode products that differ in structure, location, stability, enzyme activity, and other properties, which regulate key biological processes and contribute to disease. Therefore, identifying these alternative events is crucial [31].

ATSS and ATTS have been reported to contribute more to isoform diversity than alternative splicing [31]. Nascent transcriptome data from GRO/PRO-seq, (m)NET-seq, and Butt-seq, are characterized by peaks at promoter-proximal regions and an accumulation of reads at cleavage and polyadenylation sites (CPS), providing a natural way to identify ATSS and ATTS. However, no tools are currently available to specifically identify ATSS and ATTS from nascent transcriptome data. Leveraging these unique characteristics, eNRSA identifies ATSS by detecting shifts in read distributions across promoter-proximal regions, and ATTS by detecting shifts across CPS regions, between two conditions. Each promoter-proximal region corresponds to a TSS site, where higher read counts indicate greater TSS usage. A shift in read enrichment from one TSS site to another signals an ATSS event, which eNRSA assesses using the Cochran–Mantel–Haenszel test (CMH) for statistical significance (details in Methods). Similarly, eNRSA

detects ATTS events by counting and estimating shifts in read distributions across CPS regions.

eNRSA demonstrated strong performance in detecting ATSS and ATTS events, with findings further supported by RNA-seq data. When applied to compare nascent transcriptome between two cell lines, Ramos and G401, eNRSA identified 1,600 ATSSs and 1,400 ATTSs at an FDR < 0.05 (Supplementary Tables 3 & 4). Among these, the *SCP2* gene exhibited one of the most significant ATSS event (FDR=0). In the G401 cell line, promoter-proximal peaks were observed at two TSS sites: one at chr1:53,392,901 (hg19) corresponding to the long transcript NM_001193599.2, and the other at chr1:53,480,610 (hg19) corresponding to the short transcript NM_001007250.2. In contrast, only a single peak was observed at the second TSS site (chr1:53,480,610) in the Ramos cell line (Fig. 4A). This shift in read enrichment—from two TSS sites in G401 to one in Ramos—clearly indicates an ATSS event. This event was validated by RNA-seq data from the same cell lines, where both long and short transcripts were detected in G401, but only the short transcript was present in Ramos (Fig. 4A). The differential TSS usage between these cell lines suggests that *SCP2* may produce distinct proteins with differing functions. According to Uniprot, the two *SCP2* transcripts encode distinct proteins through transcription initiation from independently regulated promoters. The long transcript encodes SCPx, a thiolase enzyme essential for peroxisomal oxidation of branched-chain fatty acids [35], while the short transcript encodes SCP2, an intracellular lipid transfer protein that facilitates the transfer of common phospholipids, cholesterol, and gangliosides from the endoplasmic reticulum to the plasma membrane [36-38].

In addition to ATSS and ATTS, nascent transcriptome profiles provide direct measurements of transcription readthrough, where transcription continues beyond normal termination sites [39-45]. Transcription readthrough is observed not only in various cellular stress conditions but also in healthy tissues, suggesting that readthrough transcripts may play a role in regulating cellular processes [45][46]. To detect readthrough dysregulation, eNRSA calculates a readthrough ratio by counting reads 50 kb downstream of the TTS relative to reads in the terminal exon [47], then estimates the ratio change between two conditions to identify dysregulations (details in Methods). Using this approach, eNRSA identified 217 protein-coding genes with reduced readthrough, and 160 with increased readthrough, in engineered G401 cells expressed OmoMYC—a dominant-negative mutant that blocks the productive association of MYC with its target genes, compared to cells expressing inducible forms of enhanced green fluorescent protein (EGFP, control) (FDR < 0.05; Supplementary Table 5). Metagene analysis of nascent transcription for genes with increased readthrough in OmoMYC G401 cells revealed consistent read accumulation beyond the TTS, whereas genes with decreased readthrough showed read depletion in this region (Fig. 4B).

eNRSA significantly increases speed and decreases memory usage

Although NRSA has been widely used for nascent transcriptome data analysis [48-54], it struggles to handle large datasets efficiently, requiring long runtime and high memory usage. eNRSA improves both speed and memory efficiency by implementing Python in place of R, using novel algorithms, and adopting a streaming process. In simulations with increasing nascent transcriptome sizes, eNRSA reduced computational time by over 20-fold compared to NRSA. For example, NRSA took 1.46 hours to process 30.3 million nascent RNA reads, while eNRSA required only 0.07 hours. With 387.9 million reads, NRSA took 10.51 hours, whereas eNRSA completed the task in just 0.51 hours (Fig. 5A). Additionally, eNRSA significantly lowered memory demands. While NRSA's memory usage scaled linearly with read counts (from 10.96 GB for 30.3M reads to 139.2 GB for 387.9M reads), eNRSA maintained a constant 4.78 GB memory requirement regardless of read counts (Fig. 5B). These results highlight eNRSA's substantial computational efficiency over NRSA, making it a valuable tool for analyzing large datasets.

Since eNRSA automatically selects the major transcript for each gene from the gene structure file, we further evaluated its computational efficiency with respect to the number of transcripts defined in the GTF file. As a result, eNRSA showed only a minimal increase in runtime and consistent memory usage as the number of transcripts in the GTF file grew from 40.3K (RefGene) to 163.7K (EnsGene) (Figs. 5C & 5D).

Discussion

eNRSA offers a powerful and scalable solution for nascent transcriptome analysis, addressing the limitations of NRSA while meeting the increasing demands of analyzing diverse organisms and complex study designs. By incorporating adaptive transcript selection, supporting multifactor designs, and introducing new capabilities to identify ATSS, ATTS, and transcription readthrough, eNRSA significantly enhances performance and broadens its applicability. Combined with substantial improvements in computational efficiency, eNRSA stands out as a scalable, efficient, and versatile tool for gaining deeper insights into transcriptional regulation across multiple stages. Although eNRSA is primarily designed to analyze GRO-seq or PRO-seq data, it can also be applied to data generated by other nascent RNA sequencing technologies that capture transcriptional pausing and elongation, such as Butt-seq, NET-seq, and mNET-seq.

To further advance the study of transcriptional regulation, eNRSA could be enhanced by integrating data from chromatin accessibility or binding assays, such as ATAC-seq or ChIP-seq. This integration would enable the linking of pausing behavior with chromatin state changes, transcription factor binding, and enhancer-promoter interactions, offering deeper insights into the regulatory landscape underlying transcription initiation, pausing, elongation, and termination. Additionally, eNRSA could be extended to analyze single-cell nascent transcriptome, such as scGRO-seq [55], unlocking new opportunities to study

immediate, cell-type-specific transcriptional changes and enabling a more detailed investigation of heterogeneity in gene expression across different cell types and conditions.

eNRSA relies heavily on predefined gene structures (e.g., from GTF files) to define gene boundaries for transcriptional quantification. While this approach is efficient, it may limit the tool's ability to detect novel or poorly annotated genes, especially in regions with uncharacterized or alternative gene models. Implementing more flexible annotation systems or incorporating RNA-Seq data could help address this limitation. eNRSA identifies ATSS and ATTS by detecting distinct promoter-proximal pausing and cleavage/polyadenylation signals. However, nascent transcription data has limited resolution when it comes to accurately defining alternative TSS and TTS, particularly in regions with overlapping or closely spaced initiation/termination sites. This limits eNRSA's ability to fully capture all potential ATSS and ATTS events, especially in genes with complex promoter architectures or multiple isoforms. eNRSA uses read counts mapped to promoter-proximal and CPS regions to detect ATSS and ATTS, but transcriptional noise from neighboring genes can complicate the identification process. While eNRSA attempts to mitigate this noise by excluding closely located genes, this strategy may not always be sufficient, particularly when genes are densely packed or transcriptionally active in close proximity. Furthermore, eNRSA assumes that differences in promoter-proximal pausing regions suggest alternative TSS usage, but such differences can also arise from post-initiation regulatory mechanisms. To confidently link pausing behavior to TSS heterogeneity, it is essential to integrate complementary data types. For example, combining PRO-seq data with TSS-specific methods like CAGE-seq can help validate TSS locations. Additionally, RNA-seq data can assess differential isoform expression, which may align with alternative TSS usage. Mapping histone modifications, such as H3K4me3 (a mark of active promoters), can also provide insights into how pausing regions correlate with distinct TSSs.

Methods

Adaptive selection of major transcripts

Promoter-proximal and gene body regions are determined based on TSS and TTS sites. The promoter-proximal region is defined by examining each 50 bp window with a 5 bp sliding step along the coding strand, spanning ±500 bp from the TSS. The 50 bp window with the highest number of reads is selected as the promoter-proximal region. The gene body is defined as the region extending from +1 kb downstream of the TSS to the TTS [21].

Unlike NRSA, which preselects the longest transcript of each gene as the major transcript, eNRSA adopts a data-driven approach to determine the major transcript based on the nascent transcriptome data being analyzed. For each gene, eNRSA groups transcripts with identical TSS and TTS sites, quantifies transcription activity in the promoter-proximal and gene body regions for each group, and selects the transcript group with the highest reads in the promoter-proximal region as the major transcript. If two groups have the same number of promoter-proximal reads (i.e., share the same TSS), the transcript group with the highest number of reads in the gene body region is selected.

Differential transcription analysis in complex study designs

After quantifying reads in the promoter-proximal and gene body regions, eNRSA estimates transcriptional alterations in both regions between two conditions. To facilitate differential expression analysis and accommodate complex study designs, eNRSA integrates PyDEseq2, a Python implementation of the DESeq2 workflow for differential expression analysis. Users provide an experimental design file that includes a column specifying the path to each sample's alignment file, and additional columns indicating each sample's group assignments. Each sample can belong to multiple groups. eNRSA uses this design file to build a DESeq2 [56] model for differential comparisons.

Notably, eNRSA allows users to provide their own normalization factors, such as those derived from spike-ins. If no normalization factor is provided, eNRSA applies the default DESeq2 normalization method to gene body expression and then uses the same normalization factor to normalize transcription in the promoter-proximal regions.

ATSS and ATTS identification

Nascent transcription sequencing captures promoter-proximal pausing, a regulatory step where RNA polymerase II (Pol II) pauses after initiating transcription, typically 20–60 nucleotides downstream of the TSS. Distinct clusters of paused Pol II near a gene's promoter, referred to as distinct pausing regions, may indicate alternative TSS usage. To identify potential ATSS, eNRSA compares read counts within two promoter-proximal pausing regions under different conditions and evaluates the association between TSS usage and the condition using the CMH test. To enhance reliability, eNRSA excludes closely spaced TSSs (<1000 bp).

Similarly, nascent transcription accumulates at cleavage and polyadenylation sites, where distinct clusters of reads near a gene's termination may indicate alternative TTS usage. To detect ATTS, eNRSA compares read counts mapped between -1 kb and +2 kb of two TTS sites and assesses the association between TTS usage and the condition using the CMH test. To reduce transcriptional noise from neighboring genes, eNRSA excludes a TTS site if it overlaps with any gene or if there is a gene within 3 kb downstream.

Readthrough dysregulation

Nascent transcription sequencing measures RNA still associated with actively transcribing RNA polymerase, enabling it to capture readthrough reads, which are transcripts extending beyond the normal termination site. eNRSA quantifies transcriptional readthrough using the ratio of reads mapped within 50 kb downstream of the TTS to reads mapped to the last exon (Fig. S1C) [47]. A significant change in this ratio under different conditions indicates readthrough dysregulation. To ensure reliability, eNRSA considers only active genes, defined as those with promoter-proximal read densities greater than zero and gene-body densities exceeding four reads per kilobase after total read counts are normalized to 10 million based on background estimation. The significance of changes in the readthrough ratio is assessed using the CMH test. To minimize transcriptional noise from neighboring genes, eNRSA excludes genes that have other active genes within 50 kb downstream.

Running speed and memory improvements

To improve speed and reduce memory usage, eNRSA adopts an optimized strategy for transcriptional quantification. While NRSA uses a gene-centered approach that scans the nascent transcriptome data repeatedly to count reads mapped to each gene, eNRSA takes a more efficient approach. eNRSA begins by performing two types of read counting: one at the individual site-specific level and another by summing reads within 200 bp binned regions. These counts are then used to generate a comprehensive count matrix. The count matrix, created for each chromosome, is stored in a python dictionary and saved as a binary pickle file for downstream analysis. For each gene defined in the GTF file, eNRSA consolidates transcripts with identical TSS and TTS into a single entity. The transcript group's region are then overlapped with the precomputed count matrix for quantification. This strategy allows eNRSA to process the alignment data only once, significantly enhancing efficiency. Additionally, it groups the transcripts by chromosome, loading only the corresponding count matrix for the active chromosome. Once all transcripts for that chromosome are processed, the memory is released, further optimizing resource usage. By replacing all R-based coeds in NRSA with Python, eNRSA achieves faster runtimes, making it suitable for large-scale nascent transcriptome studies.

eNRSA installation and implementation

eNRSA is implemented in Python 3 (> = 3.6) and the dependencies include BEDTools, HOMER, and two Python packages - PyDESeq2 and Fisher. eNRSA can be executed in a standard Python environment with the required dependencies installed either by conda or Docker container. A Docker image can be found at Docker Hub (https://hub.docker.com/repository/docker/chccode/enrsa/). eNRSA, along with its detailed manual, including installation instructions, implementation guidelines, and output descriptions, are available at https://bioinfo.vanderbilt.edu/eNRSA/.

Other bioinformatics analysis

The PCA plot, volcano plot and the performance bar plots were generated in R using ggplot2 package [57]. Functional enrichment analysis was performed by WebGestalt 2024 and GSEA_4.3.3 [29, 30]. The snapshots were from IGV_2.11.0 [58]. The PRO-seq profiles for readthrough disruption were generated by deepTools_3.5.6 with the last exon scaled to 1000bp [59].

Nascent transcriptomic datasets

The PRO-seq data for DMSO-treated and dTAG47-treated cells in the G401 cell line are available at the Gene Expression Omnibus (GEO) under accession number GSE164926. The PRO-seq data for the G401 and Ramos cell lines can be accessed from GEO under accession numbers GSE173207 and GSE183781, respectively. RNA-seq data for the G401 and Ramos cell lines are available from GEO under accession numbers GSE173207 and GSE212456. Additionally, the PRO-seq data for G401 cells expressing EGFP control and OmoMYC are available under accession number GSE109310.

Availability of supporting source code and requirements

Project name: eNRSA

Project home page: https://bioinfo.vanderbilt.edu/eNRSA/

Operating system(s): Platform independent

Programming language: Python

Other requirements: Python3.8 or higher, HOMERs v5.1, bedtools v2.31.0

License: GNU GPL-3.0.

List of abbreviations

ATSS: alternative transcription start sites

ATTS: alternative transcription termination sites

Butt-seq: bulk analysis of nascent transcript termini sequencing

caRNA-seq: chromatin-associated RNA sequencing

CMH: Cochran–Mantel–Haenszel test

CPS: cleavage and polyadenylation sites

EGFP: enhanced green fluorescent protein

eNRSA: enhanced NRSA

GEO: Gene Expression Omnibus

GRO-seq: global run-on sequencing

GTF: gene structure file

mNET-seq: mammalian NET-seq

NES: normalized enrichment score

NET-seq: native elongating transcript sequencing

NRSA: nascent RNA sequencing analysis

Pol II: RNA polymerase II

PRO-seq: precision run-on sequencing

SLAM-seq: thiol(SH)-linked alkylation for the metabolic sequencing of RNA

Start-seq: small capped RNA sequencing

TT-seq: transient transcriptome sequencing

Competing interests

The authors declare that they have no competing interests.

Funding

This work is supported by National Cancer Institute grants (P01CA229123, U54 CA274367, R01 CA200709, and R01 CA247833), National Institutes of Health (P01 AI139449), Cancer Center Support Grant (P30CA068485), 2024/2025 Biostatistics Faculty Development Award from Department of Biostatistics in VUMC.

Authors' contributions

Jing Wang: Data curation, Conceptualization, Formal analysis, Methodology, Supervision, Writing – original draft, Writing – review & editing, Funding acquisition. Hua-chang Chen: Writing – original draft, Software, Resources, Methodology, Formal analysis, Data curation. Scott W. Hibert: Writing – review & editing, Investigation. Quanhu Sheng: Software, Writing – review & editing. William P. Tansey: Writing – review & editing, Investigation, Funding acquisition. Yu Shyr: Writing – review & editing, Investigation. Qi

Liu: Writing – review & editing, Supervision, Methodology, Investigation, Project administration, Funding acquisition, Conceptualization.

References

1. Tian, B. and J.H. Graber, *Signals for pre-mRNA cleavage and polyadenylation.* Wiley Interdiscip Rev RNA, 2012. **3**(3): p. 385-96.
2. Fuda, N.J., M.B. Ardehali, and J.T. Lis, *Defining mechanisms that regulate RNA polymerase II transcription in vivo.* Nature, 2009. **461**(7261): p. 186-92.
3. Vihervaara, A., F.M. Duarte, and J.T. Lis, *Molecular mechanisms driving transcriptional stress responses.* Nat Rev Genet, 2018. **19**(6): p. 385-397.
4. Wissink, E.M., et al., *Nascent RNA analyses: tracking transcription and its regulation.* Nat Rev Genet, 2019. **20**(12): p. 705-723.
5. Core, L.J., J.J. Waterfall, and J.T. Lis, *Nascent RNA sequencing reveals widespread pausing and divergent initiation at human promoters.* Science, 2008. **322**(5909): p. 1845-8.
6. Kwak, H., et al., *Precise maps of RNA polymerase reveal how promoters direct initiation and pausing.* Science, 2013. **339**(6122): p. 950-3.
7. Nechaev, S., et al., *Global analysis of short RNAs reveals widespread promoter-proximal stalling and arrest of Pol II in Drosophila.* Science, 2010. **327**(5963): p. 335-8.
8. Bhatt, D.M., et al., *Transcript dynamics of proinflammatory genes revealed by sequence analysis of subcellular RNA fractions.* Cell, 2012. **150**(2): p. 279-90.
9. Pandya-Jones, A., et al., *Splicing kinetics and transcript release from the chromatin compartment limit the rate of Lipid A-induced gene expression.* RNA, 2013. **19**(6): p. 811-27.
10. Churchman, L.S. and J.S. Weissman, *Nascent transcript sequencing visualizes transcription at nucleotide resolution.* Nature, 2011. **469**(7330): p. 368-73.
11. Nojima, T., et al., *Mammalian NET-Seq Reveals Genome-wide Nascent Transcription Coupled to RNA Processing.* Cell, 2015. **161**(3): p. 526-540.
12. Herzog, V.A., et al., *Thiol-linked alkylation of RNA to assess expression dynamics.* Nat Methods, 2017. **14**(12): p. 1198-1204.
13. Yu, A.D. and M. Rosbash, *Butt-seq: a new method for facile profiling of transcription.* Genes Dev, 2023. **37**(9-10): p. 432-448.
14. Schwalb, B., et al., *TT-seq maps the human transient transcriptome.* Science, 2016. **352**(6290): p. 1225-8.
15. Danko, C.G., et al., *Identification of active transcriptional regulatory elements from GRO-seq data.* Nat Methods, 2015. **12**(5): p. 433-8.
16. Wang, Z., et al., *Identification of regulatory elements from nascent transcription using dREG.* Genome Res, 2019. **29**(2): p. 293-303.
17. Azofeifa, J.G., et al., *An Annotation Agnostic Algorithm for Detecting Nascent RNA Transcripts in GRO-Seq.* IEEE/ACM Trans Comput Biol Bioinform, 2017. **14**(5): p. 1070-1081.

18. Chae, M., C.G. Danko, and W.L. Kraus, *groHMM: a computational tool for identifying unannotated and cell type-specific transcription units from global run-on sequencing data.* BMC Bioinformatics, 2015. **16**: p. 222.
19. Allison, K.A., et al., *Vespucci: a system for building annotated databases of nascent transcripts.* Nucleic Acids Res, 2014. **42**(4): p. 2433-47.
20. Wang, Z., et al., *nASAP: A Nascent RNA Profiling Data Analysis Platform.* J Mol Biol, 2023. **435**(14): p. 168142.
21. Wang, J., et al., *Nascent RNA sequencing analysis provides insights into enhancer-mediated gene regulation.* BMC Genomics, 2018. **19**(1): p. 633.
22. Henninger, J.E. and R.A. Young, *An RNA-centric view of transcription and genome organization.* Mol Cell, 2024. **84**(19): p. 3627-3643.
23. Woodley, C.M., et al., *Multiple interactions of the oncoprotein transcription factor MYC with the SWI/SNF chromatin remodeler.* Oncogene, 2021. **40**(20): p. 3593-3609.
24. van Riggelen, J., A. Yetil, and D.W. Felsher, *MYC as a regulator of ribosome biogenesis and protein synthesis.* Nat Rev Cancer, 2010. **10**(4): p. 301-9.
25. Ramalho, S., A. Dopler, and W.J. Faller, *Ribosome specialization in cancer: a spotlight on ribosomal proteins.* NAR Cancer, 2024. **6**(3): p. zcae029.
26. Zacarias-Fluck, M.F., L. Soucek, and J.R. Whitfield, *MYC: there is more to it than cancer.* Front Cell Dev Biol, 2024. **12**: p. 1342872.
27. Wolpaw, A.J., et al., *Drugging the "Undruggable" MYCN Oncogenic Transcription Factor: Overcoming Previous Obstacles to Impact Childhood Cancers.* Cancer Res, 2021. **81**(7): p. 1627-1632.
28. Popay, T.M., et al., *MYC regulates ribosome biogenesis and mitochondrial gene expression programs through its interaction with host cell factor-1.* Elife, 2021. **10**.
29. Elizarraras, J.M., et al., *WebGestalt 2024: faster gene set analysis and new support for metabolomics and multi-omics.* Nucleic Acids Res, 2024. **52**(W1): p. W415-W421.
30. Subramanian, A., et al., *Gene set enrichment analysis: a knowledge-based approach for interpreting genome-wide expression profiles.* Proc Natl Acad Sci U S A, 2005. **102**(43): p. 15545-50.
31. Reyes, A. and W. Huber, *Alternative start and termination sites of transcription drive most transcript isoform differences across human tissues.* Nucleic Acids Res, 2018. **46**(2): p. 582-592.
32. Pan, Q., et al., *Deep surveying of alternative splicing complexity in the human transcriptome by high-throughput sequencing.* Nat Genet, 2008. **40**(12): p. 1413-5.
33. Carninci, P., et al., *Genome-wide analysis of mammalian promoter architecture and evolution.* Nat Genet, 2006. **38**(6): p. 626-35.
34. Tian, B. and J.L. Manley, *Alternative polyadenylation of mRNA precursors.* Nat Rev Mol Cell Biol, 2017. **18**(1): p. 18-30.
35. Ferdinandusse, S., et al., *Peroxisomal fatty acid oxidation disorders and 58 kDa sterol carrier protein X (SCPx). Activity measurements in liver and fibroblasts using a newly developed method.* J Lipid Res, 2000. **41**(3): p. 336-42.
36. Stanley, W.A., et al., *Recognition of a functional peroxisome type 1 target by the dynamic import receptor pex5p.* Mol Cell, 2006. **24**(5): p. 653-663.

37. Puglielli, L., et al., *Sterol carrier protein-2 is involved in cholesterol transfer from the endoplasmic reticulum to the plasma membrane in human fibroblasts.* J Biol Chem, 1995. **270**(32): p. 18723-6.
38. Seedorf, U., et al., *Structure-activity studies of human sterol carrier protein 2.* J Biol Chem, 1994. **269**(4): p. 2613-8.
39. Vilborg, A., et al., *Widespread Inducible Transcription Downstream of Human Genes.* Mol Cell, 2015. **59**(3): p. 449-61.
40. Rutkowski, A.J., et al., *Widespread disruption of host transcription termination in HSV-1 infection.* Nat Commun, 2015. **6**: p. 7126.
41. Grosso, A.R., et al., *Pervasive transcription read-through promotes aberrant expression of oncogenes and RNA chimeras in renal carcinoma.* Elife, 2015. **4**.
42. Vilborg, A., et al., *Comparative analysis reveals genomic features of stress-induced transcriptional readthrough.* Proc Natl Acad Sci U S A, 2017. **114**(40): p. E8362-E8371.
43. Hennig, T., et al., *HSV-1-induced disruption of transcription termination resembles a cellular stress response but selectively increases chromatin accessibility downstream of genes.* PLoS Pathog, 2018. **14**(3): p. e1006954.
44. Vilborg, A. and J.A. Steitz, *Readthrough transcription: How are DoGs made and what do they do?* RNA Biol, 2017. **14**(5): p. 632-636.
45. Caldas, P., et al., *Transcription readthrough is prevalent in healthy human tissues and associated with inherent genomic features.* Commun Biol, 2024. **7**(1): p. 100.
46. Papadopoulos, D., et al., *MYCN recruits the nuclear exosome complex to RNA polymerase II to prevent transcription-replication conflicts.* Mol Cell, 2022. **82**(1): p. 159-176 e12.
47. Gregersen, L.H., et al., *SCAF4 and SCAF8, mRNA Anti-Terminator Proteins.* Cell, 2019. **177**(7): p. 1797-1813 e18.
48. Taylor, S.J., et al., *Pharmacological restriction of genomic binding sites redirects PU.1 pioneer transcription factor activity.* Nat Genet, 2024. **56**(10): p. 2213-2227.
49. Layden, H.M., et al., *Mutant FOXO1 controls an oncogenic network via enhancer accessibility.* Cell Genom, 2024. **4**(4): p. 100537.
50. Kelly, R.D.W., et al., *Histone deacetylases maintain expression of the pluripotent gene network via recruitment of RNA polymerase II to coding and noncoding loci.* Genome Res, 2024. **34**(1): p. 34-46.
51. Zhao, J., et al., *Inherited blood cancer predisposition through altered transcription elongation.* Cell, 2024. **187**(3): p. 642-658 e19.
52. Bressin, A., et al., *High-sensitive nascent transcript sequencing reveals BRD4-specific control of widespread enhancer and target gene transcription.* Nat Commun, 2023. **14**(1): p. 4971.
53. Bomber, M.L., et al., *Human SMARCA5 is continuously required to maintain nucleosome spacing.* Mol Cell, 2023. **83**(4): p. 507-522 e6.
54. Zhang, S., et al., *PAX3-FOXO1 coordinates enhancer architecture, eRNA transcription, and RNA polymerase pause release at select gene targets.* Mol Cell, 2022. **82**(23): p. 4428-4442 e7.
55. Mahat, D.B., et al., *Single-cell nascent RNA sequencing unveils coordinated global transcription.* Nature, 2024. **631**(8019): p. 216-223.

56. Love, M.I., W. Huber, and S. Anders, *Moderated estimation of fold change and dispersion for RNA-seq data with DESeq2.* Genome Biol, 2014. **15**(12): p. 550.
57. Villanueva, R.A.M. and Z.J. Chen, *ggplot2: Elegant Graphics for Data Analysis, 2nd edition.* Measurement-Interdisciplinary Research and Perspectives, 2019. **17**(3): p. 160-167.
58. Robinson, J.T., et al., *Integrative genomics viewer.* Nat Biotechnol, 2011. **29**(1): p. 24-6.
59. Ramirez, F., et al., *deepTools2: a next generation web server for deep-sequencing data analysis.* Nucleic Acids Res, 2016. **44**(W1): p. W160-5.

Figures

Figure 1. Summary of features distinguishing eNRSA from exiting nascent RNA sequencing analysis tools.

Figure 2. Workflow of eNRSA.

Figure 3. eNRSA enhances differential analysis by removing the batch effect. (A) PCA plot of normalized counts mapped to gene body for DMSO and dTAG47-treated cells from two batches. (B) The number of dysregulated genes identified by NRSA and eNRSA with an FDR<0.05. (C) Volcano plot showing the $\log_2$ fold change (x-axis) and $-\log_{10}$ FDR (y-axis) for dTAG47-treated vs. DMSO-treated on gene body transcription. (D) Pathways enriched in the downregulated genes in dTAG47-treated vs. DMSO-treated cells by WebGestalt 2024. (E) GSEA results revealing the two most significant pathways, MYC targets and rRNA processing, enriched in the downregulation of dTAG47-treated cells compared to DMSO.

Figure 4. Example of ATSS and readthrough dysregulation identified by eNRSA. (A) IGV screenshot of PRO-seq and RNA-seq signals illustrating ATSS events between G401 and Ramos cells. In the G401 cell line, promoter-proximal peaks were observed at two TSS sites, while only one single peak was observed at the second TSS site in the Ramos cell line. (B) Metagene profiles of PRO-seq data for the genes with increased (left) and decreased (right) readthrough in OmoMYC vs. EGFP.

Figure 5. Performance comparison between eNRSA and NRSA. (A) Runtime with increasing numbers of PRO-seq reads. (B) Memory usage with increasing numbers of PRO-seq reads. (C) Runtime with increasing numbers of transcripts defined in the GTF file. (D) Memory usage with increasing numbers of transcripts defined in the GTF file. Computations were performed using a single thread of an Intel Xeon E5-2695 v4 @ 2.10GHz processor with 1T memory.

Figures
Click here to access/download;Figure;eNRSA-Figures.pdf

Fig. 1

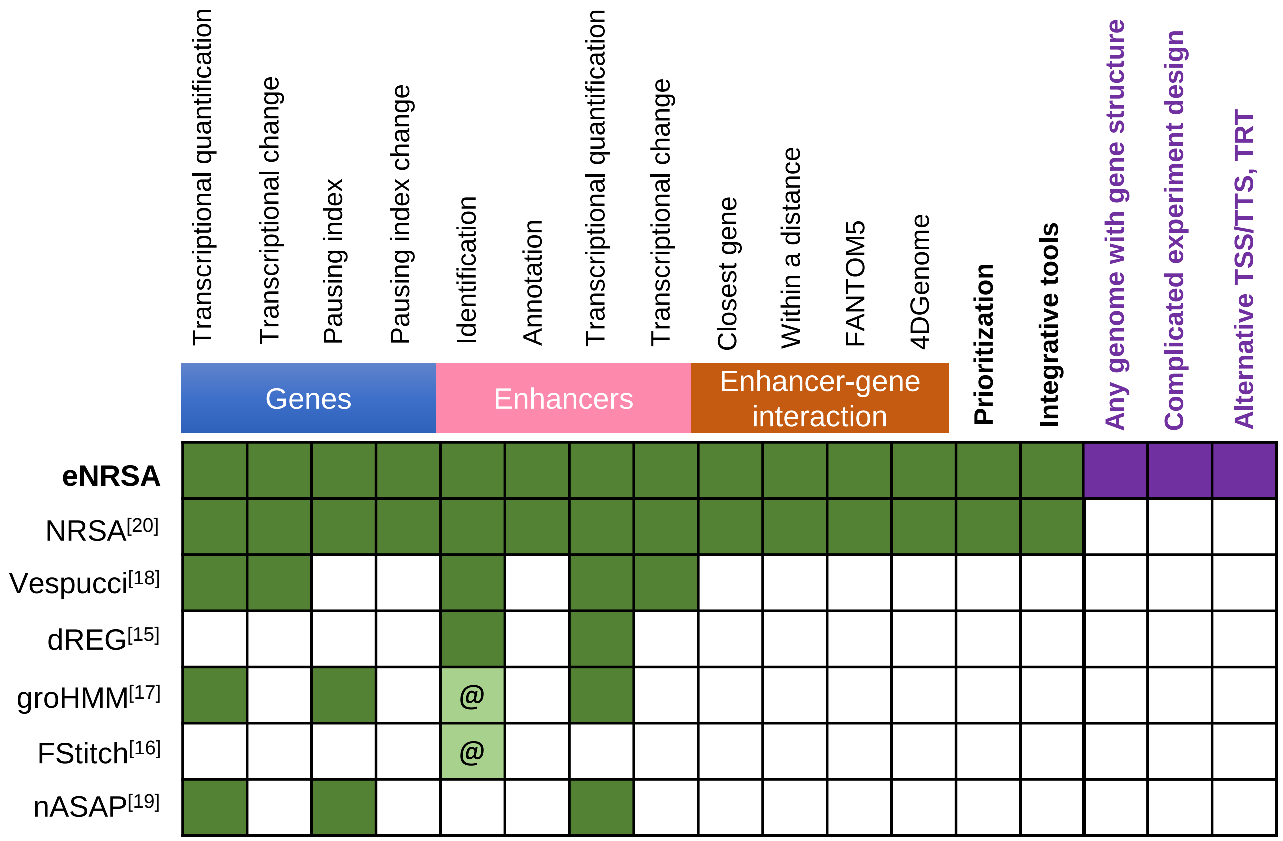

New features in eNRSA

@ identify novel transcripts instead of enhancers, but novel transcripts could be further classified into enhancers with the help of other scripts or genome annotation.

Fig. 2

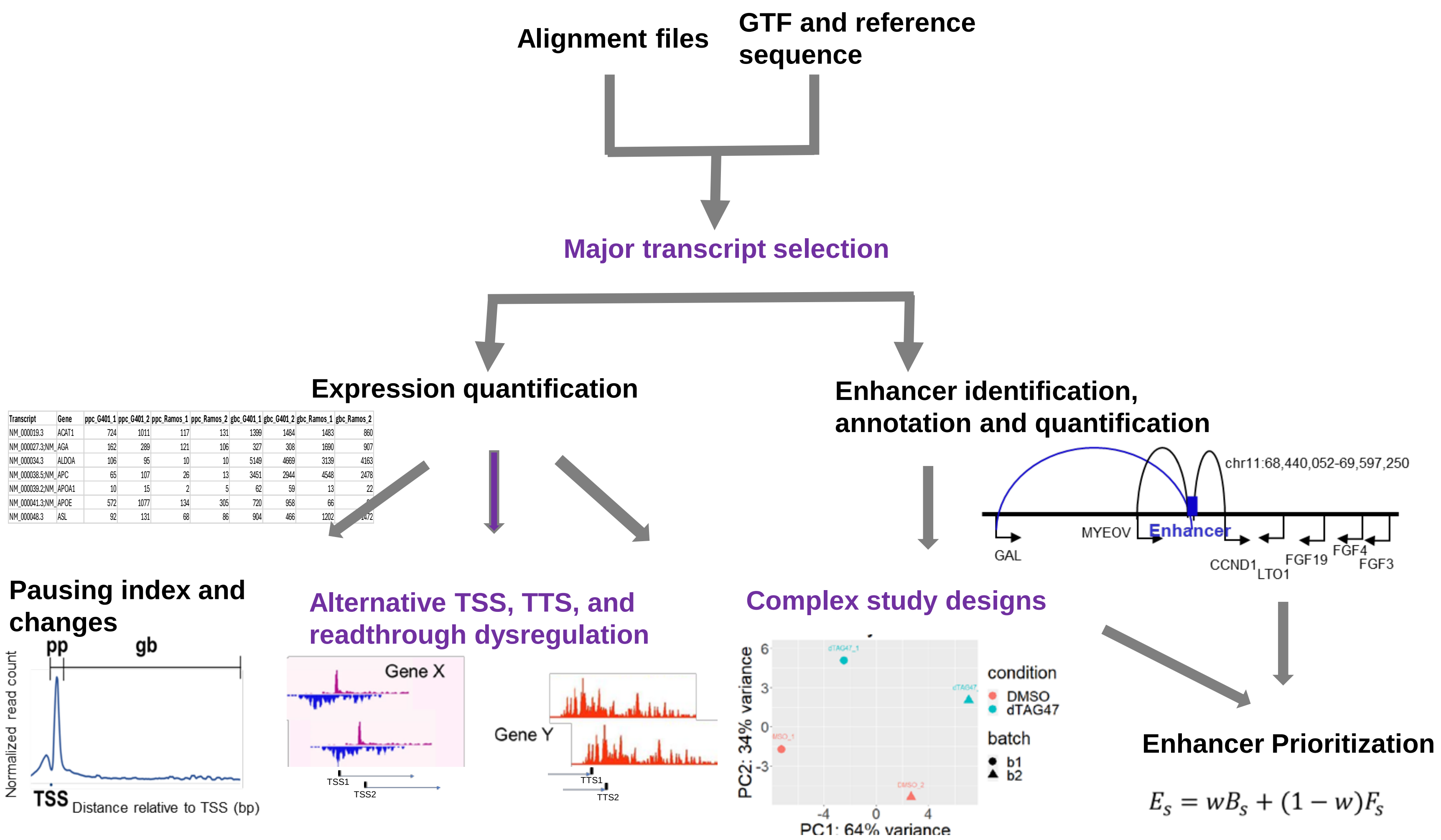

Alignment files
GTF and reference sequence
Major transcript selection
Expression quantification
Enhancer identification, annotation and quantification
chr11:68,440,052-69,597,250
GAL
MYEOV
Enhancer
CCND1
LTO1
FGF19
FGF4
FGF3
Pausing index and changes
pp
gb
Normalized read count
TSS
Distance relative to TSS (bp)
Alternative TSS, TTS, and readthrough dysregulation
Gene X
TSS1
TSS2
Gene Y
TTS1
TTS2
Complex study designs
PC2: 34% variance
PC1: 64% variance
condition
DMSO
dTAG47
batch
b1
b2
Enhancer Prioritization
$E_s = wB_s + (1 - w)F_s$

Fig. 2

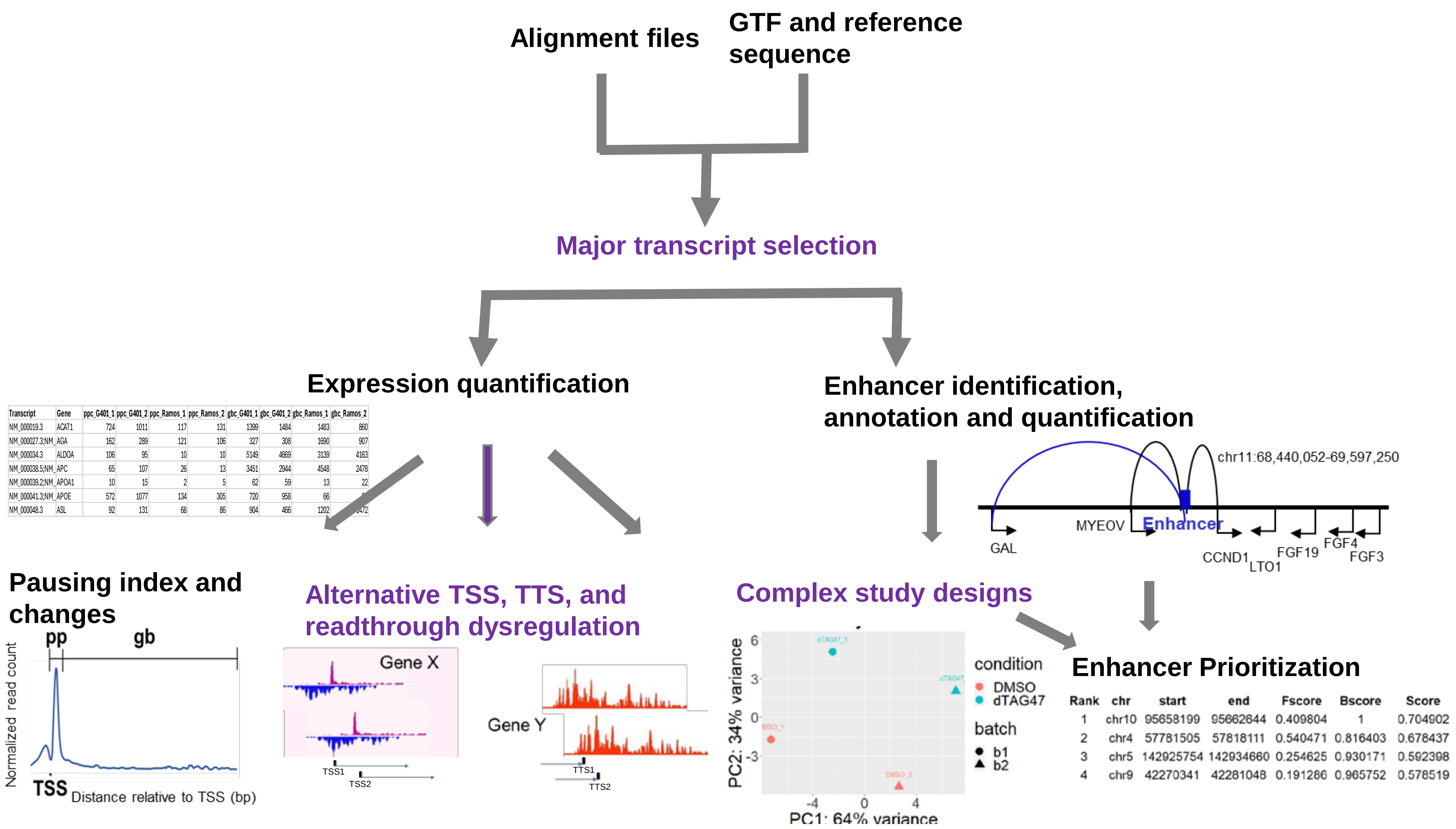

Rank	chr	start	end	Fscore	Bscore	Score
1	chr10	95658199	95662644	0.409804	1	0.704902
2	chr4	57781505	57818111	0.540471	0.816403	0.678437
3	chr5	142925754	142934660	0.254625	0.930171	0.592398
4	chr9	42270341	42281048	0.191286	0.965752	0.578519

Fig. 3

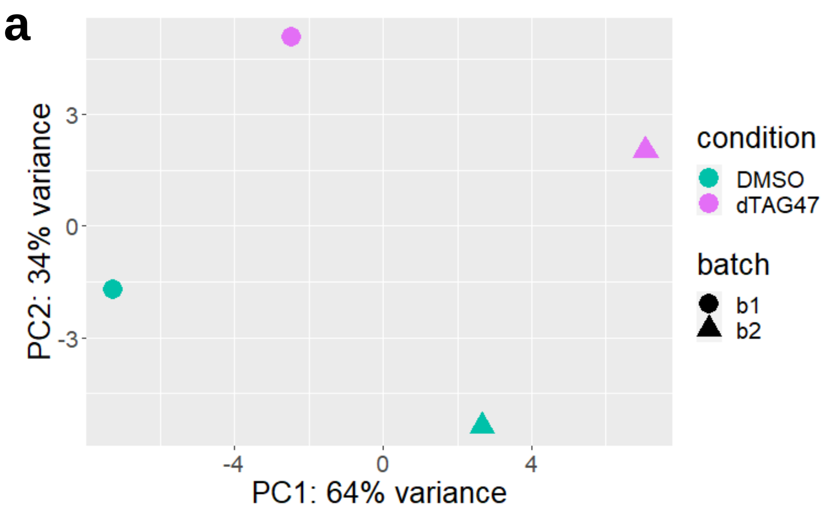

b

	NRSA	eNRSA
**Up**	5	440
**Down**	62	829

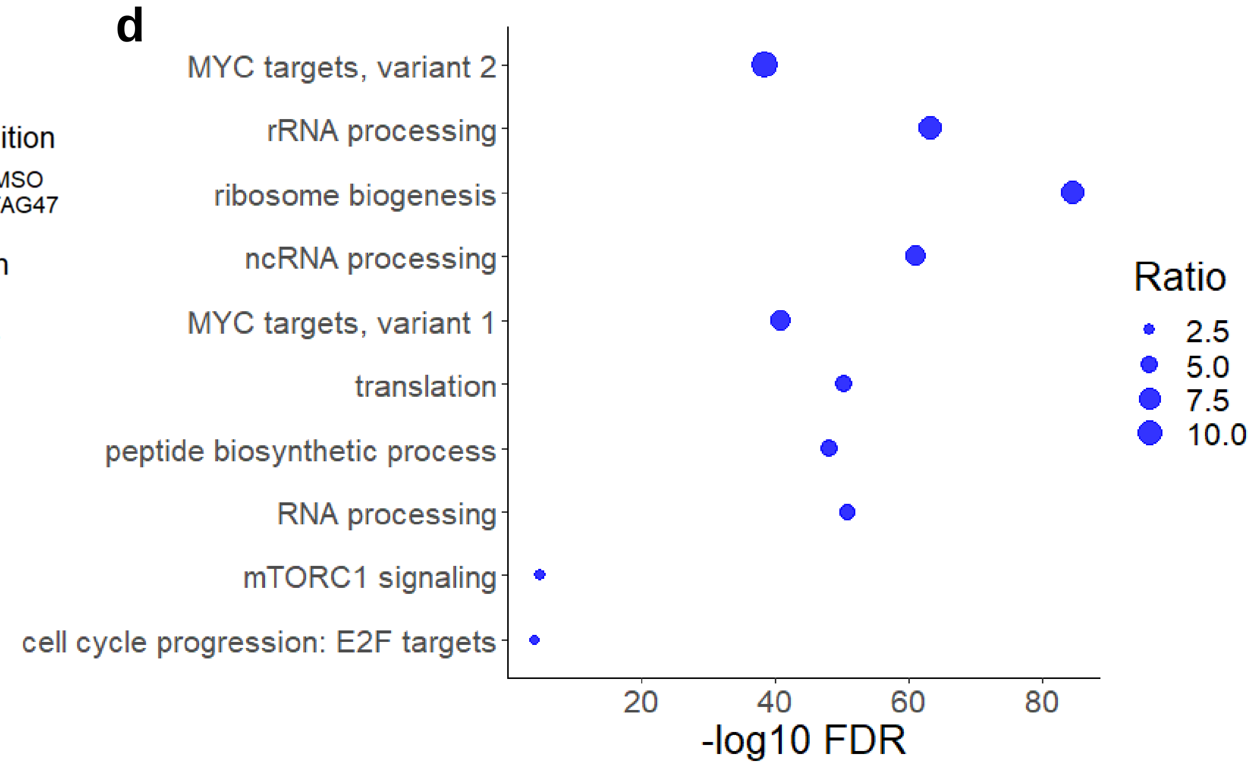

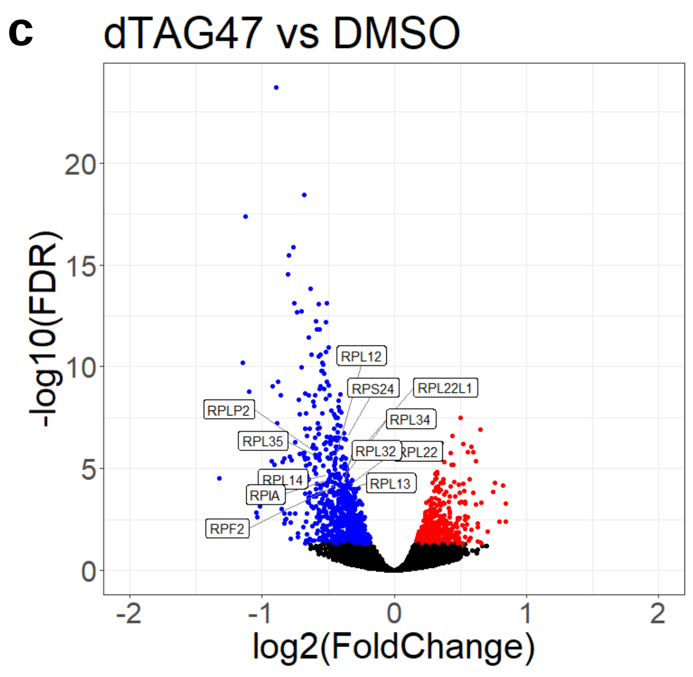

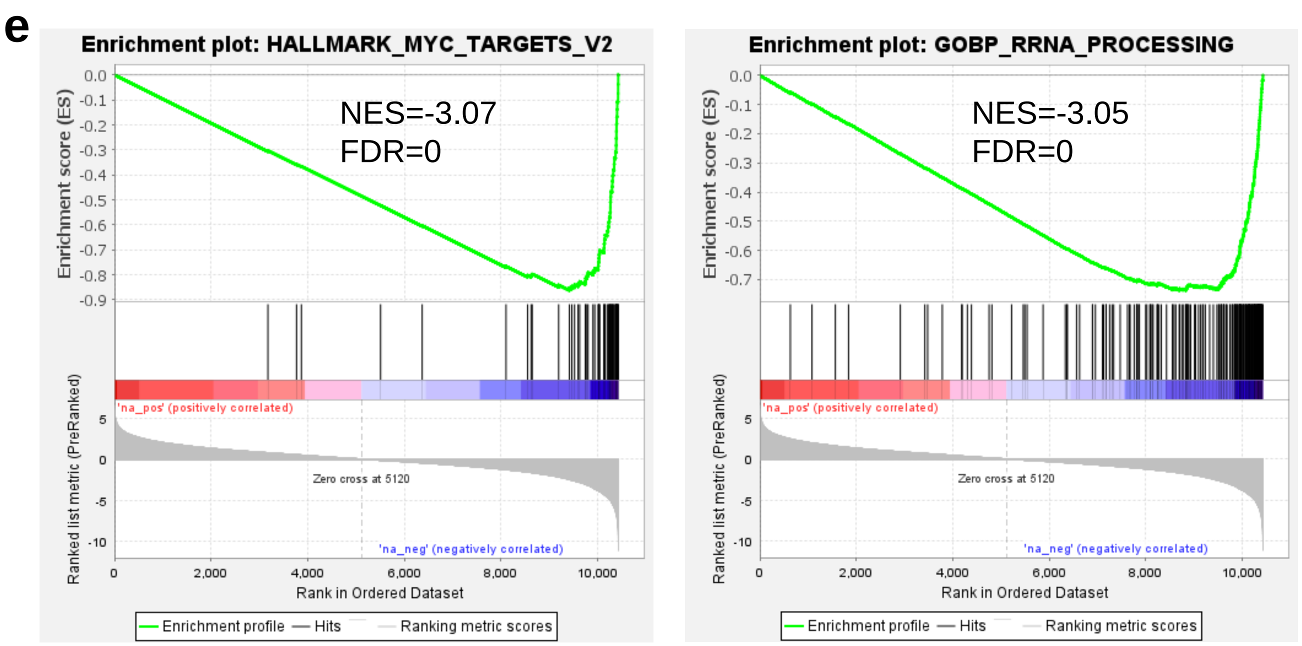

Fig. 4

a

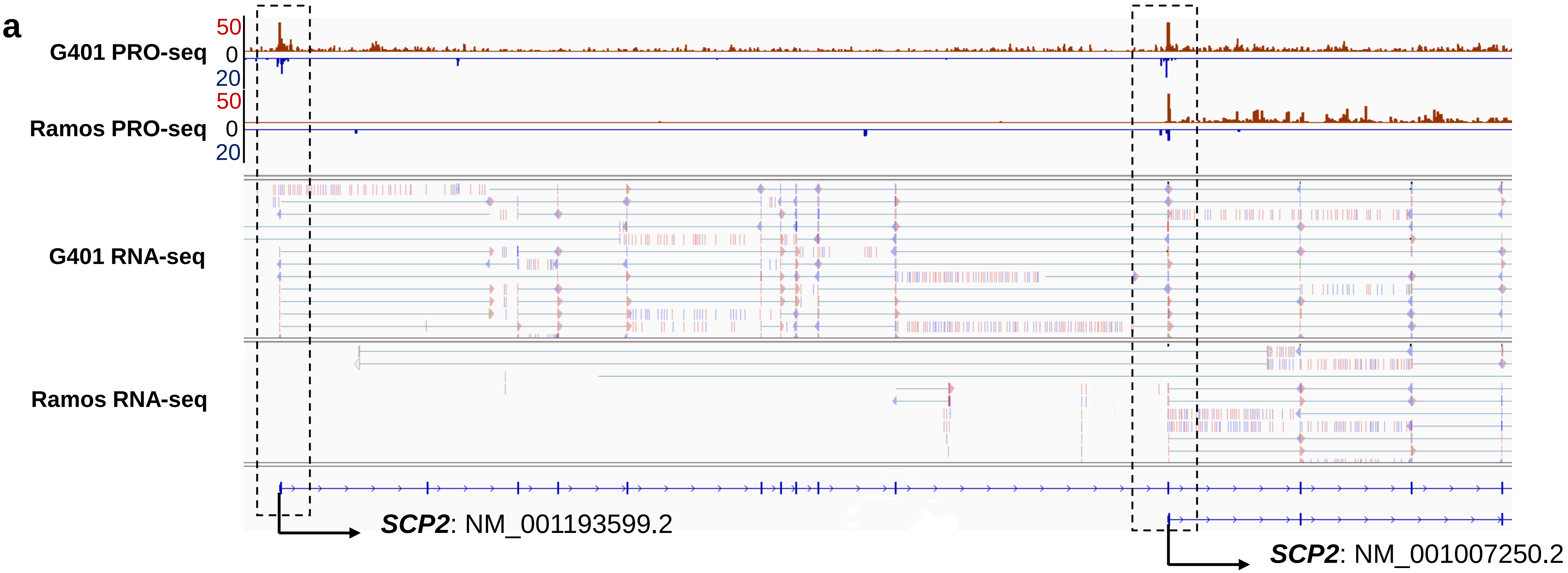
G401 PRO-seq
Ramos PRO-seq
G401 RNA-seq
Ramos RNA-seq
50
0
20
SCP2: NM_001193599.2
SCP2: NM_001007250.2

b

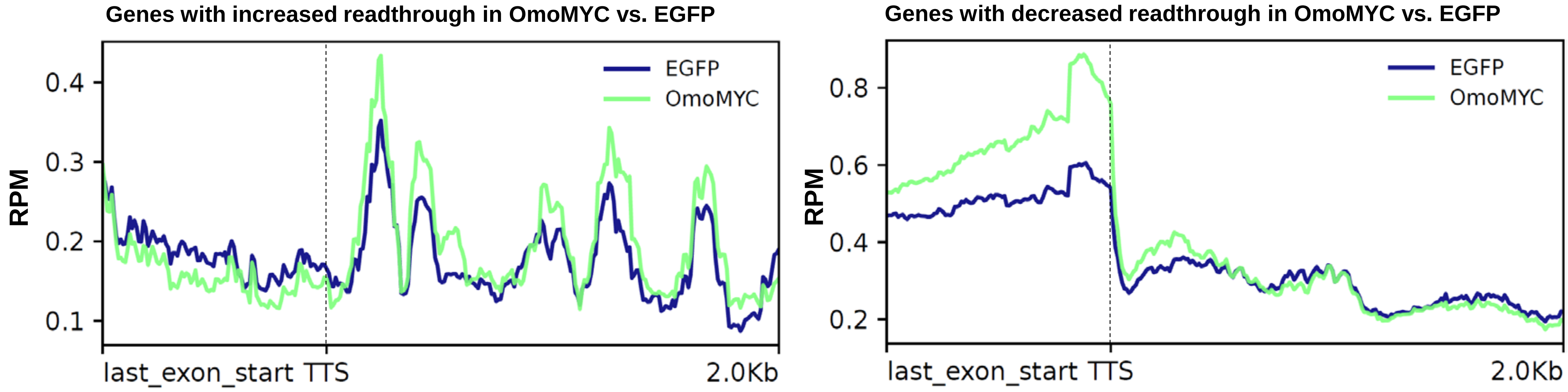
Genes with increased readthrough in OmoMYC vs. EGFP
Genes with decreased readthrough in OmoMYC vs. EGFP
RPM
EGFP
OmoMYC
0.1
0.2
0.3
0.4
0.6
0.8
last_exon_start TTS
2.0Kb

Fig. 5

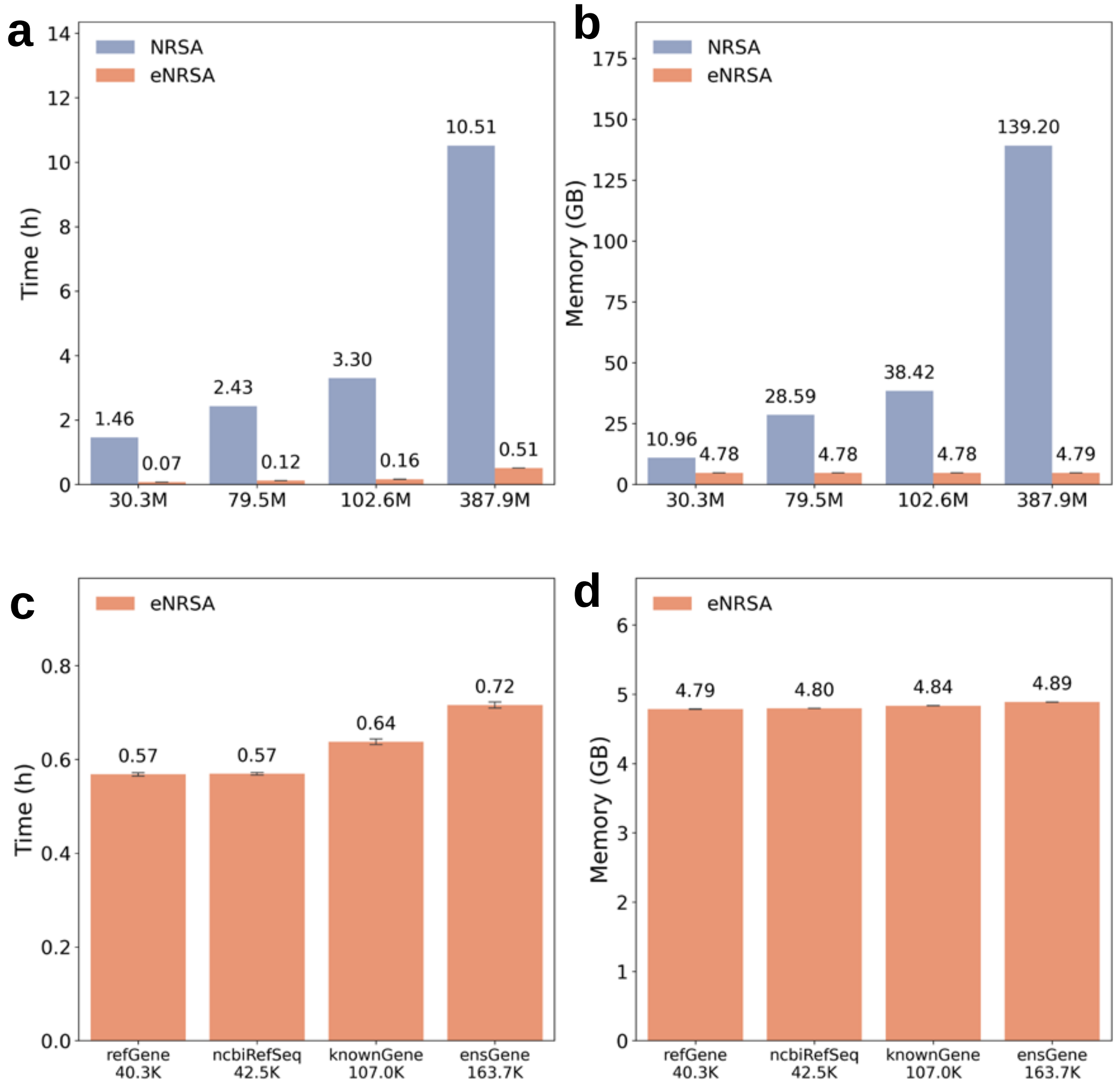
a
b
c
d
NRSA
eNRSA
Time (h)
Memory (GB)
1.46
0.07
2.43
0.12
3.30
0.16
10.51
0.51
30.3M
79.5M
102.6M
387.9M
10.96
4.78
28.59
4.78
38.42
4.78
139.20
4.79
0.57
0.57
0.64
0.72
4.79
4.80
4.84
4.89
refGene 40.3K
ncbiRefSeq 42.5K
knownGene 107.0K
ensGene 163.7K

Click here to access/download
Supplementary Material
eNRSA-SupplTables.xlsx

Cover Letter Click here to access/download;Personal Cover;coverLetter.docx

VANDERBILT UNIVERSITY MEDICAL CENTER

Qi Liu, PhD.
Professor, Department of Biostatistics
Vanderbilt University Medical Center
Nashville, TN, USA
Tel: 615-322-6618
Email: qi.liu@vumc.org

Dear Editor,

We are pleased to submit our manuscript entitled **"eNRSA: A Faster and More Powerful Approach for Nascent Transcriptome Analysis"** for consideration for publication in *GigaScience*. All authors have approved the manuscript for submission and declare no conflicts of interest. This work has not been submitted elsewhere.

Nascent RNA sequencing provides the distinct advantage of directly measuring new RNA production, making it a critical tool for studying immediate regulatory changes in both genes and enhancers. In 2018, we developed NRSA, which has since been widely used for nascent transcriptome analysis. However, NRSA's applicability is limited by its restriction to specific organisms, its lack of support for multi-factor experimental designs, and its high time and memory requirements.

Here, we present **eNRSA**, a significantly enhanced version of NRSA. eNRSA is 20 times faster than NRSA and requires approximately 1/8 of the memory. It improves analytical performance by adaptively selecting major transcripts and by supporting multi-factor experimental designs. These advancements make eNRSA applicable to any organism or genome with a known gene structure. Furthermore, eNRSA introduces novel functionalities, including the ability to identify alternative transcription start sites (ATSS), alternative transcription termination sites (ATTS), and readthrough dysregulation across conditions. By fully leveraging the unique characteristics of nascent transcriptomes, eNRSA addresses limitations in existing tools. The software is freely available at https://bioinfo.vanderbilt.edu/eNRSA/.

eNRSA has already been evaluated and tested by multiple external users, who initially reached out to request extensions to NRSA's functionalities. Based on their feedback and the growing demand for such a tool in the field, we are confident that eNRSA will

become a widely used and impactful resource for nascent transcriptome analysis.

We appreciate your consideration of our manuscript and look forward to the reviewers' feedback. Should you have any questions or require further information, please do not hesitate to contact me.

Thank you very much for your time!

Best regards,

Qi Liu

potential peer reviewers

1. Don Delker, Ph.D.
don.delker@nih.gov
NIH - national institute of health
Integrative Bioinformatics Support Group

2. Aniket Kumar, PhD,
ak3@nibmg.ac.in
National Institute of Biomedical Genomics (NIBMG), Kalyani, India

3. Ulrich G. Steidl, M.D., Ph.D.
ulrich.steidl@einsteinmed.edu
Albert Einstein College of Medicine
Jack and Pearl Resnick Campus
