# Supplementary material for: eNRSA: a faster and more powerful approach for nascent transcriptome analysis
Source: Gigascience. 2025 Jul 4;14:giaf071. doi: 10.1093/gigascience/giaf071 (PMC12231571; doi:10.1093/gigascience/giaf071)
Supplement: giaf071_GIGA-D-25-00028_Revision_1 [file giaf071_giga-d-25-00028_revision_1.pdf]

GigaScience

eNRSA: A Faster and More Powerful Approach for Nascent Transcriptome Analysis

--Manuscript Draft--

**Manuscript Number:**	GIGA-D-25-00028R1
**Full Title:**	eNRSA: A Faster and More Powerful Approach for Nascent Transcriptome Analysis
**Article Type:**	Technical Note
**Funding Information:**	National Cancer Institute (CA200709, C247833) — Professor William P. Tansey<br>National Cancer Institute (CA229123, CA274367) — Professor Qi Liu<br>National Institutes of Health (AI139449) — Professor Qi Liu<br>Cancer Center Support Grant (CA068485) — Professor Qi Liu<br>Department of Biostatistics in VUMC (2024/2025 Biostatistics Faculty Development Award) — Dr. Jing Wang
**Abstract:**	Nascent RNA sequencing tracks primary transcriptional events, making it crucial for studying the immediate regulatory changes of genes and enhancers in response to both endogenous and exogenous stimuli. NRSA is a widely used tool for analyzing nascent transcriptomic data, enabling quantification of transcriptional changes at proximal promoters and gene bodies, estimation of pausing indices, identifying active enhancers, and establishing enhancer–target gene relationships. To improve its functionality and broaden its applicability to diverse organisms and complex study designs, we have developed an enhanced version, eNRSA. Key advancements include adaptive selection of major transcripts, support for any organism with known gene structures, compatibility with complex study designs, and identification of alternative transcription start and termination sites, as well as transcription readthrough events. Additionally, eNRSA achieves a ~20-fold increase in analysis speed while significantly reducing memory usage. These enhancements make eNRSA a faster, more versatile, and more powerful tool for nascent transcriptome analysis. eNRSA is freely available at https://bioinfo.vanderbilt.edu/eNRSA/.
**Corresponding Author:**	Qi Liu, Ph.D.<br>Vanderbilt University Medical Center<br>Nashville, TN UNITED STATES
**Corresponding Author Secondary Information:**	
**Corresponding Author's Institution:**	Vanderbilt University Medical Center
**Corresponding Author's Secondary Institution:**	
**First Author:**	Jing Wang
**First Author Secondary Information:**	
**Order of Authors:**	Jing Wang<br>Hua-chang Chen<br>Scott W. Hiebert<br>Quanhu Sheng<br>William P. Tansey<br>Yu Shyr<br>Qi Liu, Ph.D.
**Order of Authors Secondary Information:**	

Powered by Editorial Manager® and ProduXion Manager® from Aries Systems Corporation

Response to Reviewers:	GIGA-D-25-00028<br>eNRSA: A Faster and More Powerful Approach for Nascent Transcriptome Analysis<br><br>Reviewer #1<br>The manuscript by Wang et al reports a tool (eNRSA) for analysis of nascent transcriptomic data which can be generated by various methods such as GRO-seq, PRO-seq, TT-seq etc. This tool is advanced version of the NRSA tool which is already available. Along with the optimization in the functionality of NRSA authors have added new features which includes detection of Alternative Transcription Start site (ATSS), Alternative Transcription Termination Site (ATTS), inclusion of complex experimental design and flexibility to choose multiple organisms. The NRSA is already a great tool. This new tool will allow users to do analysis of nascent RNA transcriptome more efficiently even with limited computational resources. However there are few concerns which needs to be addressed to make the manuscript better and more comprehensive to the readers and the potential future users of the new tools.<br>Response: We greatly appreciate the reviewer's insightful comments and valuable suggestions. We have made every effort to address each point thoroughly. Specifically, we applied eNRSA to various nascent transcription datasets, including GRO-seq (Supplementary Table S6), mNET-seq (Supplementary Fig. S1), and Butt-seq (Supplementary Fig. S2), and proved the universal performance and robustness of eNRSA. We also validated ATSS and ATTS events identified by eNRSA through comparison with RNA-seq data (Fig. 4A, and Supplementary Tables S3 and S4). Furthermore, we have revised the manuscript to clarify methodological details regarding TSS/TTS site selection, readthrough calculation, and the rationale for using the Cochran-Mantel-Haenszel (CMH) test.<br><br>1. Authors have mentioned that this tool is compatible with nascent transcriptomic data generated by multiple experiments such as GRO-seq, PRO-seq, TT-seq, NET-seq etc but they have used only PRO-seq data for evaluating the tool. Authors should at least include data of few different types of experiments to prove the universal performance and robustness of their tool. Also they should mention the accuracy of the analysis for at least few different experiment types.<br>Response: We thank the reviewer for the great suggestion. In the revised manuscript, we have applied eNRSA to various types of nascent transcription experiments, including GRO-seq (Supplementary Table S6), mNET-seq (Supplementary Fig. S1), and Butt-seq (Supplementary Fig. S2), where results are consistent with the findings reported in the original publications [1-3] (Page 8). These applications further prove the universal performance and robustness of eNRSA. Furthermore, we discussed the appropriate interpretation of eNRSA results when analyzing data from other nascent protocols, such as SLAM-seq and TT-seq (Pages 7-8).<br>Since there is no gold standard for nascent transcriptomic data analysis, evaluating the accuracy of eNRSA is challenging. We first demonstrated that eNRSA produces consistent results with the findings reported in the original publications across various nascent transcriptome platforms (Supplementary Figs. S1 and S2 and Supplementary Table S6). Additionally, we permuted PRO-seq reads between DMSO and dTAG47-treated cells (100 permutations) to generate a background distribution, which was used to estimate the probability of obtaining the observed difference by chance. Using the permutation-based test as the gold standard, eNRSA achieved 80.1% sensitivity, 100% specificity, and 95.6% accuracy. We included this in the manuscript (Page 5).<br><br>2. Authors have mentioned that eNRSA can detect ATSS and ATTS. They have also mentioned that due to the nature of data of nascent transcriptome all ATSS and ATTS cannot be identified. Thus they should provide a more detailed comparison of the percentage of ATSS and ATTS identified with respect to isoform analysis performed using total RNA seq data of the same cell line or sample to show the robustness of the tool in this particular application.<br>Response: We thank the reviewer for this insightful suggestion. In response, we compared the ATSSs and ATTSs identified from the nascent transcriptome with those detected from RNA-seq data. Given the complexity of transcriptional regulation and the fact that nascent transcriptome and RNA-seq capture different layers of transcription, we observed a strong overlap between the two datasets. Specifically, of the 1,405 ATSSs identified from the nascent transcriptome, 1,054 (75.0%) were also supported by RNA-seq. Similarly, of the 905 ATTSs detected in the nascent data, 736 (81.3%) were confirmed by RNA-seq (Fig. 4A in the manuscript). This strong concordance

highlights the robustness of eNRSA in identifying ATSSs and ATTSs across transcriptomic layers. We have added this comparison to the manuscript (Fig. 4A, Pages 6 and 13).

3. Authors have mentioned in the method section that Homer package is required as one of the dependencies. That implies that the eRNA detection is done using the Homer package. Author should mention this in detail in the manuscript.
Response: We thank the reviewer for pointing this out. We have now included a detailed description of the eRNA detection and enhancer identification procedure using the HOMER package in the revised manuscript (Pages 10-11), to clarify its role as a dependency and how it is used in eNRSA.

4. In supplementary table S3 and S4 eNRSA have only compared the counts in the two alternate transcription start site (TSS) and Transcripion termination site (TTS) in two different condition. But in reality more than two TSS and TTS also exists. Although authors have mentioned this point as limitation but authors should clarify that if more than two TSS and TTS exists then on what basis two TSS and TTS will be chosen by the software?
Response: We apologize for the misunderstanding. eNRSA does not select only two TSS or TTS sites for comparison. Instead, for each gene, all TSS sites are considered, if they are separated by at least 1,000 bp to reduce signal interference and ensure reliable detection. For example, if a gene has three TSSs (TSS1, TSS2, TSS3), eNRSA evaluates usage shifts between all eligible pairs: TSS1 vs. TSS2, TSS1 vs. TSS3, and TSS2 vs. TSS3—excluding closely spaced TSSs (<1,000 bp apart). Similarly, for each gene, all TTS sites are considered, provided they do not overlap with any annotated gene and there is no gene within 3 kb downstream, to reduce transcriptional noise from neighboring genes.
In Supplementary Tables S3 and S4, multiple TSS and TTS shift events from the same gene are included. For instance, in Table S3, two ATSS events are reported for the RGS3 gene: one between chr9:116343157 and chr9:116356200, and another between chr9:116343157 and chr9:116356434.
We have clarified this in the revised manuscript (Page 11).

5. Authors have used read counts at TSS and TTS and used GTF to detect the major transcript and to identify ATSS and ATTS. The software is using Cochran-Mantel Haenszel test (CMH). Authors have mentioned very briefly about this test even in the method section. Authors should include some more points to justify the use of this test and why this will generate robust results.
Response: We thank the reviewer for the constructive comment. We have expanded the Methods section (Page 11) to provide a more detailed justification for the use of the Cochran-Mantel-Haenszel (CMH) test.
Rather than pooling data across samples, which may obscure biological variability, we use the Cochran-Mantel-Haenszel (CMH) test to perform a stratified analysis. This test evaluates whether there is a consistent association between TSS usage and condition across sample pairs, while controlling for sample-specific variability. Under the assumption of a common odds ratio across strata, the CMH test increases statistical power and reduces bias due to heterogeneity among samples. This framework ensures robust detection of systematic shifts in TSS usage across biological replicates.

6. Authors have not mentioned the window size that they have used 50kb downstream of TTS to calculate readthrough dysregulation.
Response: We apologize for the confusion. In eNRSA, transcriptional readthrough is quantified as the ratio of the number of reads mapped within a fixed 50 kb region downstream of the annotated TTS to the number of reads mapped within the last exon [4]. A fixed 50 kb region was used, without applying any sliding window or variable window size. This approach was adapted from a previous study on transcriptional readthrough [4]. We have revised the Methods section to clarify this point (Pages 7, 12).
Minor concern 1. The figure 2 is repeated 2 times. PCA plot subfigure in figure 2 is cropped more than needed.
Response: We thank the reviewer for pointing this out. The duplicate Figure 2 has been removed, and the PCA plot in Figure 2 has been re-cropped to better display the data without excessive trimming.

Reviewer #2
In this article the authors create eNRSA, a pipeline for analysing nascent RNA sequening data. This pipeline has clear benefits to the community as it is able to note changes in isoforms that are detectable in the data and take in complex experimental designs. The authors show it is also faster than their original pipeline. The main weaknesses are in a discussion of the 3' end of the gene. However, the transcription termination site seems to be an all-encompassing term, with very little biological meaning. Other than this point the paper is well written and ready for publication.
Response: We deeply appreciate the reviewer for recognizing the value of eNRSA and for the insightful comments regarding its application to different nascent transcription protocols, the clear definition of TTS, and the detection of transcriptional readthrough. In response, we have discussed the appropriate interpretation of eNRSA results when analyzing various nascent transcription protocols. Additionally, we have revised the manuscript to clarify the definition of TTS and to highlight the performance of eNRSA in detecting readthrough events, particularly when polymerase dissociation fails.

Areas for Improvement
Major Issues:
•There are many Nascent RNA-seq protocols. Some of them are more "nascent" than others. For instance, since NET-seq is a pull-down of RNA polymerase, it is one of the most nascent. Similarly, PRO-seq is based on nt incorporated into the nascent RNA, and there is almost nonexistent time for cellular RNA degradation or splicing. It is one of the most nascent protocols. Protocols such as SLAM-seq, encorportate 4-thiouracil for 15 min with live cells, allowing the RNA-degradation machinery to act on transcripts in those 15 minutes. The longer the cells are incubated with 4-thiouracil, the more transcripts degrade. See the paper "A comparison of experimental assays and analytical methods for genome-wide identification of active enhancers" for more information. The authors should mention this variability as it relates to the differential results they get from their assay. For instance, I can imagine a scenario where GRO-seq could show similar 5' ends between two samples, but SLAM-seq could show different 5' ends as there is an RNA stability component to SLAM-seq. Therefore, the authors need to clarify how the choice of nascent protocol affects the interpretation of the eNRSA results.
Response: We thank the reviewer for the insightful comment. We have clarified the appropriate interpretation of eNRSA results when analyzing data from various nascent protocols in the manuscript (Pages 8-9 and below).
Although eNRSA is primarily designed to analyze GRO-seq or PRO-seq data, it can also be applied to data generated by other nascent RNA sequencing technologies that capture transcriptional dynamics, including pausing, elongation, and termination, such as Butt-seq, NET-seq, and mNET-seq. Examples of these applications are provided in Supplementary Table S6, Figs. S1 and S2, where the results are consistent with the findings reported in the original publications [1-3]. For nascent RNA sequencing technologies that capture only specific aspects of transcriptional dynamics, eNRSA results should be interpreted with caution. For instance, SLAM-seq tracks RNA kinetics including transcription, processing and degradation, and metabolically labeled RNAs are subject to degradation over time [5]. Therefore, while transcriptional changes within the gene body identified by eNRSA may still be meaningful, results related to pausing, ATSS/ATTS, and readthrough may not be interpretable—either because SLAM-seq does not capture pausing dynamics or due to RNA degradation leading to limited coverage beyond transcription end sites. In contrast, TT-seq maps the entire range of transient RNA and monitors RNA synthesis and degradation, which doesn't yield peak signals near the promoter where polymerase pauses like GRO/PRO-seq, NET-seq, Butt-seq, or mNET-seq [6]. Therefore, eNRSA results on gene body, ATTS, and readthrough may be informative, but those related to pausing may not be applicable.

•The same issue of RNA stability is significant in their alternative TTS section. Moreover, it is entirely unclear what TTS means in this paper. From the RNA point of view, the cleavage position, which is just downstream of the PAS, is the transcript's end. This is also the annotated "end" of the gene. However, from the Pol II perspective, the position Pol II dissociates from the DNA is at the end of the transcript, and the dissociation position is 1-30kb downstream of the annotated end of the gene. Therefore, what the authors call "readthrough" is the typical and expected detection of reads downstream of the cleavage position. In PRO-seq assays, RNA in the

Powered by Editorial Manager® and ProduXion Manager® from Aries Systems Corporation

	readthrough region is expected, but less of these should be present in SLAM-seq assays as the RNA downstream of the cleavage position is quickly degreed in a typical cell. To publish this paper, the authors must clearly explain their definition of TTS and what it means biologically based on the nascent assay being used. There is a peak at the cleavage position, but a comparison of the cleavage and the dissociation positions shows that the most substantial peak is at the dissociation position. For more information, please see “TT-seq maps the human transient transcriptome”, “FStitch: A fast and simple algorithm for detecting nascent RNA transcripts”, “Mechanisms of RNA Polymerase II Termination at the 3′-End of Genes”, “Knowing when to stop: Transcription termination on protein-coding genes by eukaryotic RNAPII”.<br>Response: We appreciate the reviewer’s comment and apologize for the lack of clarity regarding our definition of TTS. We have now clarified this in the revised manuscript (Page 6). Specifically, in eNRSA, TTS (transcription termination site) refers to the annotated end of the gene, defined as the 3′ end of the last exon in the GTF file, marking the cleavage and polyadenylation site of the mature RNA transcript.<br>As the reviewer pointed out, for a Polymerase II (Pol II) perspective, transcription termination does not occur immediately at this cleavage site. Instead, Pol II continues transcribing downstream, and dissociation typically occurs 1–50 kb downstream [4, 6, 7]. Therefore, eNRSA defines “readthrough” as transcriptionally engaged Pol II detected downstream of the annotated TTS/cleavage site and extending to the Pol II dissociation zone. This readthrough signal is expected in nascent transcription assays such as PRO-seq, GRO-seq, NET-seq, mNET-seq, Butt-seq, which capture engaged polymerase II, or TT-seq, which also maps transient RNA downstream of polyadenylation sites [6]. In contrast, assays like SLAM-seq, which detect metabolically labeled RNAs post-transcriptionally, are less likely to show signal downstream of the cleavage site due to rapid degradation of unprocessed or unstable transcripts in the readthrough region [5] (Page 7).<br><br>•When cells are stressed, such as heat shock or osmotic stress, cleavage fails, Polymerase dissociation fails, and some transcripts get far longer. The authors should demonstrate that eNSRA can detect this.<br>Response: We thank the reviewer for the great suggestion. In the revised manuscript, we demonstrate that eNRSA detects significant overall readthrough increase ($p<1.2e-12$) when termination factors (CPSF73 and CstF64+CstF64t) are depleted. This result has been included in the manuscript (Page 7 and Supplementary Fig. S1).<br><br>Minor Issues:<br>Figure 1 should include Peppro, Denr, Tfit and PINTS.<br>Response: We thank the reviewer for the suggestion. Peppro, Denr, Tfit, and PINTS have been included in Figure 1. We have also added brief introductions to these tools in the revised manuscript (Page 2).<br><br>1.Kaikkonen, M.U., et al., Control of VEGF-A transcriptional programs by pausing and genomic compartmentalization. Nucleic Acids Res, 2014. 42(20): p. 12570-84.<br>2.Yu, A.D. and M. Rosbash, Butt-seq: a new method for facile profiling of transcription. Genes Dev, 2023. 37(9-10): p. 432-448.<br>3.Nojima, T., et al., Mammalian NET-Seq Reveals Genome-wide Nascent Transcription Coupled to RNA Processing. Cell, 2015. 161(3): p. 526-540.<br>4.Gregersen, L.H., et al., SCAF4 and SCAF8, mRNA Anti-Terminator Proteins. Cell, 2019. 177(7): p. 1797-1813 e18.<br>5.Herzog, V.A., et al., Thiol-linked alkylation of RNA to assess expression dynamics. Nat Methods, 2017. 14(12): p. 1198-1204.<br>6.Schwalb, B., et al., TT-seq maps the human transient transcriptome. Science, 2016. 352(6290): p. 1225-8.<br>7.Lopez Martinez, D. and J.Q. Svejstrup, Mechanisms of RNA Polymerase II Termination at the 3'-End of Genes. J Mol Biol, 2025. 437(1): p. 168735.
**Additional Information:**	
**Question**	**Response**
Are you submitting this manuscript to a special series or article collection?	No

Powered by Editorial Manager® and ProduXion Manager® from Aries Systems Corporation

**Experimental design and statistics**<br><br>Full details of the experimental design and statistical methods used should be given in the Methods section, as detailed in our Minimum Standards Reporting Checklist. Information essential to interpreting the data presented should be made available in the figure legends.<br><br>Have you included all the information requested in your manuscript?	Yes
**Resources**<br><br>A description of all resources used, including antibodies, cell lines, animals and software tools, with enough information to allow them to be uniquely identified, should be included in the Methods section. Authors are strongly encouraged to cite Research Resource Identifiers (RRIDs) for antibodies, model organisms and tools, where possible.<br><br>Have you included the information requested as detailed in our Minimum Standards Reporting Checklist?	Yes
**Availability of data and materials**<br><br>All datasets and code on which the conclusions of the paper rely must be either included in your submission or deposited in publicly available repositories (where available and ethically appropriate), referencing such data using a unique identifier in the references and in the “Availability of Data and Materials” section of your manuscript.<br><br>Have you have met the above requirement as detailed in our Minimum Standards Reporting Checklist?	Yes

Powered by Editorial Manager® and ProduXion Manager® from Aries Systems Corporation

GigaScience has policies and guidelines in place for the use of generative AI-writing tools such as ChatGPT. If you have used such writing tools to assist with writing the manuscript this must be declared and cited in the text. Authors should not list AI-writing tools and other AI-assisted technologies as an author or co-author and should acknowledge that they are fully responsible for text generated or refined by AI-writing tools.<p><br><br>A summary of use (particularly in the introduction or among methods) needs to be included at the end of the paper, and the outputs should also be included as a supplementary file hosted in GigaDB or other open repositories. Please read our guidelines for more information. <p><br><br>By submitting to GigaScience, you are aware of the journal's AI-writing tools policy, and if you have declared use of such tools below, you have acknowledged this where appropriate in your manuscript and have made a summary of use and outputs available. <p><br><b>AI-assisted writing tools have been used in the preparation of this manuscript?	No

Manuscript

Click here to access/download;Manuscript;eNRSA-manuscript-revision-May 30.docx

eNRSA: A Faster and More Powerful Approach for Nascent Transcriptome Analysis

Jing Wang [1,2,†], Hua-chang Chen [1,2,†], Scott W. Hiebert [3,4], Quanhu Sheng [1,2], William P. Tansey [3,5], Yu Shyr [1,2,*], Qi Liu[1,2,*]

[1]Department of Biostatistics, Vanderbilt University School of Medicine, Nashville, TN, USA

[2]Center for Quantitative Sciences, Vanderbilt University Medical Center, Nashville, TN, USA

[3]Department of Biochemistry, Vanderbilt University School of Medicine, Nashville, TN, USA.

[4]Vanderbilt-Ingram Cancer Center, Nashville, TN, USA

[5] Department of Cell and Developmental Biology, Vanderbilt University School of Medicine, Nashville, TN, USA

† These authors contributed equally to this work

*** Correspondence**
Yu Shyr, yu.shyr@vumc.org; Qi Liu, qi.liu@vumc.org
Jing Wang [0000-0003-2775-9001]; Hua-chang Chen [0000-0003-0497-2483]; Scott W Hiebert [0000-0001-5621-1454]; Quanhu Sheng [0000-0001-8951-9295]; William P Tansey [0000-0002-3900-0978]; Yu Shyr [0000-0003-2086-967]; Qi Liu [0000-0001-8892-7078]

Abstract

Nascent RNA sequencing tracks primary transcriptional events, making it crucial for studying the immediate regulatory changes of genes and enhancers in response to both endogenous and exogenous stimuli. NRSA is a widely used tool for analyzing nascent transcriptomic data, enabling quantification of transcriptional changes at proximal promoters and gene bodies, estimation of pausing indices, identifying active enhancers, and establishing enhancer–target gene relationships. To improve its functionality and broaden its applicability to diverse organisms and complex study designs, we have developed an enhanced version, eNRSA. Key advancements include adaptive selection of major transcripts, support for any organism with known gene structures, compatibility with complex study designs, and identification of alternative transcription start and termination sites, as well as transcription readthrough events. Additionally, eNRSA achieves a ~20-fold increase in analysis speed while significantly reducing memory usage. These enhancements make eNRSA a faster, more versatile, and more powerful tool for

nascent transcriptome analysis. eNRSA is freely available at https://bioinfo.vanderbilt.edu/eNRSA/.

Keywords: nascent transcriptome analysis, adaptive major transcript, alternative transcription start site (ATSS), alternative transcription termination site (ATTS), transcription readthrough

Introduction

Transcription is a highly regulated process comprising multiple stages, each precisely controlled to ensure accurate gene expression [1-4]. These key transcriptional stages include initiation, pausing, elongation, and termination [4]. Unlike steady-state RNA sequencing, nascent RNA sequencing captures transcription that are actively synthesized, providing a direct measure of gene expression across various regulatory stages [5, 6]. This capability is particularly valuable for uncovering immediate, direct, and transient transcriptional changes that reflect cellular responses to diverse conditions, including stress, differentiation, or disease progression.

There are several sequencing-based techniques designed to extract newly transcribed RNAs from the total pool of cellular RNA. These include small capped RNA sequencing (Start-seq) [7], chromatin-associated RNA sequencing (caRNA-seq) [8, 9], global run-on sequencing (GRO-seq) [5], precision run-on sequencing (PRO-seq) [6], native elongating transcript sequencing (NET-seq) [10], mammalian NET-seq (mNET-seq) [11], thiol(SH)-linked alkylation for the metabolic sequencing of RNA (SLAM-seq) [12], bulk analysis of nascent transcript termini sequencing (Butt-seq) [13], and transient transcriptome sequencing (TT-seq) [14]. Of these, GRO-seq and PRO-seq are among the most widely used methods, largely due to their ability to provide high-resolution, genome-wide data on actively transcribing RNA polymerases. To analyze GRO/PRO-seq data, several tools have been developed, including dREG, FStitch, groHMM, Vespucci, nASAP, Tfit, NRSA, PEPPRO, DENR, and PINTS (Fig. 1). dREG [15, 16] and FStitch [17] focus on identifying active regulatory elements such as enhancers with divergent transcription. groHMM [18] quantifies nascent transcription for both known genes and enhancers, while Vespucci [19] also estimates transcriptional changes between conditions. nASAP [20] is a web server for nascent RNA analysis including transcription level quantification, pausing site identification and regulatory network construction. Tfit [21] aims to identify and profile bidirectional transcription sites using a finite mixture model. PEPPRO [22] is designed for quality control and preprocessing, and it also generates bigWig signal tracks for downstream analysis. DENR [23] models nascent reads as a mixture of user-provided isoforms, allowing it to estimate RNA abundance at both isoform and gene levels. PINTS [24] is a peak identifier that detects active promoters and enhancers genome-wide and pinpoints the precise location of 5' transcription start sites. Among these tools, NRSA [25]

stands out for its comprehensive analysis of nascent transcription. It not only quantifies nascent transcription and pausing for known genes, but also detects, annotates, quantifies, and prioritizes active enhancers (Fig. 1). However, NRSA is time- and memory-intensive, particularly when processing large-scale nascent transcriptomes. Additionally, its limitation to single-factor designs and the requirement to preprocess gene structure files by selecting one major transcript per gene diminishes its performance and restricts its applicability across diverse organisms and genomes.

Here, we developed eNRSA (enhanced NRSA) to significantly improve computational efficiency, broaden its applicability, and enhance performance (Fig. 1). eNRSA runs 20 times faster than NRSA while requiring only roughly 1/8 of the memory. Further, eNRSA enhances performance by adaptively selecting major transcripts based on the nascent transcriptome being analyzed and by supporting multi-factor experimental designs. These advancements make eNRSA applicable to any organism or genome with a known gene structure. By fully leveraging the unique characteristics of the nascent transcriptome, eNRSA introduces new functionalities to identify alternative transcription start sites (ATSS), alternative transcription termination sites (ATTS), and transcription readthrough (TRT) dysregulation across conditions. eNRSA is freely available at https://bioinfo.vanderbilt.edu/eNRSA/.

Results

Overview of eNRSA

Nascent RNA sequencing captures the production of newly synthesized RNAs, offering a comprehensive view of regulatory dynamics throughout the transcription cycle, including initiation, pausing, elongation, and termination [4, 26]. In 2018, we developed NRSA, which enables in-depth analysis of the nascent transcriptome at both gene and enhancer levels, surpassing other tools in scope and accuracy [25]. NRSA not only estimates promoter-proximal pausing and elongation rates, but also identifies, annotates, and quantifies active enhancers while measuring enhancer-mediated regulation.

To enhance NRSA’s performance and broaden its applications, we developed an advanced version, eNRSA, which supports any organism with a known gene structure, accommodates complex study designs, introduces new functions to identify ATSS, ATTS, and readthrough dysregulation, and significantly improves computational efficiency (Fig. 1). eNRSA takes nascent transcriptome data and a reference genome with a gene structure file as input, providing detailed outputs on transcriptional changes in promoter-proximal and gene body regions, alternative transcriptional events (including ATSS, ATTS, and readthrough), enhancer activity, and the pausing index, along with various visualization options (Fig. 2).

eNRSA introduces three key advancements: 1) adaptive selection of major transcripts, enabling data-driven definition of promoter-proximal and gene body regions, extending support to any organism with defined gene structures (compared to NRSA's limitation of five organisms and eight genomes); 2) advanced capabilities for complex study designs, improving differential transcriptional analysis by accounting for confounding factors and allowing for multifactorial designs; and 3) new functions to identify ATSS, ATTS, and readthrough dysregulation (Fig. 2). Additionally, eNRSA significantly optimizes computational performance by simultaneously increasing processing speed and reducing memory requirements.

eNRSA selects major transcripts adaptively and supports any organism with a known gene structure

One primary function of NRSA is to quantify transcriptional changes at promoter-proximal and gene body regions and to estimate the pausing index, which heavily depends on selecting the major transcript for each gene. Given that a single gene often encodes multiple transcripts, selecting the major transcript is crucial for accurately representing the gene's nascent transcription. This selection significantly impacts the definition of the corresponding promoter-proximal and gene body regions, ultimately influencing the accuracy of transcriptional regulation quantification. To simplify calculations, NRSA uses the longest transcript for each gene as the major transcript, defining promoter-proximal and gene body regions accordingly. Although NRSA preprocesses the gene structure file (GTF file) to extract the longest transcript for each gene from eight genomes across five organisms (hg19, hg38, mm10, mm39, dm3, dm6, ce10, and danRer10) and packs these preprocessed files in the tool for seamless analysis, users have to perform this preprocessing themselves if working with other organisms or different genome versions. This task is non-trivial and demands programming skills, limiting NRSA's broad application to various organisms. Furthermore, this approach may yield misleading findings if the longest transcript is not actually the major transcript for a given gene.

To address these issues, eNRSA automatically and adaptively selects major transcripts based on the nascent transcriptome data being analyzed. Unlike NRSA, which relies on preselected longest transcripts, eNRSA identifies transcripts with the highest nascent transcriptional levels as major transcripts. This data-driven approach is more accurate and flexible than a fixed selection method, as major transcripts may vary under different conditions. By removing the preprocessing step, eNRSA supports any organism with a known gene structure without additional procedures.

eNRSA improves differential transcription analysis by allowing complex study designs

Most nascent transcriptome analysis tools, including NRSA, are limited to handling simple study designs involving only two groups separated by a single factor of interest [19, 25],

lacking the ability to analyze complex study designs with multiple factors or confounding variables, such as batch effects. This limitation restricts NRSA's applicability and can result in unreliable and irreproducible findings. Failing to account for confounding factors can lead to either false differential transcription arising from technical rather than biological effects or loss of true differential signals.

For example, when NRSA was used to compare the nascent transcriptome between two conditions—DMSO-treated and dTAG47-treated cells for MYC binding depletion [27], it identified only 5 upregulated and 62 downregulated genes with an FDR<0.05 and $|\log_2 FoldChange|>0.3$, with no notable pathways emerging from functional enrichment analysis. Principal component analysis, however, revealed a strong batch effect, with DMSO-treated and dTAG47-treated cells from the same batch clustering even more closely than cells from the same condition in different batches (Fig. 3A). The lack of batch-effect correction likely explains why NRSA did not yield biologically meaningful results.

In contrast, eNRSA supports the analysis of complex study designs, with the ability to adjust for confounding factors, thereby enhancing differential transcription analysis. When applied to the same dataset of DMSO-treated and dTAG47-treated with the batch factor incorporated in the model, eNRSA identified 841 dysregulated genes with an FDR<0.05 and $|\log_2 FoldChange|>0.3$, including 287 upregulated and 554 downregulated ones (Fig. 3B). To further evaluate the performance of eNRSA, we permuted PRO-seq reads between DMSO and dTAG47-treated cells (100 permutations) to generate a background distribution, which was then used to estimate the probability of obtaining the observed difference by chance. All 841 genes were supported by the permutation-based test. Using this test as the gold standard, eNRSA achieved 80.1% sensitivity, 100% specificity, and 95.6% accuracy.

Notably, most downregulated genes in dTAG47-treated cells were either MYC targets or associated with core MYC functions. MYC is known to control the transcription of genes essential for ribosome biogenesis [28-32]. Consistently, eNRSA detected significant downregulation of ribosome-related genes in dTAG47-treated cells, including *RPLP2*, *RPL12*, *RPL13*, *RPL14*, *RPL32*, *RPL34*, *RPL35*, and *RPS24* (Fig. 3C; Supplementary Table 1). Functional enrichment analysis using WebGestalt [33] and GSEA [34] revealed significant downregulation of genes involved in ribosome biogenesis, mTORC1 signaling, and MYC targets (Normalized Enrichment Score (NES)= -3.07, FDR=0), as well as rRNA processing (NES=-3.05, FDR=0) (Figs. 3D and 3E; Supplementary Table 2). These findings demonstrate that eNRSA effectively unmasks true differential transcription signals previously obscured by batch effects, significantly enhancing detection power.

eNRSA identifies ATSS, ATTS and readthrough dysregulation

In mammalian genomes, most genes give rise to multiple transcript isoforms. At least 70% of genes have multiple polyadenylation sites, more than 50% have alternative

transcription start sites, and transcripts from nearly all genes can be subject to alternative splicing [35-38]. Differential transcript isoforms can encode products that differ in structure, location, stability, enzyme activity, and other properties, which regulate key biological processes and contribute to disease. Therefore, identifying these alternative events is crucial [35].

ATSS and ATTS have been reported to contribute more to isoform diversity than alternative splicing [35]. TSS denotes the annotated start of the gene, while TTS refers to the annotated end of the gene, defined as the 3′ end of the last exon in the GTF file, marking the cleavage and polyadenylation site of the mature RNA transcript. Nascent transcriptome data from GRO/PRO-seq, (m)NET-seq, and Butt-seq, are characterized by peaks at promoter-proximal regions and an accumulation of reads around TTS sites, providing a natural way to identify ATSS and ATTS. However, no tools are currently available to specifically identify ATSS and ATTS from nascent transcriptome data. Leveraging these unique characteristics, eNRSA identifies ATSS by detecting shifts in read distributions across promoter-proximal regions, and ATTS by detecting shifts across TTS sites, between two conditions. Each promoter-proximal region corresponds to a TSS site, where higher read counts indicate greater TSS usage. A shift in read enrichment from one TSS site to another signals an ATSS event, which eNRSA assesses using the Cochran–Mantel–Haenszel test (CMH) for statistical significance (details in Methods). Similarly, eNRSA detects ATTS events by counting and estimating shifts in read distributions across TTS sites.

eNRSA demonstrated strong performance in detecting ATSS and ATTS events, with findings further supported by RNA-seq data. When applied to compare nascent transcriptome between two cell lines, Ramos and G401, eNRSA identified 1,405 ATSSs and 905 ATTSs at an FDR < 0.05 (Supplementary Tables 3 & 4). These events were then compared to those detected from RNA-seq data. Given the complexity of transcriptional regulation and the fact that nascent transcriptome profiling and RNA-seq capture different layers of transcription, a strong overlap was observed between the two datasets. Specifically, among the 1,405 ATSSs, 1,054 (75.0%) were also supported by RNA-seq; likewise, among the 905 ATTSs, 736 (81.3%) were confirmed by RNA-seq (Fig. 4A; Supplementary Tables 3 & 4). As an illustrative example, the *SCP2* gene exhibited one of the most significant ATSS event (FDR=0). In the G401 cell line, promoter-proximal peaks were observed at two TSS sites: one at chr1:53,392,901 (hg19) corresponding to the long transcript NM_001193599.2, and the other at chr1:53,480,610 (hg19) corresponding to the short transcript NM_001007250.2. In contrast, only a single peak was observed at the second TSS site (chr1:53,480,610) in the Ramos cell line (Fig. 4B). This shift in read enrichment—from two TSS sites in G401 to one in Ramos—clearly indicates an ATSS event. This event was validated by RNA-seq data from the same cell lines, where both long and short transcripts were detected in G401, but only the short transcript was present

in Ramos (Fig. 4B). The differential TSS usage between these cell lines suggests that *SCP2* may produce distinct proteins with differing functions. According to Uniprot, the two *SCP2* transcripts encode distinct proteins through transcription initiation from independently regulated promoters. The long transcript encodes SCPx, a thiolase enzyme essential for peroxisomal oxidation of branched-chain fatty acids [39], while the short transcript encodes SCP2, an intracellular lipid transfer protein that facilitates the transfer of common phospholipids, cholesterol, and gangliosides from the endoplasmic reticulum to the plasma membrane [40-42].

From a Polymerase II (Pol II) perspective, transcription termination does not occur immediately at the cleavage site. Instead, Pol II continues transcribing downstream, and dissociation typically occurs 1–50 kb downstream [14, 43, 44]. Therefore, eNRSA defines “readthrough” as transcriptionally engaged Pol II detected downstream of the annotated TTS/cleavage site and extending to the Pol II dissociation zone. This readthrough signal is expected in nascent transcription assays such as PRO-seq, GRO-seq, NET-seq, mNET-seq, Butt-seq, which capture engaged polymerase II, or TT-seq, which also maps transient RNA downstream of polyadenylation sites [14]. In contrast, assays like SLAM-seq, which detect metabolically labeled RNAs post-transcriptionally, are less likely to show signal downstream of the cleavage site due to rapid degradation of unprocessed or unstable transcripts in the readthrough region [12]. Transcription readthrough is observed not only in various cellular stress conditions but also in healthy tissues, suggesting that readthrough transcripts may play a role in regulating cellular processes [45, 46]. To detect readthrough dysregulation, eNRSA calculates a readthrough ratio by comparing the number of reads within a fixed 50 kb downstream of the TTS relative to the number of reads in the terminal exon [43], then estimates the ratio change between two conditions to identify dysregulations (details in Methods). Using this approach, eNRSA identified 217 protein-coding genes with reduced readthrough, and 160 with increased readthrough, in engineered G401 cells expressed OmoMYC—a dominant-negative mutant that blocks the productive association of MYC with its target genes, compared to cells expressing inducible forms of enhanced green fluorescent protein (EGFP, control) (FDR < 0.05; Supplementary Table 5). Metagene analysis of nascent transcription for genes with increased readthrough in OmoMYC G401 cells revealed consistent read accumulation beyond the TTS, whereas genes with decreased readthrough showed read depletion in this region (Fig. 4C).

Cleavage and polyadenylation (CPA) factors, including CPSF73, CstF64, and CstF64t, play crucial roles in the 3' end processing of RNA and are essential for proper transcription termination. A previous study using mNET-seq profiling has shown that depletion of CPSF73 and the double knockdown of CstF64 and CstF64t proteins lead to termination defects [11]. When applied to the mNET-seq data, eNRSA revealed significant overall

increases in readthrough (Supplementary Fig. S1, $p < 1.2e-12$), indicating termination defects.

eNRSA significantly increases speed and decreases memory usage

Although NRSA has been widely used for nascent transcriptome data analysis [47-53], it struggles to handle large datasets efficiently, requiring long runtime and high memory usage. eNRSA improves both speed and memory efficiency by implementing Python in place of R, using novel algorithms, and adopting a streaming process. In simulations with increasing nascent transcriptome sizes, eNRSA reduced computational time by over 20-fold compared to NRSA. For example, NRSA took 1.46 hours to process 30.3 million nascent RNA reads, while eNRSA required only 0.07 hours. With 387.9 million reads, NRSA took 10.51 hours, whereas eNRSA completed the task in just 0.51 hours (Fig. 5A). Additionally, eNRSA significantly lowered memory demands. While NRSA's memory usage scaled linearly with read counts (from 10.96 GB for 30.3M reads to 139.2 GB for 387.9M reads), eNRSA maintained a constant 4.78 GB memory requirement regardless of read counts (Fig. 5B). These results highlight eNRSA's substantial computational efficiency over NRSA, making it a valuable tool for analyzing large datasets.

Since eNRSA automatically selects the major transcript for each gene from the gene structure file, we further evaluated its computational efficiency with respect to the number of transcripts defined in the GTF file. As a result, eNRSA showed only a minimal increase in runtime and consistent memory usage as the number of transcripts in the GTF file grew from 40.3K (RefGene) to 163.7K (EnsGene) (Figs. 5C & 5D).

Discussion

eNRSA offers a powerful and scalable solution for nascent transcriptome analysis, addressing the limitations of NRSA while meeting the increasing demands of analyzing diverse organisms and complex study designs. By incorporating adaptive transcript selection, supporting multifactor designs, and introducing new capabilities to identify ATSS, ATTS, and transcription readthrough, eNRSA significantly enhances performance and broadens its applicability. Combined with substantial improvements in computational efficiency, eNRSA stands out as a scalable, efficient, and versatile tool for gaining deeper insights into transcriptional regulation across multiple stages. Although eNRSA is primarily designed to analyze GRO-seq or PRO-seq data, it can also be applied to data generated by other nascent RNA sequencing technologies that capture transcriptional dynamics, including pausing, elongation, and termination, such as Butt-seq, NET-seq, and mNET-seq. Examples of these applications are provided in Supplementary Table S6, Figs. S1 and S2, where the results are consistent with the findings reported in the original publications [11, 13, 54]. For nascent RNA sequencing technologies that capture only

specific aspects of transcriptional dynamics, eNRSA results should be interpreted with caution. For instance, SLAM-seq tracks RNA kinetics including transcription, processing and degradation, and metabolically labeled RNAs are subject to degradation over time [12]. Therefore, while transcriptional changes within the gene body identified by eNRSA may still be meaningful, results related to pausing, ATSS/ATTS, and readthrough may not be interpretable—either because SLAM-seq does not capture pausing dynamics or due to RNA degradation leading to limited coverage beyond transcription end sites. In contrast, TT-seq maps the entire range of transient RNA and monitors RNA synthesis and degradation, which doesn't yield peak signals near the promoter where polymerase pauses like GRO/PRO-seq, NET-seq, Butt-seq, or mNET-seq [14]. Therefore, eNRSA results on gene body, ATTS, and readthrough may be informative, but those related to pausing may not be applicable.

To further advance the study of transcriptional regulation, eNRSA could be enhanced by integrating data from chromatin accessibility or binding assays, such as ATAC-seq or ChIP-seq. This integration would enable the linking of pausing behavior with chromatin state changes, transcription factor binding, and enhancer-promoter interactions, offering deeper insights into the regulatory landscape underlying transcription initiation, pausing, elongation, and termination. Additionally, eNRSA could be extended to analyze single-cell nascent transcriptome, such as scGRO-seq [55], unlocking new opportunities to study immediate, cell-type-specific transcriptional changes and enabling a more detailed investigation of heterogeneity in gene expression across different cell types and conditions.

eNRSA relies heavily on predefined gene structures (e.g., from GTF files) to define gene boundaries for transcriptional quantification. While this approach is efficient, it may limit the tool's ability to detect novel or poorly annotated genes, especially in regions with uncharacterized or alternative gene models. Implementing more flexible annotation systems or incorporating RNA-Seq data could help address this limitation. eNRSA identifies ATSS and ATTS by detecting distinct promoter-proximal pausing and cleavage/polyadenylation signals. However, nascent transcription data has limited resolution when it comes to accurately defining alternative TSS and TTS, particularly in regions with overlapping or closely spaced initiation/termination sites. This limits eNRSA's ability to fully capture all potential ATSS and ATTS events, especially in genes with complex promoter architectures or multiple isoforms. eNRSA uses read counts mapped to promoter-proximal and CPS regions to detect ATSS and ATTS, but transcriptional noise from neighboring genes can complicate the identification process. While eNRSA attempts to mitigate this noise by excluding closely located genes, this strategy may not always be sufficient, particularly when genes are densely packed or transcriptionally active in close proximity. Furthermore, eNRSA assumes that differences in promoter-proximal pausing regions suggest alternative TSS usage, but such differences can also arise from post-

initiation regulatory mechanisms. To confidently link pausing behavior to TSS heterogeneity, it is essential to integrate complementary data types. For example, combining PRO-seq data with TSS-specific methods like CAGE-seq can help validate TSS locations. Additionally, RNA-seq data can assess differential isoform expression, which may align with alternative TSS usage. Mapping histone modifications, such as H3K4me3 (a mark of active promoters), can also provide insights into how pausing regions correlate with distinct TSSs.

Methods

Adaptive selection of major transcripts

Promoter-proximal and gene body regions are determined based on TSS and TTS sites. The promoter-proximal region is defined by examining each 50 bp window with a 5 bp sliding step along the coding strand, spanning ±500 bp from the TSS. The 50 bp window with the highest number of reads is selected as the promoter-proximal region. The gene body is defined as the region extending from +1 kb downstream of the TSS to the TTS [25].

Unlike NRSA, which preselects the longest transcript of each gene as the major transcript, eNRSA adopts a data-driven approach to determine the major transcript based on the nascent transcriptome data being analyzed. For each gene, eNRSA groups transcripts with identical TSS and TTS sites, quantifies transcription activity in the promoter-proximal and gene body regions for each group, and selects the transcript group with the highest reads in the promoter-proximal region as the major transcript. If two groups have the same number of promoter-proximal reads (i.e., share the same TSS), the transcript group with the highest number of reads in the gene body region is selected.

Differential transcription analysis in complex study designs

After quantifying reads in the promoter-proximal and gene body regions, eNRSA estimates transcriptional alterations in both regions between two conditions. To facilitate differential expression analysis and accommodate complex study designs, eNRSA integrates PyDEseq2, a Python implementation of the DESeq2 workflow for differential expression analysis. Users provide an experimental design file that includes a column specifying the path to each sample's alignment file, and additional columns indicating each sample’s group assignments. Each sample can belong to multiple groups. eNRSA uses this design file to build a DESeq2 [56] model for differential comparisons.

To detect and quantify intergenic enhancers, eNRSA utilizes the HOMER package (http://homer.salk.edu/) to call novel transcripts using default parameters (tssFold > 4 and bodyFold > 3) on reads pooled from all samples. Transcripts located within −2 kb to +20

kb of any annotated gene are excluded. Active enhancers are then defined as regions exhibiting pairs of bidirectional transcripts, and their activity is quantified by the number of reads mapped to the corresponding enhancer region [25].

Notably, eNRSA allows users to provide their own normalization factors, such as those derived from spike-ins. If no normalization factor is provided, eNRSA applies the default DESeq2 normalization method to gene body expression and then uses the same normalization factor to normalize transcription in the promoter-proximal regions and enhancers.

ATSS and ATTS identification

Nascent transcription sequencing captures promoter-proximal pausing, a regulatory step where RNA polymerase II (Pol II) pauses after initiating transcription, typically 20–60 nucleotides downstream of the TSS. Distinct clusters of paused Pol II near a gene's promoter, referred to as distinct pausing regions, may indicate alternative TSS usage. To identify potential alternative TSSs (ATSSs), eNRSA compares read counts within two promoter-proximal pausing regions under different conditions and evaluates the association between TSS usage and condition. For each gene, all TSS sites are considered, with pairwise comparisons restricted to TSSs separated by at least 1000 bp to avoid mixed signals and enhance reliability. For example, if a gene has three TSSs (TSS1, TSS2, TSS3), eNRSA evaluates usage changes between TSS1 and TSS2, TSS1 and TSS3, and TSS2 and TSS3, while excluding closely spaced TSSs (<1000 bp apart). Changes in TSS usage between conditions are assessed by constructing 2×2 contingency tables for each pair of samples, one from each condition, based on read counts corresponding to the two TSSs being compared. Rather than pooling data across samples, which may obscure biological variability, the Cochran-Mantel-Haenszel (CMH) test is used to perform a stratified analysis. This test evaluates whether there is a consistent association between TSS usage and condition across sample pairs, while controlling for sample-specific variability. Under the assumption of a common odds ratio across strata, the CMH test increases statistical power and reduces bias due to heterogeneity among samples. This framework ensures robust detection of systematic shifts in TSS usage across biological replicates.

Similarly, nascent transcription accumulates at cleavage and polyadenylation sites, where distinct clusters of reads near a gene's termination may indicate alternative TTS usage. To detect ATTS, eNRSA compares read counts mapped between -1 kb and +2 kb of two TTS sites and assesses the association between TTS usage and the condition using a CMH test, analogous to the ATSS analysis. For each gene, all TTS sites were considered. To minimize transcriptional noise from neighboring genes, eNRSA excludes any TTS site that overlaps with another gene or has a gene located within 3 kb downstream.

Readthrough dysregulation

Nascent transcription sequencing measures RNA still associated with actively transcribing RNA polymerase, enabling it to capture readthrough reads, which are transcripts extending beyond the normal termination site. eNRSA quantifies transcriptional readthrough using the ratio of the number of reads mapped within a fixed 50 kb region downstream of the TTS to the number of reads mapped within the last exon [43]. A significant change in this ratio under different conditions indicates readthrough dysregulation. To ensure reliability, eNRSA considers only active genes, defined as those with promoter-proximal read densities greater than zero and gene-body densities exceeding four reads per kilobase after total read counts are normalized to 10 million based on background estimation. The significance of changes in the readthrough ratio is assessed using the CMH test applied in a manner similar to the TSS analysis. To minimize transcriptional noise from neighboring genes, eNRSA excludes genes that have other active genes within 50 kb downstream.

Running speed and memory improvements

To improve speed and reduce memory usage, eNRSA adopts an optimized strategy for transcriptional quantification. While NRSA uses a gene-centered approach that scans the nascent transcriptome data repeatedly to count reads mapped to each gene, eNRSA takes a more efficient approach. eNRSA begins by performing two types of read counting: one at the individual site-specific level and another by summing reads within 200 bp binned regions. These counts are then used to generate a comprehensive count matrix. The count matrix, created for each chromosome, is stored in a python dictionary and saved as a binary pickle file for downstream analysis. For each gene defined in the GTF file, eNRSA consolidates transcripts with identical TSS and TTS into a single entity. The transcript group's region are then overlapped with the precomputed count matrix for quantification. This strategy allows eNRSA to process the alignment data only once, significantly enhancing efficiency. Additionally, it groups the transcripts by chromosome, loading only the corresponding count matrix for the active chromosome. Once all transcripts for that chromosome are processed, the memory is released, further optimizing resource usage. By replacing all R-based coeds in NRSA with Python, eNRSA achieves faster runtimes, making it suitable for large-scale nascent transcriptome studies.

eNRSA installation and implementation

eNRSA is implemented in Python 3 (> = 3.6) and the dependencies include BEDTools, HOMER, and two Python packages - PyDESeq2 and Fisher. eNRSA can be executed in a standard Python environment with the required dependencies installed either by conda or Docker container. A Docker image can be found at Docker Hub (https://hub.docker.com/repository/docker/chccode/enrsa/). eNRSA, along with its detailed manual, including installation instructions, implementation guidelines, and output descriptions, are available at https://bioinfo.vanderbilt.edu/eNRSA/.

Other bioinformatics analysis

To detect ATSSs and ATTSs from RNA-seq data, we calculated the ratio of reads mapped to the two alternative TSS or TTS sites for each sample pair, with one sample from each condition. To assess the significance of ATSS and ATTS events across sample pairs between the two conditions, we applied the Cochran–Mantel–Haenszel (CMH) test.

The PCA plot, volcano plot and the performance bar plots were generated in R using ggplot2 package [57]. Functional enrichment analysis was performed by WebGestalt 2024 and GSEA_4.3.3 [33, 34]. The snapshots were from IGV_2.11.0 [58]. The PRO-seq profiles for readthrough disruption were generated by deepTools_3.5.6 with the last exon scaled to 1000bp [59].

Nascent transcriptomic datasets

The PRO-seq data for DMSO-treated and dTAG47-treated cells in the G401 cell line are available at the Gene Expression Omnibus (GEO) under accession number GSE164926. The PRO-seq data for the G401 and Ramos cell lines can be accessed from GEO under accession numbers GSE173207 and GSE183781, respectively. RNA-seq data for the G401 and Ramos cell lines are available from GEO under accession numbers GSE173207 and GSE212456. The PRO-seq data for G401 cells expressing EGFP control and OmoMYC are available under accession number GSE109310. Additionally, the GRO-seq data from VEGF-A stimulated and non-stimulated HUVEC cells, Butt-seq data from KL1-treated and DMSO-treated S2 cells, and S2P mNET-seq data from siCPSF73-treated, siCstF64+siCstF64t-treated, and control siRNA-treated HeLa cells are available at GSE52642, GSE228595 and GSE60358, respectively.

Availability of supporting source code and requirements

Project name: eNRSA

Project home page: https://bioinfo.vanderbilt.edu/eNRSA/

Operating system(s): Platform independent

Programming language: Python

Other requirements: Python3.8 or higher, HOMERs v5.1, bedtools v2.31.0

License: GNU GPL-3.0.

biotoolsID: biotools:enrsa; https://bio.tools/enrsa

RRID: SCR_026814

Data availability

A version of record snapshot of the GitHub repository has been archived in the Software Heritage [60].

List of abbreviations

ATSS: alternative transcription start sites

ATTS: alternative transcription termination sites

Butt-seq: bulk analysis of nascent transcript termini sequencing

caRNA-seq: chromatin-associated RNA sequencing

CMH: Cochran–Mantel–Haenszel test

CPS: cleavage and polyadenylation sites

EGFP: enhanced green fluorescent protein

eNRSA: enhanced NRSA

GEO: Gene Expression Omnibus

GRO-seq: global run-on sequencing

GTF: gene structure file

mNET-seq: mammalian NET-seq

NES: normalized enrichment score

NET-seq: native elongating transcript sequencing

NRSA: nascent RNA sequencing analysis

Pol II: RNA polymerase II

PRO-seq: precision run-on sequencing

SLAM-seq: thiol(SH)-linked alkylation for the metabolic sequencing of RNA

Start-seq: small capped RNA sequencing

TT-seq: transient transcriptome sequencing

Competing interests

The authors declare that they have no competing interests.

Funding

This work is supported by National Cancer Institute grants (P01 CA229123, U54 CA274367, R01 CA200709, and R01 CA247833), National Institutes of Health (P01 AI139449), Cancer Center Support Grant (P30 CA068485), 2024/2025 Biostatistics Faculty Development Award from Department of Biostatistics in VUMC.

Authors' contributions

Jing Wang: Data curation, Conceptualization, Formal analysis, Methodology, Supervision, Writing – original draft, Writing – review & editing, Funding acquisition. Hua-chang Chen: Writing – original draft, Software, Resources, Methodology, Formal analysis, Data curation. Scott W. Hibert: Writing – review & editing, Investigation. Quanhu Sheng: Software, Writing – review & editing. William P. Tansey: Writing – review & editing, Investigation, Funding acquisition. Yu Shyr: Writing – review & editing, Investigation. Qi Liu: Writing – review & editing, Supervision, Methodology, Investigation, Project administration, Funding acquisition, Conceptualization.

References

1. Tian, B. and J.H. Graber, *Signals for pre-mRNA cleavage and polyadenylation.* Wiley Interdiscip Rev RNA, 2012. **3**(3): p. 385-96.
2. Fuda, N.J., M.B. Ardehali, and J.T. Lis, *Defining mechanisms that regulate RNA polymerase II transcription in vivo.* Nature, 2009. **461**(7261): p. 186-92.
3. Vihervaara, A., F.M. Duarte, and J.T. Lis, *Molecular mechanisms driving transcriptional stress responses.* Nat Rev Genet, 2018. **19**(6): p. 385-397.
4. Wissink, E.M., et al., *Nascent RNA analyses: tracking transcription and its regulation.* Nat Rev Genet, 2019. **20**(12): p. 705-723.
5. Core, L.J., J.J. Waterfall, and J.T. Lis, *Nascent RNA sequencing reveals widespread pausing and divergent initiation at human promoters.* Science, 2008. **322**(5909): p. 1845-8.
6. Kwak, H., et al., *Precise maps of RNA polymerase reveal how promoters direct initiation and pausing.* Science, 2013. **339**(6122): p. 950-3.
7. Nechaev, S., et al., *Global analysis of short RNAs reveals widespread promoter-proximal stalling and arrest of Pol II in Drosophila.* Science, 2010. **327**(5963): p. 335-8.
8. Bhatt, D.M., et al., *Transcript dynamics of proinflammatory genes revealed by sequence analysis of subcellular RNA fractions.* Cell, 2012. **150**(2): p. 279-90.
9. Pandya-Jones, A., et al., *Splicing kinetics and transcript release from the chromatin compartment limit the rate of Lipid A-induced gene expression.* RNA, 2013. **19**(6): p. 811-27.
10. Churchman, L.S. and J.S. Weissman, *Nascent transcript sequencing visualizes transcription at nucleotide resolution.* Nature, 2011. **469**(7330): p. 368-73.

11. Nojima, T., et al., *Mammalian NET-Seq Reveals Genome-wide Nascent Transcription Coupled to RNA Processing.* Cell, 2015. **161**(3): p. 526-540.
12. Herzog, V.A., et al., *Thiol-linked alkylation of RNA to assess expression dynamics.* Nat Methods, 2017. **14**(12): p. 1198-1204.
13. Yu, A.D. and M. Rosbash, *Butt-seq: a new method for facile profiling of transcription.* Genes Dev, 2023. **37**(9-10): p. 432-448.
14. Schwalb, B., et al., *TT-seq maps the human transient transcriptome.* Science, 2016. **352**(6290): p. 1225-8.
15. Danko, C.G., et al., *Identification of active transcriptional regulatory elements from GRO-seq data.* Nat Methods, 2015. **12**(5): p. 433-8.
16. Wang, Z., et al., *Identification of regulatory elements from nascent transcription using dREG.* Genome Res, 2019. **29**(2): p. 293-303.
17. Azofeifa, J.G., et al., *An Annotation Agnostic Algorithm for Detecting Nascent RNA Transcripts in GRO-Seq.* IEEE/ACM Trans Comput Biol Bioinform, 2017. **14**(5): p. 1070-1081.
18. Chae, M., C.G. Danko, and W.L. Kraus, *groHMM: a computational tool for identifying unannotated and cell type-specific transcription units from global run-on sequencing data.* BMC Bioinformatics, 2015. **16**: p. 222.
19. Allison, K.A., et al., *Vespucci: a system for building annotated databases of nascent transcripts.* Nucleic Acids Res, 2014. **42**(4): p. 2433-47.
20. Wang, Z., et al., *nASAP: A Nascent RNA Profiling Data Analysis Platform.* J Mol Biol, 2023. **435**(14): p. 168142.
21. Azofeifa, J.G. and R.D. Dowell, *A generative model for the behavior of RNA polymerase.* Bioinformatics, 2017. **33**(2): p. 227-234.
22. Smith, J.P., et al., *PEPPRO: quality control and processing of nascent RNA profiling data.* Genome Biol, 2021. **22**(1): p. 155.
23. Zhao, Y., et al., *Deconvolution of expression for nascent RNA-sequencing data (DENR) highlights pre-RNA isoform diversity in human cells.* Bioinformatics, 2021. **37**(24): p. 4727-4736.
24. Yao, L., et al., *A comparison of experimental assays and analytical methods for genome-wide identification of active enhancers.* Nat Biotechnol, 2022. **40**(7): p. 1056-1065.
25. Wang, J., et al., *Nascent RNA sequencing analysis provides insights into enhancer-mediated gene regulation.* BMC Genomics, 2018. **19**(1): p. 633.
26. Henninger, J.E. and R.A. Young, *An RNA-centric view of transcription and genome organization.* Mol Cell, 2024. **84**(19): p. 3627-3643.
27. Woodley, C.M., et al., *Multiple interactions of the oncoprotein transcription factor MYC with the SWI/SNF chromatin remodeler.* Oncogene, 2021. **40**(20): p. 3593-3609.
28. van Riggelen, J., A. Yetil, and D.W. Felsher, *MYC as a regulator of ribosome biogenesis and protein synthesis.* Nat Rev Cancer, 2010. **10**(4): p. 301-9.
29. Ramalho, S., A. Dopler, and W.J. Faller, *Ribosome specialization in cancer: a spotlight on ribosomal proteins.* NAR Cancer, 2024. **6**(3): p. zcae029.
30. Zacarias-Fluck, M.F., L. Soucek, and J.R. Whitfield, *MYC: there is more to it than cancer.* Front Cell Dev Biol, 2024. **12**: p. 1342872.

31. Wolpaw, A.J., et al., *Drugging the "Undruggable" MYCN Oncogenic Transcription Factor: Overcoming Previous Obstacles to Impact Childhood Cancers.* Cancer Res, 2021. **81**(7): p. 1627-1632.
32. Popay, T.M., et al., *MYC regulates ribosome biogenesis and mitochondrial gene expression programs through its interaction with host cell factor-1.* Elife, 2021. **10**. DOI: 10.7554/eLife.60191
33. Elizarraras, J.M., et al., *WebGestalt 2024: faster gene set analysis and new support for metabolomics and multi-omics.* Nucleic Acids Res, 2024. **52**(W1): p. W415-W421.
34. Subramanian, A., et al., *Gene set enrichment analysis: a knowledge-based approach for interpreting genome-wide expression profiles.* Proc Natl Acad Sci U S A, 2005. **102**(43): p. 15545-50.
35. Reyes, A. and W. Huber, *Alternative start and termination sites of transcription drive most transcript isoform differences across human tissues.* Nucleic Acids Res, 2018. **46**(2): p. 582-592.
36. Pan, Q., et al., *Deep surveying of alternative splicing complexity in the human transcriptome by high-throughput sequencing.* Nat Genet, 2008. **40**(12): p. 1413-5.
37. Carninci, P., et al., *Genome-wide analysis of mammalian promoter architecture and evolution.* Nat Genet, 2006. **38**(6): p. 626-35.
38. Tian, B. and J.L. Manley, *Alternative polyadenylation of mRNA precursors.* Nat Rev Mol Cell Biol, 2017. **18**(1): p. 18-30.
39. Ferdinandusse, S., et al., *Peroxisomal fatty acid oxidation disorders and 58 kDa sterol carrier protein X (SCPx). Activity measurements in liver and fibroblasts using a newly developed method.* J Lipid Res, 2000. **41**(3): p. 336-42.
40. Stanley, W.A., et al., *Recognition of a functional peroxisome type 1 target by the dynamic import receptor pex5p.* Mol Cell, 2006. **24**(5): p. 653-663.
41. Puglielli, L., et al., *Sterol carrier protein-2 is involved in cholesterol transfer from the endoplasmic reticulum to the plasma membrane in human fibroblasts.* J Biol Chem, 1995. **270**(32): p. 18723-6.
42. Seedorf, U., et al., *Structure-activity studies of human sterol carrier protein 2.* J Biol Chem, 1994. **269**(4): p. 2613-8.
43. Gregersen, L.H., et al., *SCAF4 and SCAF8, mRNA Anti-Terminator Proteins.* Cell, 2019. **177**(7): p. 1797-1813 e18.
44. Lopez Martinez, D. and J.Q. Svejstrup, *Mechanisms of RNA Polymerase II Termination at the 3'-End of Genes.* J Mol Biol, 2025. **437**(1): p. 168735.
45. Caldas, P., et al., *Transcription readthrough is prevalent in healthy human tissues and associated with inherent genomic features.* Commun Biol, 2024. **7**(1): p. 100.
46. Papadopoulos, D., et al., *MYCN recruits the nuclear exosome complex to RNA polymerase II to prevent transcription-replication conflicts.* Mol Cell, 2022. **82**(1): p. 159-176 e12.
47. Taylor, S.J., et al., *Pharmacological restriction of genomic binding sites redirects PU.1 pioneer transcription factor activity.* Nat Genet, 2024. **56**(10): p. 2213-2227.
48. Layden, H.M., et al., *Mutant FOXO1 controls an oncogenic network via enhancer accessibility.* Cell Genom, 2024. **4**(4): p. 100537.

49. Kelly, R.D.W., et al., *Histone deacetylases maintain expression of the pluripotent gene network via recruitment of RNA polymerase II to coding and noncoding loci.* Genome Res, 2024. **34**(1): p. 34-46.
50. Zhao, J., et al., *Inherited blood cancer predisposition through altered transcription elongation.* Cell, 2024. **187**(3): p. 642-658 e19.
51. Bressin, A., et al., *High-sensitive nascent transcript sequencing reveals BRD4-specific control of widespread enhancer and target gene transcription.* Nat Commun, 2023. **14**(1): p. 4971.
52. Bomber, M.L., et al., *Human SMARCA5 is continuously required to maintain nucleosome spacing.* Mol Cell, 2023. **83**(4): p. 507-522 e6.
53. Zhang, S., et al., *PAX3-FOXO1 coordinates enhancer architecture, eRNA transcription, and RNA polymerase pause release at select gene targets.* Mol Cell, 2022. **82**(23): p. 4428-4442 e7.
54. Kaikkonen, M.U., et al., *Control of VEGF-A transcriptional programs by pausing and genomic compartmentalization.* Nucleic Acids Res, 2014. **42**(20): p. 12570-84.
55. Mahat, D.B., et al., *Single-cell nascent RNA sequencing unveils coordinated global transcription.* Nature, 2024. **631**(8019): p. 216-223.
56. Love, M.I., W. Huber, and S. Anders, *Moderated estimation of fold change and dispersion for RNA-seq data with DESeq2.* Genome Biol, 2014. **15**(12): p. 550.
57. Villanueva, R.A.M. and Z.J. Chen, *ggplot2: Elegant Graphics for Data Analysis, 2nd edition.* Measurement-Interdisciplinary Research and Perspectives, 2019. **17**(3): p. 160-167.
58. Robinson, J.T., et al., *Integrative genomics viewer.* Nat Biotechnol, 2011. **29**(1): p. 24-6.
59. Ramirez, F., et al., *deepTools2: a next generation web server for deep-sequencing data analysis.* Nucleic Acids Res, 2016. **44**(W1): p. W160-5.
60. Jing Wang, Hua-chang Chen, Scott W. Hiebert, Quanhu Sheng, William P. Tansey, Yu Shyr, Qi Liu (2025) eNRSA: A Faster and More Powerful Approach for Nascent Transcriptome Analysis (Version 1). [Computer software]. Software Heritage,2025. https://archive.softwareheritage.org/swh:1:snp:c5562ccec07e41e203a75d5f6a31d3a8d52943a5;origin=https://github.com/chc-code/eNRSA.git.

Figures

Figure 1. Summary of features distinguishing eNRSA from exiting nascent RNA sequencing analysis tools.

Figure 2. Workflow of eNRSA.

Figure 3. eNRSA enhances differential analysis by removing the batch effect. (A) PCA plot of normalized counts mapped to gene body for DMSO and dTAG47-treated cells from two batches. (B) The number of dysregulated genes identified by NRSA and eNRSA with an FDR<0.05. (C) Volcano plot showing the $\log_2$ fold change (x-axis) and $-\log_{10}$ FDR (y-axis) for dTAG47-treated vs. DMSO-treated on gene body transcription. (D) Pathways

enriched in the downregulated genes in dTAG47-treated vs. DMSO-treated cells by WebGestalt 2024. (E) GSEA results revealing the two most significant pathways, MYC targets and rRNA processing, enriched in the downregulation of dTAG47-treated cells compared to DMSO.

Figure 4. Example of ATSS and readthrough dysregulation identified by eNRSA. (A) ATSSs (up) and ATTSs (bottom) identified from PRO-seq and RNA-seq between G401 and Ramos cells. (B) IGV screenshot of PRO-seq and RNA-seq signals illustrating ATSS events between G401 and Ramos cells. In the G401 cell line, promoter-proximal peaks were observed at two TSS sites, while only one single peak was observed at the second TSS site in the Ramos cell line. (C) Metagene profiles of PRO-seq data for the genes with increased (left) and decreased (right) readthrough in OmoMYC vs. EGFP.

Figure 5. Performance comparison between eNRSA and NRSA. (A) Runtime with increasing numbers of PRO-seq reads. (B) Memory usage with increasing numbers of PRO-seq reads. (C) Runtime with increasing numbers of transcripts defined in the GTF file. (D) Memory usage with increasing numbers of transcripts defined in the GTF file. Computations were performed using a single thread of an Intel Xeon E5-2695 v4 @ 2.10GHz processor with 1T memory.

Figures revision
Click here to access/download;Figure;eNRSA-Figures-revision.pdf

Fig. 1

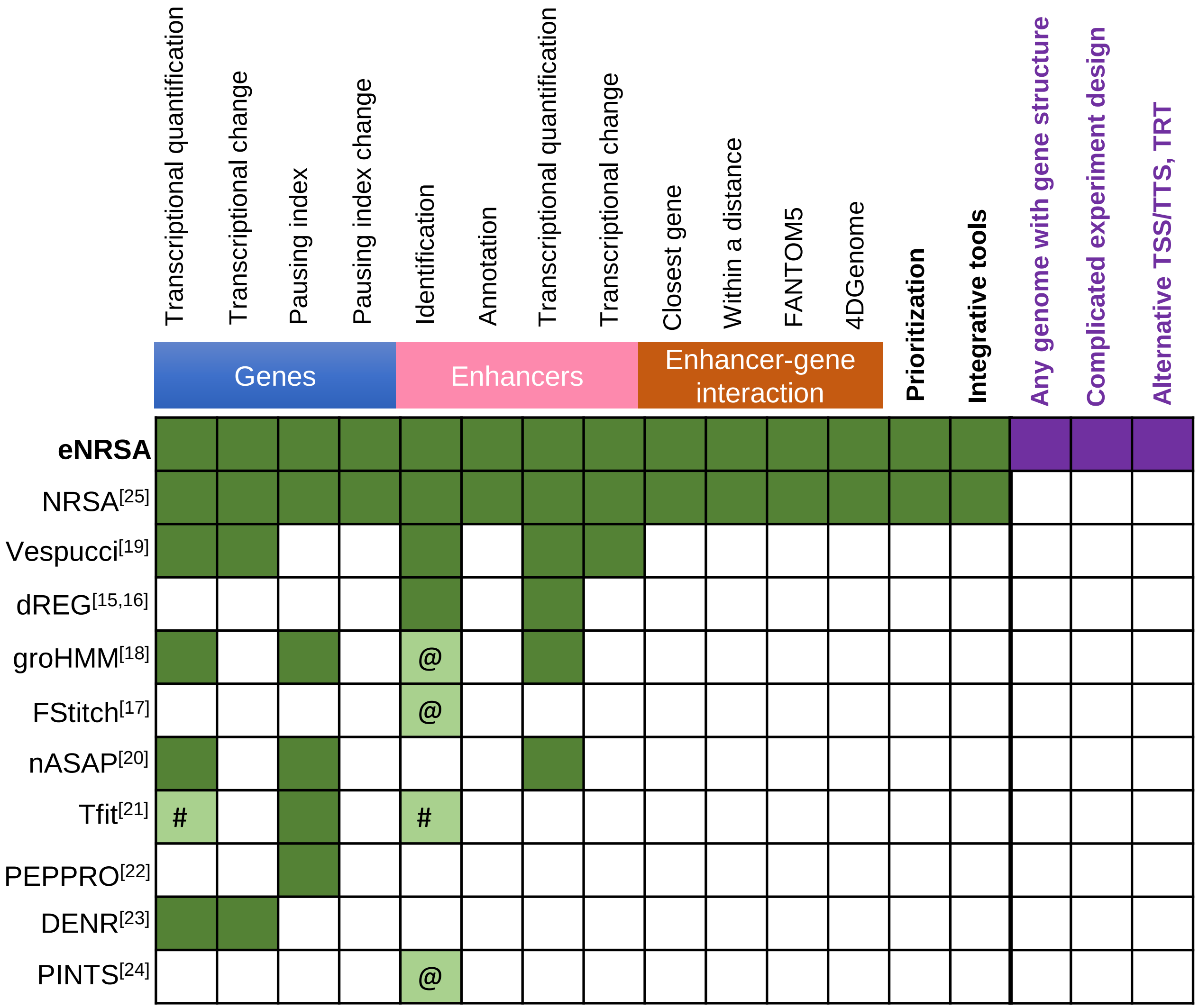

New features in eNRSA

@ identify novel transcripts instead of enhancers, but novel transcripts could be further classified into enhancers with the help of other scripts or genome annotation.

identify bidirectional transcription.

Fig. 2

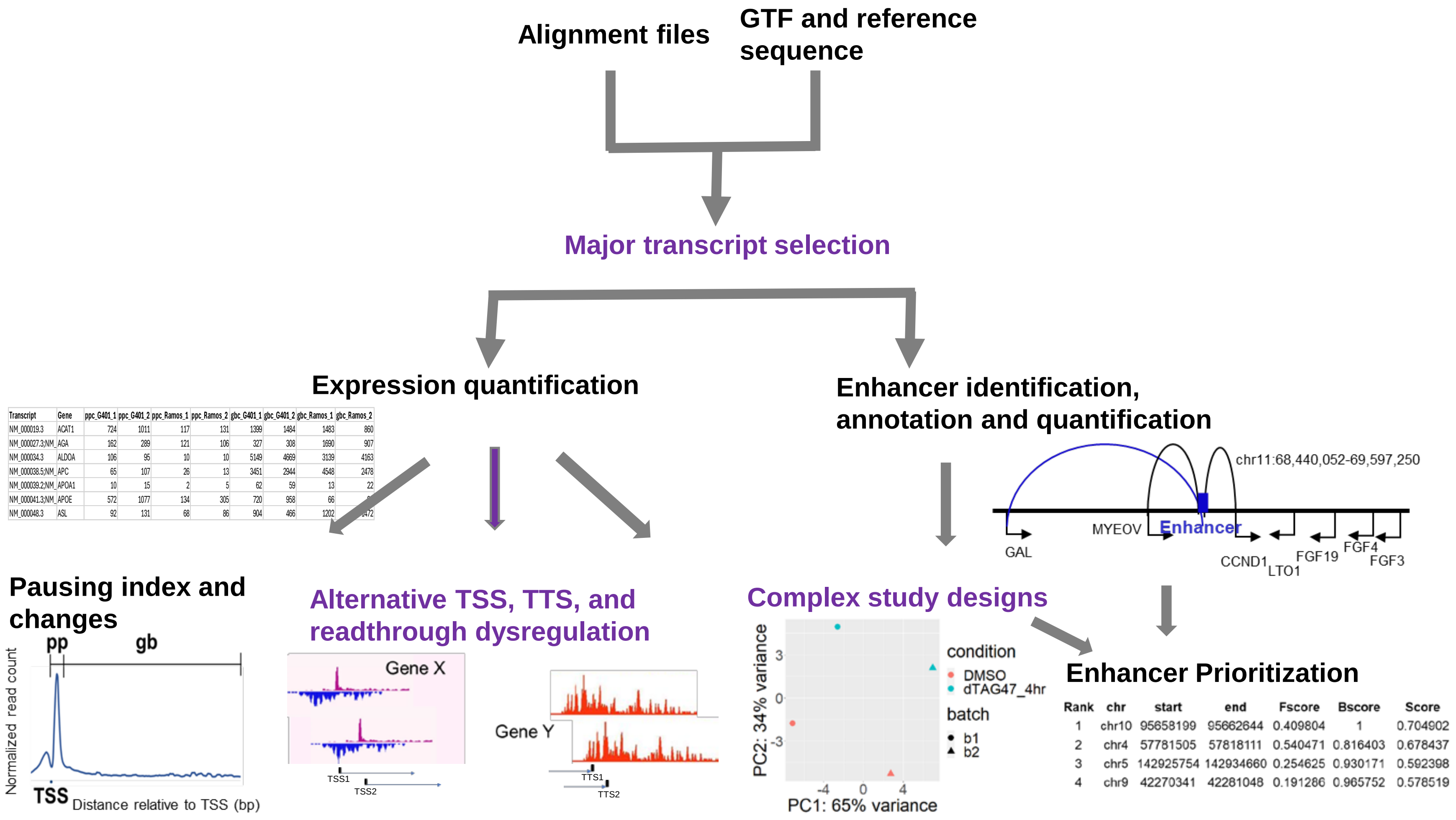

Alignment files
GTF and reference sequence
Major transcript selection
Expression quantification
Enhancer identification, annotation and quantification
Transcript Gene ppc_G401_1 ppc_G401_2 ppc_Ramos_1 ppc_Ramos_2 gbc_G401_1 gbc_G401_2 gbc_Ramos_1 gbc_Ramos_2
NM_000019.3 ACAT1 724 1011 117 131 1399 1484 1483 860
NM_000027.3;NM_ AGA 162 289 121 106 327 308 1690 907
NM_000034.3 ALDOA 106 95 10 10 5149 4669 3139 4163
NM_000038.5;NM_ APC 65 107 26 13 3451 2944 4548 2478
NM_000039.2;NM_ APOA1 10 15 2 5 62 59 13 22
NM_000041.3;NM_ APOE 572 1077 134 305 720 958 66
NM_000048.3 ASL 92 131 68 86 904 466 1202
chr11:68,440,052-69,597,250
GAL
MYEOV
Enhancer
CCND1
LTO1
FGF19
FGF4
FGF3
Pausing index and changes
pp
gb
Normalized read count
TSS
Distance relative to TSS (bp)
Alternative TSS, TTS, and readthrough dysregulation
Gene X
TSS1
TSS2
Gene Y
TTS1
TTS2
Complex study designs
PC2: 34% variance
PC1: 65% variance
condition
DMSO
dTAG47_4hr
batch
b1
b2
Enhancer Prioritization
Rank chr start end Fscore Bscore Score
1 chr10 95658199 95662644 0.409804 1 0.704902
2 chr4 57781505 57818111 0.540471 0.816403 0.678437
3 chr5 142925754 142934660 0.254625 0.930171 0.592398
4 chr9 42270341 42281048 0.191286 0.965752 0.578519

Fig. 3

A

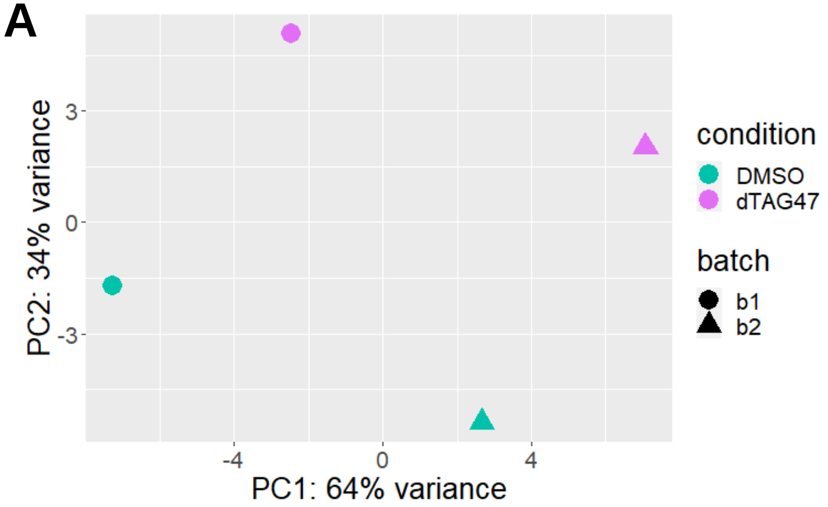

D

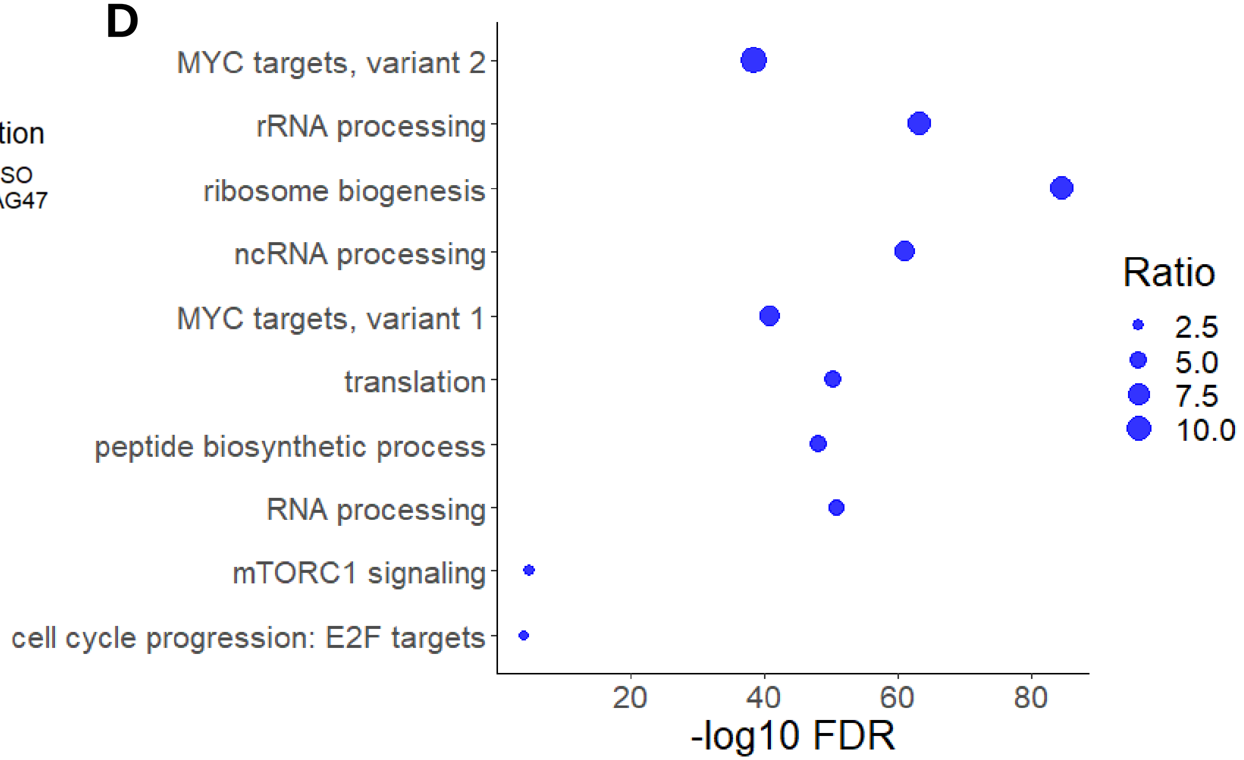

B

	NRSA	eNRSA
**Up**	5	287
**Down**	62	554

C

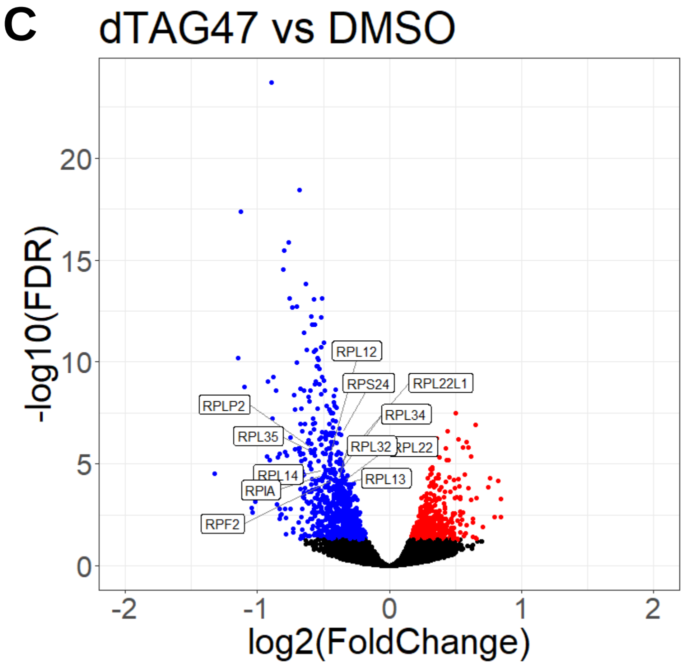

E

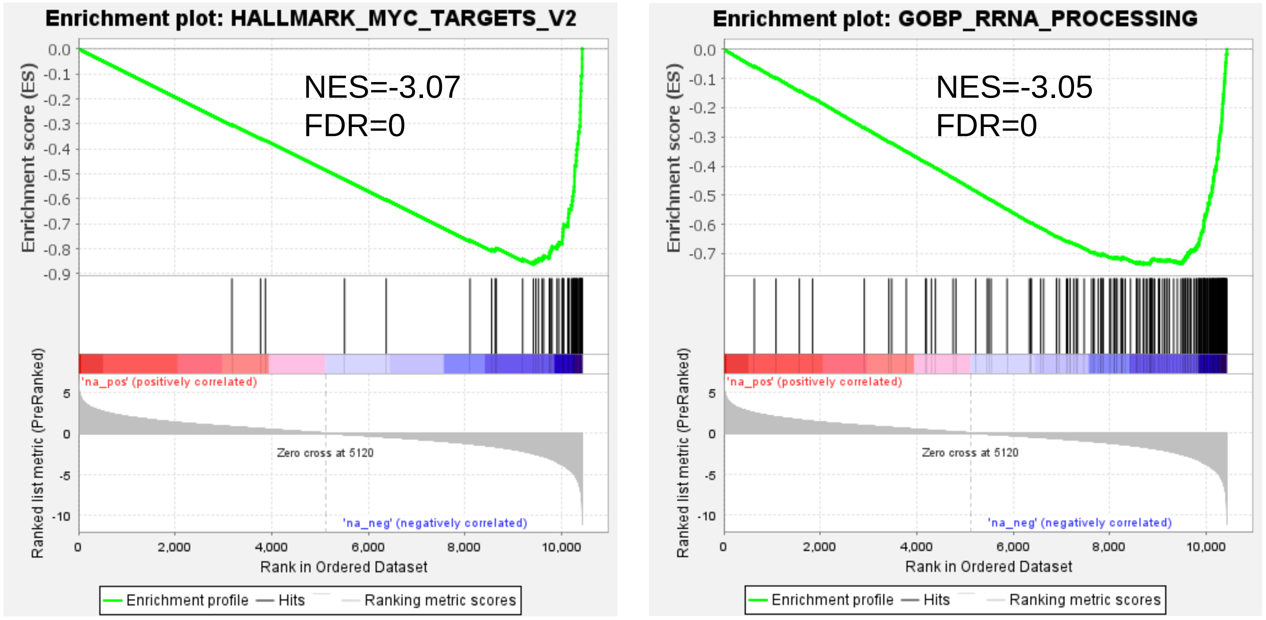

Fig. 4

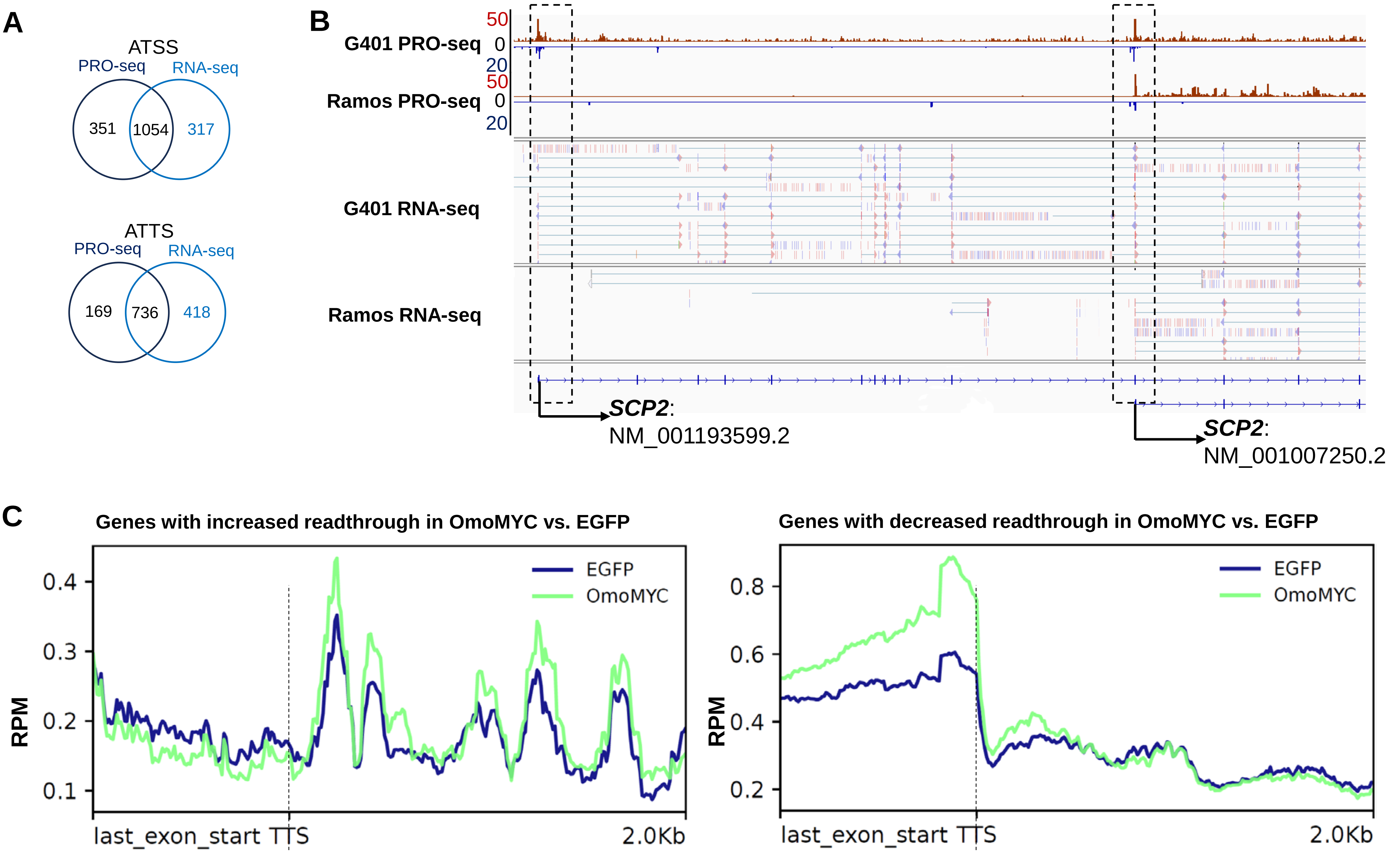
A
ATSS
PRO-seq
RNA-seq
351
1054
317
ATTS
PRO-seq
RNA-seq
169
736
418
B
G401 PRO-seq
Ramos PRO-seq
G401 RNA-seq
Ramos RNA-seq
50
0
20
SCP2: NM_001193599.2
SCP2: NM_001007250.2
C
Genes with increased readthrough in OmoMYC vs. EGFP
Genes with decreased readthrough in OmoMYC vs. EGFP
RPM
EGFP
OmoMYC
0.4
0.3
0.2
0.1
0.8
0.6
last_exon_start TTS
2.0Kb

Fig. 5

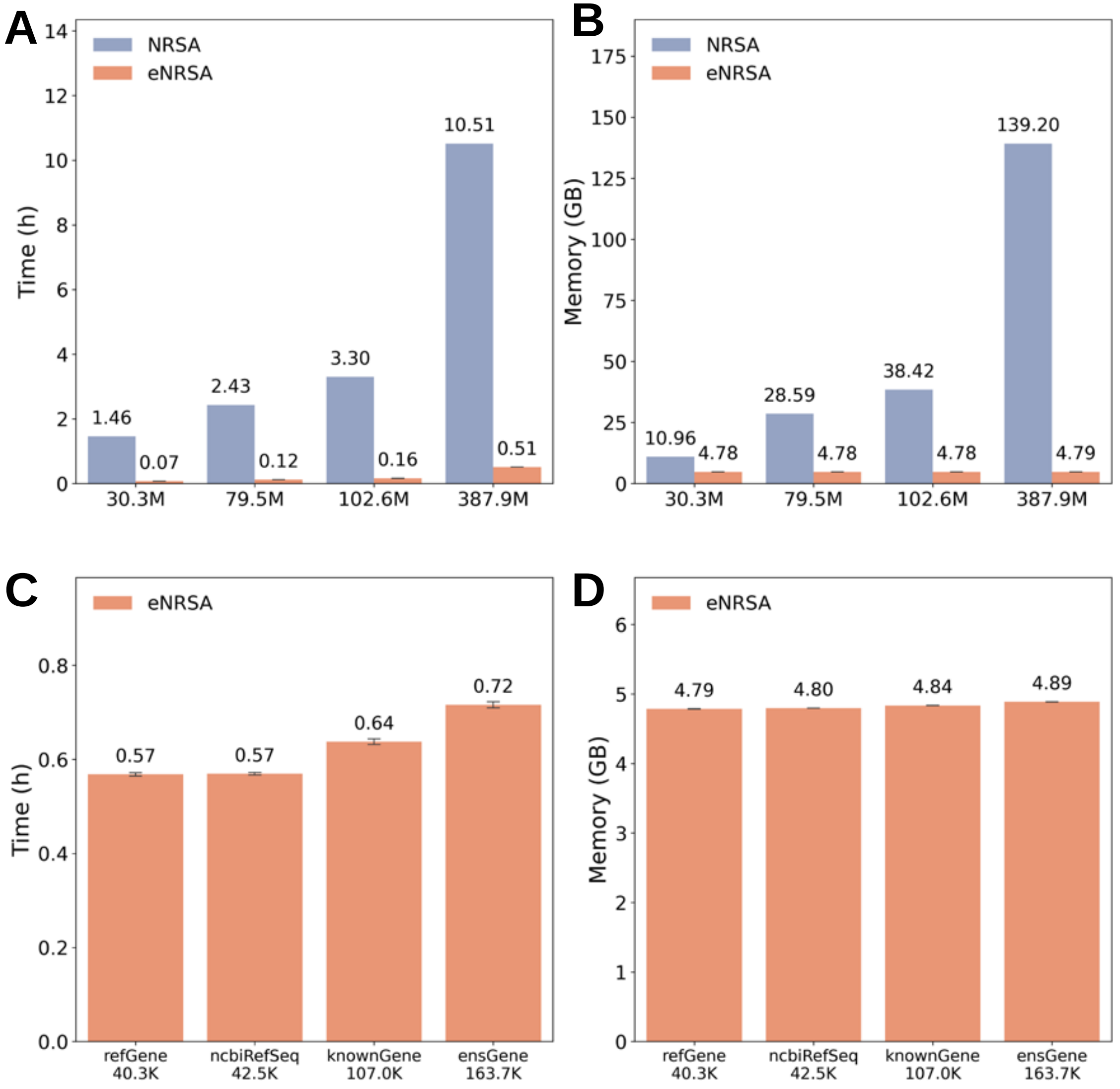
A
B
C
D
NRSA
eNRSA
Time (h)
Memory (GB)
1.46
0.07
2.43
0.12
3.30
0.16
10.51
0.51
10.96
4.78
28.59
4.78
38.42
4.78
139.20
4.79
30.3M
79.5M
102.6M
387.9M
0.57
0.57
0.64
0.72
4.79
4.80
4.84
4.89
refGene 40.3K
ncbiRefSeq 42.5K
knownGene 107.0K
ensGene 163.7K

Click here to access/download
Supplementary Material
eNRSA-SupplTables-revision.xlsx

Click here to access/download
Supplementary Material
eNRSA-Suppl-Figures-revision.pdf

Response to reviewers

Click here to access/download
Supplementary Material
GIGA-D-25-00028_response to reviewer_v2.docx

cover letter Click here to access/download;Personal Cover;cover letter-revision.docx

SCHOOL OF MEDICINE
VANDERBILT UNIVERSITY

Qi Liu, Professor
Vanderbilt University Medical Center
Department of Biostatistics
Technical Director of VANGARD
Tel: 615-322-6618
Email: qi.liu@vumc.org

GIGA-D-25-00028

eNRSA: A Faster and More Powerful Approach for Nascent Transcriptome Analysis

Dear Editor,

Thank you very much for giving us the opportunity to revise our manuscript entitled "**eNRSA: A Faster and More Powerful Approach for Nascent Transcriptome Analysis**" (**GIGA-D-25-00028**). Following your suggestion, we have registered eNRSA in the bio.tools and SciCrunch.org databases and have included RRID and biotoolsID identifiers in the revised manuscript.

We are deeply grateful to the two reviewers for their invaluable suggestions and insightful comments, which have significantly enhanced the manuscript. A detailed point-by-point response to the reviewers' comments has been provided.

We hope you and the reviewer will find that the manuscript has been significantly improved to meet the high standards of ***GigaScience***. We look forward to hearing back from you.

Thank you very much for your consideration of this manuscript!

Respectfully yours,
Qi Liu, PhD.
