# Supplementary material for: eNRSA: a faster and more powerful approach for nascent transcriptome analysis
Source: Gigascience. 2025 Jul 4;14:giaf071. doi: 10.1093/gigascience/giaf071 (PMC12231571; doi:10.1093/gigascience/giaf071)
Supplement: giaf071_Authors_Response_To_Reviewer_Comments_original_submission [file giaf071_authors_response_to_reviewer_comments_original_submission.pdf]

Author's Response To Reviewer Comments

GIGA-D-25-00028
eNRSA: A Faster and More Powerful Approach for Nascent Transcriptome Analysis

Reviewer #1
The manuscript by Wang et al reports a tool (eNRSA) for analysis of nascent transcriptomic data which can be generated by various methods such as GRO-seq, PRO-seq, TT-seq etc. This tool is advanced version of the NRSA tool which is already available. Along with the optimization in the functionality of NRSA authors have added new features which includes detection of Alternative Transcription Start site (ATSS), Alternative Transcription Termination Site (ATTS), inclusion of complex experimental design and flexibility to choose multiple organisms. The NRSA is already a great tool. This new tool will allow users to do analysis of nascent RNA transcriptome more efficiently even with limited computational resources. However there are few concerns which needs to be addressed to make the manuscript better and more comprehensive to the readers and the potential future users of the new tools.
Response: We greatly appreciate the reviewer's insightful comments and valuable suggestions. We have made every effort to address each point thoroughly. Specifically, we applied eNRSA to various nascent transcription datasets, including GRO-seq (Supplementary Table S6), mNET-seq (Supplementary Fig. S1), and Butt-seq (Supplementary Fig. S2), and proved the universal performance and robustness of eNRSA. We also validated ATSS and ATTS events identified by eNRSA through comparison with RNA-seq data (Fig. 4A, and Supplementary Tables S3 and S4). Furthermore, we have revised the manuscript to clarify methodological details regarding TSS/TTS site selection, readthrough calculation, and the rationale for using the Cochran-Mantel-Haenszel (CMH) test.

1. Authors have mentioned that this tool is compatible with nascent transcriptomic data generated by multiple experiments such as GRO-seq, PRO-seq, TT-seq, NET-seq etc but they have used only PRO-seq data for evaluating the tool. Authors should at least include data of few different types of experiments to prove the universal performance and robustness of their tool. Also they should mention the accuracy of the analysis for at least few different experiment types.
Response: We thank the reviewer for the great suggestion. In the revised manuscript, we have applied eNRSA to various types of nascent transcription experiments, including GRO-seq (Supplementary Table S6), mNET-seq (Supplementary Fig. S1), and Butt-seq (Supplementary Fig. S2), where results are consistent with the findings reported in the original publications [1-3] (Page 8). These applications further prove the universal performance and robustness of eNRSA. Furthermore, we discussed the appropriate interpretation of eNRSA results when analyzing data from other nascent protocols, such as SLAM-seq and TT-seq (Pages 7-8).
Since there is no gold standard for nascent transcriptomic data analysis, evaluating the accuracy of eNRSA is challenging. We first demonstrated that eNRSA produces consistent results with the findings reported in the original publications across various nascent transcriptome platforms (Supplementary Figs. S1 and S2 and Supplementary Table S6). Additionally, we permuted PRO-seq reads between DMSO and dTAG47-treated cells (100 permutations) to generate a background distribution, which was used to estimate the probability of obtaining the observed difference by chance. Using the permutation-based test as the gold standard, eNRSA achieved 80.1% sensitivity, 100% specificity, and 95.6% accuracy. We included this in the manuscript (Page 5).

2. Authors have mentioned that eNRSA can detect ATSS and ATTS. They have also mentioned that due to the nature of data of nascent transcriptome all ATSS and ATTS cannot be identified. Thus they should provide a more detailed comparison of the percentage of ATSS and ATTS identified with respect to isoform analysis performed using total RNA seq data of the same cell line or sample to show the robustness of the tool in this particular application.
Response: We thank the reviewer for this insightful suggestion. In response, we compared the ATSSs and ATTSs identified from the nascent transcriptome with those detected from RNA-seq data. Given the complexity of transcriptional regulation and the fact that nascent transcriptome and RNA-seq capture different layers of transcription, we observed a strong overlap between the two datasets. Specifically, of the 1,405 ATSSs identified from the nascent transcriptome, 1,054 (75.0%) were also supported by RNA-

seq. Similarly, of the 905 ATTSs detected in the nascent data, 736 (81.3%) were confirmed by RNA-seq (Fig. 4A in the manuscript). This strong concordance highlights the robustness of eNRSA in identifying ATSSs and ATTSs across transcriptomic layers. We have added this comparison to the manuscript (Fig. 4A, Pages 6 and 13).

3. Authors have mentioned in the method section that Homer package is required as one of the dependencies. That implies that the eRNA detection is done using the Homer package. Author should mention this in detail in the manuscript.
Response: We thank the reviewer for pointing this out. We have now included a detailed description of the eRNA detection and enhancer identification procedure using the HOMER package in the revised manuscript (Pages 10-11), to clarify its role as a dependency and how it is used in eNRSA.

4. In supplementary table S3 and S4 eNRSA have only compared the counts in the two alternate transcription start site (TSS) and Transcripion termination site (TTS) in two different condition. But in reality more than two TSS and TTS also exists. Although authors have mentioned this point as limitation but authors should clarify that if more than two TSS and TTS exists then on what basis two TSS and TTS will be chosen by the software?
Response: We apologize for the misunderstanding. eNRSA does not select only two TSS or TTS sites for comparison. Instead, for each gene, all TSS sites are considered, if they are separated by at least 1,000 bp to reduce signal interference and ensure reliable detection. For example, if a gene has three TSSs (TSS1, TSS2, TSS3), eNRSA evaluates usage shifts between all eligible pairs: TSS1 vs. TSS2, TSS1 vs. TSS3, and TSS2 vs. TSS3—excluding closely spaced TSSs (<1,000 bp apart). Similarly, for each gene, all TTS sites are considered, provided they do not overlap with any annotated gene and there is no gene within 3 kb downstream, to reduce transcriptional noise from neighboring genes.
In Supplementary Tables S3 and S4, multiple TSS and TTS shift events from the same gene are included. For instance, in Table S3, two ATSS events are reported for the RGS3 gene: one between chr9:116343157 and chr9:116356200, and another between chr9:116343157 and chr9:116356434.
We have clarified this in the revised manuscript (Page 11).

5. Authors have used read counts at TSS and TTS and used GTF to detect the major transcript and to identify ATSS and ATTS. The software is using Cochran-Mantel Haenszel test (CMH). Authors have mentioned very briefly about this test even in the method section. Authors should include some more points to justify the use of this test and why this will generate robust results.
Response: We thank the reviewer for the constructive comment. We have expanded the Methods section (Page 11) to provide a more detailed justification for the use of the Cochran-Mantel-Haenszel (CMH) test. Rather than pooling data across samples, which may obscure biological variability, we use the Cochran-Mantel-Haenszel (CMH) test to perform a stratified analysis. This test evaluates whether there is a consistent association between TSS usage and condition across sample pairs, while controlling for sample-specific variability. Under the assumption of a common odds ratio across strata, the CMH test increases statistical power and reduces bias due to heterogeneity among samples. This framework ensures robust detection of systematic shifts in TSS usage across biological replicates.

6. Authors have not mentioned the window size that they have used 50kb downstream of TTS to calculate readthrough dysregulation.
Response: We apologize for the confusion. In eNRSA, transcriptional readthrough is quantified as the ratio of the number of reads mapped within a fixed 50 kb region downstream of the annotated TTS to the number of reads mapped within the last exon [4]. A fixed 50 kb region was used, without applying any sliding window or variable window size. This approach was adapted from a previous study on transcriptional readthrough [4]. We have revised the Methods section to clarify this point (Pages 7, 12).
Minor concern 1. The figure 2 is repeated 2 times. PCA plot subfigure in figure 2 is cropped more than needed.
Response: We thank the reviewer for pointing this out. The duplicate Figure 2 has been removed, and the PCA plot in Figure 2 has been re-cropped to better display the data without excessive trimming.

Reviewer #2
In this article the authors create eNRSA, a pipeline for analysing nascent RNA sequening data. This pipeline

has clear benefits to the community as it is able to note changes in isoforms that are detectable in the data and take in complex experimental designs. The authors show it is also faster than their original pipeline. The main weaknesses are in a discussion of the 3’ end of the gene. However, the transcription termination site seems to be an all-encompassing term, with very little biological meaning. Other than this point the paper is well written and ready for publication.
Response: We deeply appreciate the reviewer for recognizing the value of eNRSA and for the insightful comments regarding its application to different nascent transcription protocols, the clear definition of TTS, and the detection of transcriptional readthrough. In response, we have discussed the appropriate interpretation of eNRSA results when analyzing various nascent transcription protocols. Additionally, we have revised the manuscript to clarify the definition of TTS and to highlight the performance of eNRSA in detecting readthrough events, particularly when polymerase dissociation fails.

Areas for Improvement
Major Issues:
• There are many Nascent RNA-seq protocols. Some of them are more “nascent” than others. For instance, since NET-seq is a pull-down of RNA polymerase, it is one of the most nascent. Similarly, PRO-seq is based on nt incorporated into the nascent RNA, and there is almost nonexistent time for cellular RNA degradation or splicing. It is one of the most nascent protocols. Protocols such as SLAM-seq, encorportate 4-thiouracil for 15 min with live cells, allowing the RNA-degradation machinery to act on transcripts in those 15 minutes. The longer the cells are incubated with 4-thiouracil, the more transcripts degrade. See the paper “A comparison of experimental assays and analytical methods for genome-wide identification of active enhancers” for more information. The authors should mention this variability as it relates to the differential results they get from their assay. For instance, I can imagine a scenario where GRO-seq could show similar 5’ ends between two samples, but SLAM-seq could show different 5’ ends as there is an RNA stability component to SLAM-seq. Therefore, the authors need to clarify how the choice of nascent protocol affects the interpretation of the eNRSA results.
Response: We thank the reviewer for the insightful comment. We have clarified the appropriate interpretation of eNRSA results when analyzing data from various nascent protocols in the manuscript (Pages 8-9 and below).
Although eNRSA is primarily designed to analyze GRO-seq or PRO-seq data, it can also be applied to data generated by other nascent RNA sequencing technologies that capture transcriptional dynamics, including pausing, elongation, and termination, such as Butt-seq, NET-seq, and mNET-seq. Examples of these applications are provided in Supplementary Table S6, Figs. S1 and S2, where the results are consistent with the findings reported in the original publications [1-3]. For nascent RNA sequencing technologies that capture only specific aspects of transcriptional dynamics, eNRSA results should be interpreted with caution. For instance, SLAM-seq tracks RNA kinetics including transcription, processing and degradation, and metabolically labeled RNAs are subject to degradation over time [5]. Therefore, while transcriptional changes within the gene body identified by eNRSA may still be meaningful, results related to pausing, ATSS/ATTS, and readthrough may not be interpretable—either because SLAM-seq does not capture pausing dynamics or due to RNA degradation leading to limited coverage beyond transcription end sites. In contrast, TT-seq maps the entire range of transient RNA and monitors RNA synthesis and degradation, which doesn’t yield peak signals near the promoter where polymerase pauses like GRO/PRO-seq, NET-seq, Butt-seq, or mNET-seq [6]. Therefore, eNRSA results on gene body, ATTS, and readthrough may be informative, but those related to pausing may not be applicable.

• The same issue of RNA stability is significant in their alternative TTS section. Moreover, it is entirely unclear what TTS means in this paper. From the RNA point of view, the cleavage position, which is just downstream of the PAS, is the transcript's end. This is also the annotated “end” of the gene. However, from the Pol II perspective, the position Pol II dissociates from the DNA is at the end of the transcript, and the dissociation position is 1-30kb downstream of the annotated end of the gene. Therefore, what the authors call “readthrough” is the typical and expected detection of reads downstream of the cleavage position. In PRO-seq assays, RNA in the readthrough region is expected, but less of these should be present in SLAM-seq assays as the RNA downstream of the cleavage position is quickly degreed in a typical cell. To publish this paper, the authors must clearly explain their definition of TTS and what it means biologically based on the nascent assay being used. There is a peak at the cleavage position, but a comparison of the cleavage and the dissociation positions shows that the most substantial peak is at the dissociation position. For more information, please see “TT-seq maps the human transient transcriptome”,

“FStitch: A fast and simple algorithm for detecting nascent RNA transcripts”, “Mechanisms of RNA Polymerase II Termination at the 3’-End of Genes”, “Knowing when to stop: Transcription termination on protein-coding genes by eukaryotic RNAPII”.
Response: We appreciate the reviewer’s comment and apologize for the lack of clarity regarding our definition of TTS. We have now clarified this in the revised manuscript (Page 6). Specifically, in eNRSA, TTS (transcription termination site) refers to the annotated end of the gene, defined as the 3’ end of the last exon in the GTF file, marking the cleavage and polyadenylation site of the mature RNA transcript.
As the reviewer pointed out, for a Polymerase II (Pol II) perspective, transcription termination does not occur immediately at this cleavage site. Instead, Pol II continues transcribing downstream, and dissociation typically occurs 1–50 kb downstream [4, 6, 7]. Therefore, eNRSA defines “readthrough” as transcriptionally engaged Pol II detected downstream of the annotated TTS/cleavage site and extending to the Pol II dissociation zone. This readthrough signal is expected in nascent transcription assays such as PRO-seq, GRO-seq, NET-seq, mNET-seq, Butt-seq, which capture engaged polymerase II, or TT-seq, which also maps transient RNA downstream of polyadenylation sites [6]. In contrast, assays like SLAM-seq, which detect metabolically labeled RNAs post-transcriptionally, are less likely to show signal downstream of the cleavage site due to rapid degradation of unprocessed or unstable transcripts in the readthrough region [5] (Page 7).

• When cells are stressed, such as heat shock or osmotic stress, cleavage fails, Polymerase dissociation fails, and some transcripts get far longer. The authors should demonstrate that eNSRA can detect this.
Response: We thank the reviewer for the great suggestion. In the revised manuscript, we demonstrate that eNRSA detects significant overall readthrough increase ($p<1.2e-12$) when termination factors (CPSF73 and CstF64+CstF64t) are depleted. This result has been included in the manuscript (Page 7 and Supplementary Fig. S1).

Minor Issues:
Figure 1 should include Peppro, Denr, Tfit and PINTS.
Response: We thank the reviewer for the suggestion. Peppro, Denr, Tfit, and PINTS have been included in Figure 1. We have also added brief introductions to these tools in the revised manuscript (Page 2).

1. Kaikkonen, M.U., et al., Control of VEGF-A transcriptional programs by pausing and genomic compartmentalization. Nucleic Acids Res, 2014. 42(20): p. 12570-84.
2. Yu, A.D. and M. Rosbash, Butt-seq: a new method for facile profiling of transcription. Genes Dev, 2023. 37(9-10): p. 432-448.
3. Nojima, T., et al., Mammalian NET-Seq Reveals Genome-wide Nascent Transcription Coupled to RNA Processing. Cell, 2015. 161(3): p. 526-540.
4. Gregersen, L.H., et al., SCAF4 and SCAF8, mRNA Anti-Terminator Proteins. Cell, 2019. 177(7): p. 1797-1813 e18.
5. Herzog, V.A., et al., Thiol-linked alkylation of RNA to assess expression dynamics. Nat Methods, 2017. 14(12): p. 1198-1204.
6. Schwalb, B., et al., TT-seq maps the human transient transcriptome. Science, 2016. 352(6290): p. 1225-8.
7. Lopez Martinez, D. and J.Q. Svejstrup, Mechanisms of RNA Polymerase II Termination at the 3'-End of Genes. J Mol Biol, 2025. 437(1): p. 168735.

Editorial Manager®

Cookie Preference Center

We use cookies which are necessary to make our site work. We may also use additional cookies to analyze, improve and personalize our content and your digital experience. For more information, see our Cookie Policy.

You may choose not to allow some types of cookies. However, blocking some types may impact your experience of our site and the services we are able to offer. See the different category headings below to find out more or change your settings.

Allow all

Manage Consent Preferences

Strictly Necessary Cookies

Always active

These cookies are necessary for the website to function and cannot be switched off in our systems. They are usually only set in response to actions made by you which amount to a request for services, such as setting your privacy preferences, logging in or filling in forms. You can set your browser to block or alert you about these cookies, but some parts of the site will not then work.

Functional Cookies

These cookies enable the website to provide enhanced functionality and personalisation. They may be set by us or by third party providers whose services we have added to our pages. If you do not allow these cookies then some or all of these services may not function properly.

Performance Cookies

These cookies allow us to count visits and traffic sources so we can measure and improve the performance of our site. They help us to know which pages are the most and least popular and see how visitors move around the site.

Targeting Cookies

These cookies may be set through our site by our advertising partners. They may be used by those companies to build a profile of your interests and show you relevant adverts on other sites. If you do not allow these cookies, you will experience less targeted advertising.

Cookie List

Clear

Apply Cancel
Consent Leg.Interest

Confirm my choices

Powered by onetrust
