# Supplementary material for: eNRSA: a faster and more powerful approach for nascent transcriptome analysis
Source: Gigascience. 2025 Jul 4;14:giaf071. doi: 10.1093/gigascience/giaf071 (PMC12231571; doi:10.1093/gigascience/giaf071)
Supplement: giaf071_Reviewer_1_Report_Original_submission — Aniket Kumar -- 2/7/2025 [file giaf071_reviewer_1_report_original_submission.pdf]

Reviewer Report

Title: eNRSA: A Faster and More Powerful Approach for Nascent Transcriptome Analysis

Version: Original Submission **Date:** 2/7/2025

Reviewer name: Aniket Kumar

Reviewer Comments to Author:

The manuscript by Wang et al reports a tool (eNRSA) for analysis of nascent transcriptomic data which can be generated by various methods such as GRO-seq, PRO-seq, TT-seq etc. This tool is advanced version of the NRSA tool which is already available. Along with the optimization in the functionality of NRSA authors have added new features which includes detection of Alternative Transcription Start site (ATSS), Alternative Transcription Termination Site (ATTS), inclusion of complex experimental design and flexibility to choose multiple organisms. The NRSA is already a great tool. This new tool will allow users to do analysis of nascent RNA transcriptome more efficiently even with limited computational resources. However there are few concerns which needs to be addressed to make the manuscript better and more comprehensive to the readers and the potential future users of the new tools.1. Authors have mentioned that this tool is compatible with nascent transcriptomic data generated by multiple experiments such as GRO-seq, PRO-seq, TT-seq, NET-seq etc but they have used only PRO-seq data for evaluating the tool. Authors should atleast include data of few different types of experiments to prove the universal performance and robustness of their tool. Also they should mention the accuracy of the analysis for atleast few different experiment types.2. Authors have mentioned that eNRSA can detect ATSS and ATTS. They have also mentioned that due to the nature of data of nascent transcriptome all ATSS and ATTS cannot be identified. Thus they should provide a more detailed comparison of the percentage of ATSS and ATTS identified with respect to isoform analysis performed using total RNA seq data of the same cell line or sample to show the robustness of the tool in this particular application.3. Authors have mentioned in the method section that Homer package is required as one of the dependencies. That implies that the eRNA detection is done using the Homer package. Author should mention this in detail in the manuscript.4. In supplementary table S3 and S4 eNRSA have only compared the counts in the two alternate transcription start site (TSS) and Transcripion termination site (TTS) in two different condition. But in reality more than two TSS and TTS also exists. Although authors have mentioned this point as limitation but authors should clarify that if more than two TSS and TTS exists then on what basis two TSS and TTS will be chosen by the software?5. Authors have used read counts at TSS and TTS and used GTF to detect the major transcript and to identify ATSS and ATTS. The software is using Cochran-Mantel Haenszel test (CMH). Authors have mentioned very briefly about this test even in the method section. Authors should include some more points to justify the use of this test and why this will generate robust results.6. Authors have not mentioned the window size that they have used 50kb downstream of TTS to calculate readthrough dysregulation.Minor concern1. The figure 2 is repeated 2 times. PCA plot subfigure in figure 2 is cropped more than needed.

Level of Interest

Please indicate how interesting you found the manuscript: Choose an item.

Quality of Written English

Please indicate the quality of language in the manuscript: Choose an item.

Declaration of Competing Interests

Please complete a declaration of competing interests, considering the following questions:

- Have you in the past five years received reimbursements, fees, funding, or salary from an organisation that may in any way gain or lose financially from the publication of this manuscript, either now or in the future?
- Do you hold any stocks or shares in an organisation that may in any way gain or lose financially from the publication of this manuscript, either now or in the future?
- Do you hold or are you currently applying for any patents relating to the content of the manuscript?
- Have you received reimbursements, fees, funding, or salary from an organization that holds or has applied for patents relating to the content of the manuscript?
- Do you have any other financial competing interests?
- Do you have any non-financial competing interests in relation to this paper?

If you can answer no to all of the above, write 'I declare that I have no competing interests' below. If your reply is yes to any, please give details below.

I declare that I have no competing interests

I agree to the open peer review policy of the journal. I understand that my name will be included on my report to the authors and, if the manuscript is accepted for publication, my named report including any attachments I upload will be posted on the website along with the authors' responses. I agree for my report to be made available under an Open Access Creative Commons CC-BY license (http://creativecommons.org/licenses/by/4.0/). I understand that any comments which I do not wish to be included in my named report can be included as confidential comments to the editors, which will not be published.

Choose an item.

To further support our reviewers, we have joined with Publons, where you can gain additional credit to further highlight your hard work (see: https://publons.com/journal/530/gigascience). On publication of this paper, your review will be automatically added to Publons, you can then choose whether or not to claim your Publons credit. I understand this statement.

Yes Choose an item.
