# Supplementary material for: eNRSA: a faster and more powerful approach for nascent transcriptome analysis
Source: Gigascience. 2025 Jul 4;14:giaf071. doi: 10.1093/gigascience/giaf071 (PMC12231571; doi:10.1093/gigascience/giaf071)
Supplement: giaf071_Reviewer_2_Report_Original_submission — Mary Allen, Ph.D. -- 3/25/2025 [file giaf071_reviewer_2_report_original_submission.pdf]

Reviewer Report

Title: eNRSA: A Faster and More Powerful Approach for Nascent Transcriptome Analysis

Version: Original Submission **Date:** 3/25/2025

Reviewer name: Mary Allen, Ph.D.

Reviewer Comments to Author:

In this article the authors create eNRSA, a pipeline for analysing nascent RNA sequening data. This pipeline has clear benefits to the community as it is able to note changes in isoroms that are detectable in the data and take in complex experimental designs. The authors show it is also faster than their original pipeline. The main weaknesses are in a discussion of the 3' end of the gene. Transcription termination site seems to be a all encompansing term, with very little biological meaning. Other than this point the paper is well written and ready for publication.Areas for Improvement* Major Issues:o There are many Nascent RNA-seq protocols. Some of them are more "nascent" than others. For instance, since NET-seq is a pull-down of RNA polymerase, it is one of the most nascent. Similarly, PRO-seq is based on nt incorporated into the nascent RNA, and there is almost nonexistent time for cellular RNA degradation or splicing. It is one of the most nascent protocols. Protocols such as SLAM-seq, encorportate 4-thiouracil for 15 min with live cells, allowing the RNA-degradation machinery to act on transcripts in those 15 minutes. The longer the cells are incubated with 4-thiouracil, the more transcripts degrade. See the paper "A comparison of experimental assays and analytical methods for genome-wide identification of active enhancers" for more information. The authors should mention this variability as it relates to the differential results they get from their assay. For instance, I can imagine a scenario where GRO-seq could show similar 5' ends between two samples, but SLAM-seq could show different 5' ends as there is an RNA stability component to SLAM-seq. Therefore, the authors need to note how the choice of nascent protocol affects the interpretation of the eNRSA results.o The same issue of RNA stability is significant in their alternative TTS section. Moreover, it is entirely unclear what TTS means in this paper. From the RNA point of view, the cleavage position, which is just downstream of the PAS, is the transcript's end. This is also the annotated "end" of the gene. However, from the Pol II perspective, the position Pol II dissociates from the DNA is at the end of the transcript, and the dissociation position is 1-30kb downstream of the annotated end of the gene. Therefore, what the authors call "readthrough" is the typical and expected detection of reads downstream of the cleavage position. In PRO-seq assays, RNA in the readthrough region is expected, but less of these should be present in SLAM-seq assays as the RNA downstream of the cleavage position is quickly degreed in a typical cell. To publish this paper, the authors must clearly explain their definition of TTS and what it means biologically based on the nascent assay being used. There is a peak at the cleavage position, but a comparison of the cleavage and the dissociation positions shows that the most substantial peak is at the dissociation position. For more information, please see "TT-seq maps the human transient transcriptome", "FStitch: A fast and simple algorithm for detecting nascent RNA transcripts", "Mechanisms of RNA Polymerase II Termination at the 3â€²-End of Genes", "Knowing when to stop: Transcription termination on protein-coding genes by eukaryotic RNAPII",o When cells are stressed, such as heat shock or osmotic stress, cleavage fails,

Polymerase dissociation fails, and some transcripts get far longer. The authors should demonstrate that eNSRA can detect this.* Minor Issues:o Figure 1 should include Peppro, Denr, Tfit and PINTS.

Level of Interest

Please indicate how interesting you found the manuscript: Choose an item.

Quality of Written English

Please indicate the quality of language in the manuscript: Choose an item.

Declaration of Competing Interests

Please complete a declaration of competing interests, considering the following questions:

- Have you in the past five years received reimbursements, fees, funding, or salary from an organisation that may in any way gain or lose financially from the publication of this manuscript, either now or in the future?
- Do you hold any stocks or shares in an organisation that may in any way gain or lose financially from the publication of this manuscript, either now or in the future?
- Do you hold or are you currently applying for any patents relating to the content of the manuscript?
- Have you received reimbursements, fees, funding, or salary from an organization that holds or has applied for patents relating to the content of the manuscript?
- Do you have any other financial competing interests?
- Do you have any non-financial competing interests in relation to this paper?

If you can answer no to all of the above, write 'I declare that I have no competing interests' below. If your reply is yes to any, please give details below.

I declare that I have no competing interests

I agree to the open peer review policy of the journal. I understand that my name will be included on my report to the authors and, if the manuscript is accepted for publication, my named report including any attachments I upload will be posted on the website along with the authors' responses. I agree for my report to be made available under an Open Access Creative Commons CC-BY license (http://creativecommons.org/licenses/by/4.0/). I understand that any comments which I do not wish to be included in my named report can be included as confidential comments to the editors, which will not be published.

Choose an item.

To further support our reviewers, we have joined with Publons, where you can gain additional credit to further highlight your hard work (see: https://publons.com/journal/530/gigascience). On publication of this paper, your review will be automatically added to Publons, you can then choose whether or not to claim your Publons credit. I understand this statement.

Yes Choose an item.
